# Accommodation‐based interventions for individuals experiencing, or at risk of experiencing, homelessness

**DOI:** 10.1002/cl2.1165

**Published:** 2021-05-18

**Authors:** Ciara Keenan, Sarah Miller, Jennifer Hanratty, Terri Pigott, Jayne Hamilton, Christopher Coughlan, Peter Mackie, Suzanne Fitzpatrick, John Cowman

**Affiliations:** ^1^ Campbell UK & Ireland, Centre for Evidence and Social Innovation Queen's University Belfast UK; ^2^ School of Public Health Georgia State University Atlanta Georgia USA; ^3^ Information Services Cardiff University Cardiff UK; ^4^ Heriot‐Watt University Edinburgh UK; ^5^ Department of Social Work Health Service Executive Dublin Ireland

## Abstract

**Background:**

Globally, almost 1.6 billion individuals lack adequate housing. Many accommodation‐based approaches have evolved across the globe to incorporate additional support and services beyond delivery of housing.

**Objectives:**

This review examines the effectiveness of accommodation‐based approaches on outcomes including housing stability, health, employment, crime, wellbeing, and cost for individuals experiencing or at risk of experiencing homelessness.

**Search Methods:**

The systematic review is based on evidence already identified in two existing EGMs commissioned by the Centre for Homelessness Impact (CHI) and built by White et al. The maps were constructed using a comprehensive three stage search and mapping process. Stage one mapped included studies in an existing systematic review on homelessness, stage two was an extensive search of 17 academic databases, three EGM databases, and eight systematic review databases. Finally stage three included web searches for grey literature, scanning reference lists of included studies and consultation with experts to identify additional literature. We identified 223 unique studies across 551 articles from the effectiveness map on 12th April 2019.

**Selection Criteria:**

We include research on all individuals currently experiencing, or at risk of experiencing homelessness irrespective of age or gender, in high‐income countries. The Network Meta‐Analysis (NMA) contains all study designs where a comparison group was used. This includes randomised controlled trials (RCTs), quasi‐experimental designs, matched comparisons and other study designs that attempt to isolate the impact of the intervention on homelessness. The NMA primarily addresses how interventions can reduce homelessness and increase housing stability for those individuals experiencing, or at risk of experiencing, homelessness. Additional outcomes are examined and narratively described. These include: access to mainstream healthcare; crime and justice; employment and income; capabilities and wellbeing; and cost of intervention. These outcomes reflect the domains used in the EGM, with the addition of cost.

**Data Collection and Analysis:**

Due to the diverse nature of the literature on accommodation‐based approaches, the way in which the approaches are implemented in practice, and the disordered descriptions of the categories, the review team created a novel typology to allow meaningful categorisations for functional and useful comparison between the various intervention types. Once these eligible categories were identified, we undertook dual data extraction, where two authors completed data extraction and risk of bias (ROB) assessments independently for each study. NMA was conducted across outcomes related to housing stability and health.Qualitative data from process evaluations is included using a “Best Fit” Framework synthesis. The purpose of this synthesis is to complement the quantitative evidence and provide a better understanding of what factors influenced programme effectiveness. All included Qualitative data followed the initial framework provided by the five main analytical categories of factors of influence (reflected in the EGM), namely: contextual factors, policy makers/funders, programme administrators/managers/implementing agencies, staff/case workers and recipients of the programme.

**Main Results:**

There was a total of 13,128 people included in the review, across 51 reports of 28 studies. Most of the included studies were carried out in the United States of America (25/28), with other locations including Canada and the UK. Sixteen studies were RCTs (57%) and 12 were nonrandomised (quasi‐experimental) designs (43%). Assessment of methodological quality and potential for bias was conducted using the second version of the Cochrane Risk of Bias tool for Randomised controlled trials. Nonrandomised studies were coded using the ROBINS‐ I tool. Out of the 28 studies, three had sufficiently low ROB (11%), 11 (39%) had moderate ROB, and five (18%) presented serious problems with ROB, and nine (32%) demonstrated high, critical problems with their methodology. A NMA on housing stability outcomes demonstrates that interventions offering the highest levels of support alongside unconditional accommodation (High/Unconditional) were more effective in improving housing stability compared to basic support alongside unconditional housing (Basic/Unconditional) (ES=1.10, 95% confidence interval [CI] [0.39, 1.82]), and in comparison to a no‐intervention control group (ES=0.62, 95% CI [0.19, 1.06]). A second NMA on health outcomes demonstrates that interventions categorised as offering Moderate/Conditional (ES= 0.36, 95% CI [0.03, 0.69]) and High/Unconditional (ES = 0.22, 95% CI [0.01, 0.43]) support were effective in improving health outcomes compared to no intervention. These effects were smaller than those observed for housing stability. The quality of the evidence was relatively low but varied across the 28 included studies. Depending on the context, finding accommodation for those who need it can be hindered by supply and affordability in the market. The social welfare approach in each jurisdiction can impact heavily on support available and can influence some of the prejudice and stigma surrounding homelessness. The evaluations emphasised the need for collaboration and a shared commitment between policymakers, funders and practitioners which creates community and buy in across sectors and agencies. However, co‐ordinating this is difficult and requires sustainability to work. For those implementing programmes, it was important to invest time in developing a culture together to build trust and solid relationships. Additionally, identifying sufficient resources and appropriate referral routes allows for better implementation planning. Involving staff and case workers in creating processes helps drive enthusiasm and energy for the service. Time should be allocated for staff to develop key skills and communicate engage effectively with service users. Finally, staff need time to develop trust and relationships with service users; this goes hand in hand with providing information that is up to date and useful as well making themselves accessible in terms of location and time.

**Authors' Conclusions:**

The network meta‐analysis suggests that all types of accommodation which provided support are more effective than no intervention or Basic/Unconditional accommodation in terms of housing stability and health. The qualitative evidence synthesis raised a primary issue in relation to context: which was the lack of stable, affordable accommodation and the variability in the rental market, such that actually sourcing accommodation to provide for individuals who are homeless is extremely challenging. Collaboration between stakeholders and practitioners can be fruitful but difficult to coordinate across different agencies and organisations.

## 1 PLAIN LANGUAGE SUMMARY

### 1.1 Accommodation‐based approaches help people remain healthy and stably housed

Accommodation‐based approaches are mostly effective for increasing housing stability and health outcomes, except for those which offer low support housing without behavioural conditions. These approaches led to worse outcomes related to housing stability and health than receiving nothing at all. Agencies working together and sharing resources such as time and staff creates a commitment to ending homelessness.

#### 1.1.1 What is this review about?

Globally, almost 1.6 billion individuals lack adequate housing. Many accommodation‐based approaches have evolved to incorporate support and services beyond delivery of housing. This review looked at whether these approaches are effective on outcomes including housing stability, health, employment, crime, wellbeing, and cost for individuals experiencing or at risk of experiencing homelessness.

**What is the aim of this review?**

This Campbell systematic review of qualitative and quantitative evidence examines how useful accommodation‐based approaches are for people experiencing homelessness. The quantitative data summarises evidence from 28 studies, reported in 51 articles, mainly from North America. The qualitative data summarises evidence from 10 articles from high‐income countries.

#### 1.1.2 What studies are included?

The quantitative research provides an overview of effectiveness findings from 28 intervention studies reported in 51 articles of accommodation‐based interventions. Twenty‐five out of the 28 studies are from the United States, two from Canada and one from the UK. The quality of the research is generally low and represents important weaknesses in the evidence base.

The qualitative data presents one evaluation based on an intervention conducted in the UK, two in Ireland, one in Australia, one across Europe and the remaining five carried out in North America; three in the United States and two in Canada. The quality of the evaluations was average and did not directly evaluate the effectiveness interventions discussed in this review.

#### 1.1.3 Do accommodation‐based approaches help people experiencing homelessness?

Interventions which provide the highest levels of support and do not place rules on the person receiving the intervention are best at improving housing stability and health outcomes.

Interventions which offer the lowest levels of support and do not place rules on the person might harm those individuals. For those individuals, housing stability and health outcomes were worse than for all other interventions, including individuals who are not receiving any intervention at all.

#### 1.1.4 What implementation factors affect how well accommodation‐based approaches work?

Staff, resources and time often impacted the delivery of accommodation programmes most. Programme managers knew that members of staff working on the ground took initiative and were capable in their roles. However, they need adequate training and time to build good relations with service users.

There is a tension in funding allocated between new and established services, which can cause issues when services collaborate. It can also impact upon the shared commitment to ending homelessness. Buy‐in at all levels of influence can impact how successful a programme is and how many people experiencing homelessness it can engage with.

#### 1.1.5 What do the findings of this review mean?

Those interventions which are described as Basic/Unconditional (i.e., those that only satisfy very basic human needs such as a bed and food) harm people: meaning they had worse health and housing stability outcomes even when compared to receiving nothing at all. This invites questions on whether these types of accommodation‐based interventions should be discontinued so that other more suitable and effective offers of support can be made available.

Too few studies assess the cost, or important participant characteristics like age and gender. There are also gaps related to where the research is conducted. Most of the studies included are from the United States and Canada which have very different social welfare systems to those of the UK. The process evaluations were conducted in high‐income countries with different housing contexts and social welfare systems.

The studies were of average quality and not connected to the effectiveness studies, which presented issues when drawing connections between the available data. Researchers conducting studies into accommodation‐based interventions should consider evaluating and publishing the factors impacting upon the trial, reflecting on why the intervention did or did not work, and for whom.

#### 1.1.6 How up‐to‐date is this review?

Quantitative studies were downloaded from the effectiveness Evidence and Gap Map on 12 April 2019. Qualitative reports were downloaded from the Process and Implementation Evidence and Gap Map on 10 May 2019.

## 2 BACKGROUND

### 2.1 The problem, condition or issue

Homelessness affects individuals who are experiencing life without safe, adequate, or stable housing. Conceived in this way, homelessness not only describes those individuals who are visibly homeless and living on the street, but also those precariously housed individuals who; stay in emergency accommodation, sleep in crowded or inadequate housing, and those who are not safe in their living environment. FEANTSA further classify individuals experiencing homelessness as those who are roofless, those who are houseless and those who experience insecure or inadequate housing (FEANTSA, 2005).

Global data suggests that at least 1.6 billion people lack adequate housing (Habitat for Humanity, 2017). In the European context this figure continues to rise across all European Union member states except for Finland where homelessness has been on the decline since 1987 (FEANTSA, 2017; Y‐Foundation, 2017). Crisis, a charity based in the UK, estimated that in 2019 England acknowledged 57,890 households as homeless. In Wales, homelessness threatened 9,210 households and in Scotland, 34,100 individual applications were assessed for homelessness status (Fitzpatrick et al., 2016). Finally, Northern Ireland have an estimated 18,200 households experiencing homelessness according to a recent report (Fitzpatrick et al., 2020).

Without access to adequate housing, individuals experience multiple adverse effects including; exposure to disease, poverty, isolation, mental health issues, prejudice and discrimination, and are under constant and significant threat to their personal safety. Therefore, having access to safe, stable and adequate housing is internationally recognised as a basic human right (OHCHR, 2009) and is central to create the conditions whereby the population can live healthy, safe and happy lives.

### 2.2 The intervention

Homelessness is recognised as a multifaceted issue and many accommodation‐based approaches have evolved across the globe to incorporate additional support and services beyond delivery of housing, while other interventions deliver only temporary housing which is insufficient to meet people's basic needs. Through amalgamation of global ideas, the progression of evidence‐based policy and practice, and further establishment of welfare states, classification of accommodation‐based approaches is varied and represents the diversity in how the interventions were formed. The number of interventions which now exist, coupled with inconsistent descriptions of interventions and their elements (e.g., different models of housing, support services, expectations of engagement, etc.), has rendered current categorisations meaningless. Therefore, it was deemed necessary to group interventions based on their components, rather than their name. Later in this review, we describe how the review team created a novel and meaningful typology to categorise included interventions, however, initially we will briefly describe some of the familiar interventions that establish this evidence base.

#### 2.2.1 Housing First

Housing First interventions offer housing to people experiencing homelessness with minimal obligation or preconditions being placed upon the participant. The Housing First programme, as conceived by Tsemberis (Tsemberis & Eisenberg, 2000), had clear principles which other researchers have since deviated from. However, most Housing First programmes share some common themes: (i) the participant is provided access to permanent housing immediately, without conditions, (ii) decisions around the location of the home and the services received are made by the user, (iii) support and services to aid the individual recovery are offered alongside housing placement, (iv) social integration with local community and meaningful engagement with positive activities is encouraged. Housing First is based on the principle that housing should be made available in the first instance and preconditions such as sobriety and involvement in treatment programmes are unnecessary barriers placed upon people who are homeless. Through the removal of these common obstacles, it is believed that the individual has a better chance of achieving stabilisation in appropriate housing and feeling more willing or able to accept treatment. In the original Pathways model of Housing First, housing provision is offered through scattered sites, which is where user choice is emphasised and housing is distributed (scattered) among existing rental properties. A key variation in the model has been the use of congregate housing where a property is reserved solely for the use of individuals experiencing homelessness. There is significant debate about the potential differences in effectiveness of these two models (Mackie et al., 2017).

#### 2.2.2 Rapid rehousing

The rapid rehousing approach seeks to provide accommodation to individuals experiencing homelessness as quickly as possible. Generally, the rapid rehousing approach will identify available accommodation, aid with application, rent and moving in and the provision of case management to support access to other services. Rapid rehousing might provide the service user with a short‐term subsidy to assist with rent, rent in advance, help with rent arrears or help with moving. Generally, rapid rehousing targets those persons experiencing homelessness who have lower support needs and are less likely to require substantial access to services. The amount of support provided through a rapid rehousing approach is usually time limited.

#### 2.2.3 Hostels

Hostels provide accommodation to meet short‐term housing needs. Homeless hostels often impose strict rules on the people who stay there relating to abstinence, behaviour and curfews. The individuals who use hostels vary but may include individuals, including those with pets, families and couples who are homeless. There is no clear definition on what constitutes a hostel and the provision offered will vary across councils, counties, and countries. Sleeping arrangements are variable too, with some offering dormitory style sleeping alongside communal kitchen, living, and shower areas while others have bedsit flats. The type of support offered by a homeless hostel is often determined by the resources available and individuals they can house. There are examples of in‐house support services such as: residents having a support plan to move to more stable accommodation; practical help with form filling and obtaining necessary governmental documents to continue education or gain employment; or treatment for substance abuse or mental health issues. This support is sometimes provided by other outside organisations separate to the hostel.

#### 2.2.4 Shelters

Homeless shelters are typically viewed as a basic form of temporary accommodation where a bed is provided in a shared space overnight which a requirement for the individual to vacate the space during the day. One of the key features of a homeless shelter is that it is transitory and not usually seen as a stable form of accommodation as the individual are often in overcrowded buildings, and often subjected to physical altercations, theft, substance abuse, and unhygienic sleeping conditions. Like hostels, homeless shelters often place additional requirements on potential users including night‐time curfews. Additional services that may or may not be provided by the homeless shelter are warm meals for dinner and breakfast or support from volunteers and staff who help individuals make connections to other services. However, similarly to hostels, some support may be offered by external organisations and not by the shelter itself. Shelters and hostels are often defined in different ways in the UK and the United States, where these models are often used. Even within these categories there is substantial variability on the services that are provided and the conditions in which the facilities operate. Due to some of the common elements between shelters and hostels, which have now been outlined, the interventions are often described interchangeably in the global context, even if that masks some of the heterogeneity in provision.

#### 2.2.5 Supported housing

Supported housing is an umbrella term for various accommodation‐based approaches and therefore an extremely complex intervention type. When providers describe their approach as supported housing, the intervention will typically combine housing with additional supportive services as an integrated package. The housing offered can be permanent or temporary; nonabstinent contingent or abstinent‐contingent; staffed group homes, community based or in a private unit; and the subsidies towards rent also vary. Supportive services will be offered directly to the individual or through referrals to the relevant body. Supportive services might include those to help with mental health issues, substance misuse, those interventions which increase access to health services, support to continue education or find employment, help with accessing benefits, or those services which focus on social aspects of the individual's life such as positive interactions with society, or community engagement. Due to the inconsistencies in the approach which “supported housing” takes, and the wide range of housing and support offered through supported housing interventions, it is incredibly difficult to group supported housing as a homogenous set of interventions for which to compare effectiveness to other groups of accommodation‐based approaches.

#### 2.2.6 Conclusion

In homelessness literature, there is difficulty both in defining homelessness and the interventions which seek to benefit individuals (FEANTSA, 2017). Suttor argues that while it may be advantageous to create interventions tailored to an individual's unique needs, there is a need to classify approaches (Suttor, 2016). Indeed, most commentators acknowledge the challenges of lack of clear definition of the many terminologies used to describe accommodation‐based interventions. One example of this is highlighted in a study which identified 307 unique terms across 400 articles on supported accommodation (Gustafsson et al., 2009). Additionally, the Housing First model initially seems like an approach where categorisation is straightforward, however, there exists significant inconsistencies regarding implementation. Various researchers observe that this may be due to the way the Housing First model has deviated from the original “Pathways to Housing” intervention (Tsemberis & Eisenberg, 2000) due in part to additional services and support (Johnson et al., 2012; Phillips et al., 2011).

### 2.3 A new typology

Due to the diverse nature of the literature on accommodation‐based approaches, the way in which the approaches are implemented in practice, and the disordered descriptions of the categories, it became apparent that the review team must create meaningful categorisations to allow functional and useful comparison between the various intervention types. The importance of these categorisations cannot be understated, as it provides a comparative international framework from which policy makers and funders can work to understand the effectiveness of different accommodation‐based interventions.

One such typology already exists and is based on an international evidence review of 533 interventions for rough sleepers (Mackie et al., 2017). This review was led by one of the current review authors and identified characteristics of various types of temporary accommodation, namely shelters and hostels. The review team adapted this typology to inform the development of categories for the accommodation‐based interventions. This process was undertaken alongside Lipton and colleagues' (Lipton et al., 2000) descriptive categorisation of low, moderate, or high intensity housing which is based on the degree of structure and level of independence offered to their 2937 study participants. A further category (housing only) was added to allow for interventions which focused on providing accommodation for an extended period without further support or services offered. It was deemed to be more than just meeting the basic needs of the individual, but not intense enough to meet the criteria of the moderate category, as individuals were not receiving any additional services or help.

To develop the typology further, we used an iterative decision model. First, the review team selected a random sample of five accommodation‐based interventions included in the Evidence and Gap Map (EGM) of homelessness interventions (White et al., 2020), upon which this review is based. Second, two review team members independently coded the characteristics, hypotheses and concepts related to each intervention and compared notes. This independent analysis of the sampled papers ensured both objectivity and consistency in this step of the process and allowed the reviewers to investigate substantial amounts of data without bias or a predetermined hypothesis. Third, emerging themes were collated, and reviewers communicated to better understand the patterns which appeared through the sampled studies. Finally, through this iterative process we conclude that the most suitable way to create meaningful categorisations would be based around the intensity (defined as the level of the support offered).

Furthermore, interventions varied on the conditions the user was required to abide by. These conditions include needing to be sober from alcohol and/or drugs, abstain from criminal activity or to gain employment after a certain amount of time. To accurately incorporate these conditions into the categories, it must be stated whether the intervention required such a behavioural condition (conditional) or whether there were no behavioural conditions imposed (unconditional). The typology is described below and presented in Table 1.

1. **Basic/conditional**
  Interventions that meet the user's basic human needs only. This would be the provision of a bed and other basic subsistence such as food. There are no named additional services or support offered to the user. This type of intervention focuses more on the short‐term benefit to the user. The accommodation or support offered will require further conditions from the user upon admission such as sobriety or punctuality. An example of this intervention type would be if users were given one night in a shelter with a meal on the condition that they arrive by 11 pm.
2. **Basic/unconditional**
  Interventions which offer only minimal sleeping facilities to the user without additional services or support. Unlike the type of intervention described above, there are no behavioural expectations placed on the individual. An example of this would be if users were provided access to a shelter without exception.
3. **Housing only/conditional**
  The users are provided a form of accommodation for an extended period, with conditions, but without additional support or services. An example of this is shown in Siegel et al. (2006): one of the interventions described provides participants with housing where they are assisted with rent. Tenants were responsible for their own meals and utility expenses. An example of the behavioural expectations imposed on users receiving this type of intervention may be that they must enter paid employment within six months.
4. **Housing only/unconditional**
  Provision of housing for an extended period but without further support and services offered to the user. The participant is not required or obligated to meet any behavioural expectation to access the housing.
5. **Moderate support/conditional**
  Moderate levels of support and/or services are provided in addition to housing. The level of support and type of service offered will remain general and aimed towards a group of people experiencing homelessness, and not specific to individual personal needs. This housing, coupled with general support and services, will be offered on the condition that an individual meets a behavioural expectation. For example, Sosin et al. (1996) housing intervention a moderately intensive drug case management intervention was offered alongside the housing. To take part, participants had to sign a contract agreeing to abstain from drugs and or alcohol.
6. **Moderate support/unconditional**
  Interventions in this category are the same as the above category except there will be no behavioural expectation placed on the user for accessing the intervention. For example, Lim et al. (2017) focused on accessing cheaper housing and provided additional services to prevent youth from becoming homeless. The participants were encouraged to attend but it was not strictly enforced and there were no conditions placed upon the individuals to partake in the intervention.
7. **High support/conditional**
  These interventions provide housing and actively work to improve user's long‐term outcomes. The intervention provides assertive, individualised services and interventions for users. The intervention can involve improving housing stability, health, and employment, among other specific needs. The accommodation or support offered may place a behavioural expectation upon the person upon admission to the intervention. For example, participants in Schumacher et al. (2003) were provided housing alongside intensive treatment and other services. All participants were routinely tested for drugs and alcohol and were not allowed to continue with the intervention until they were deemed sober.
8. **High support/unconditional**
  Interventions in this category are the same as the above category except there is no behavioural expectation placed on the user. For example, Levitt et al. (2013) intervention included providing housing, meals and on‐site care services. On‐site case managers would consistently work with each individual participant on their substance use and life goals. The participant did not need to be sober to partake in the intervention.
9. **No intervention**

Interventions in this category would be those that do not actively work to improve the lives of the users. The user is not offered a bed/food or any additional support. An example of this is demonstrated in Sosin et al. (1996) article. The control group used in this experiment received no additional aid. Those in the control group received some minimal information on where they could receive help in the form of abuse agencies or welfare offices but were not offered any additional help or services.

**Table 1:** Typology: summary of categories

	Type of accommodation	Support	Conditionality
Basic/conditional	Interventions that meet the user's basic human needs only, for example, providing bed and other basic subsistence such as food.	There are no named additional services or support offered to the user. This type of intervention focuses more on the short‐term benefit to the user	Conditions such as sobriety or punctuality apply
Basic/unconditional	Interventions that meet the user's basic human needs only, for example, providing bed and other basic subsistence such as food.	There are no named additional services or support offered to the user. This type of intervention focuses more on the short‐term benefit to the user	Accommodation is not conditional on adherence to rules such as sobriety or punctuality
Housing only/conditional	Accommodation provided for an extended period	Without additional support or services	Behavioural expectations are imposed on users, for example, they must enter paid employment within six months
Housing only/unconditional	Accommodation provided for an extended period	Without additional support or services	The participant is not required or obligated to meet any behavioural expectation to retain their housing
Moderate support/conditional	Accommodation provided for an extended period	The level of support and type of service offered will remain general and aimed towards a group of homeless individuals, and not specific to individual personal needs	Expectations on behaviour in place for example signing a contract agreeing to abstain from drugs and or alcohol
Moderate support/unconditional	Accommodation provided for an extended period	The level of support and type of service offered will remain general and aimed towards a group of homeless individuals, and not specific to individual personal needs	Accommodation not conditional on engagement (though engagement may be encouraged)
High support/conditional	Accommodation provided for an extended period	Assertive, individualised services and interventions for users. They often focus specifically on the personal needs of the user	Expectations such as abstinence from alcohol and drugs in place
High support/unconditional	Accommodation provided for an extended period	Assertive, individualised services and interventions for users. They often focus specifically on the personal needs of the user	No behavioural expectation such as sobriety placed on the user
No intervention (control groups)	None provided	None provided except basic information about other services	Not applicable

### 2.4 How the intervention might work

The distinctive component shared by all accommodation‐based interventions is that accommodation will be provided to individuals (even if only for the short‐term). Some interventions may also provide services alongside the accommodation and support they require to continue life independently without the risk of future homelessness.

### 2.5 Why it is important to do this review

The aim of this systematic review is to establish the effectiveness of accommodation‐based approaches though a robust and rigorous synthesis of the available literature. The typology described above provides a framework that potentially allows us to rank the effectiveness of interventions according to the different categories. However, this is only possible if there are sufficient eligible studies in each category.

#### 2.5.1 Previous reviews

This systematic review is based on evidence already identified in two existing EGMs commissioned by the Centre for Homelessness Impact (CHI) and built by White et al. (2020). The EGMs present studies on the effectiveness and implementation of interventions aimed at people experiencing, or at risk of experiencing, homelessness.

The EGMs identified various systematic reviews which assess the effectiveness of interventions like Housing First (Beaudoin, 2016; Woodhall‐Melnik & Dunn, 2016) and supported housing (Burgoyne, 2013; Nelson et al., 2007; Richter & Hoffmann, 2017), and interventions which were conducted in hostel and shelter settings (Haskett et al., 2016; Hudson et al., 2016). However, an analysis comparing the relative effectiveness of different categories of accommodation‐based interventions for people who are homeless (for example, using network meta‐analysis) does not exist. Various systematic reviews which synthesise accommodation‐based interventions more generally, differ from the proposed review in several ways:

##### Differences in population

Bassuk, DeCandia, Tsertsvadze, and Richard (Bassuk et al., 2014) systematically reviewed and narratively reported the findings of six studies which looked at the effectiveness of housing interventions and housing combined with additional services. The interventions included Housing First, rapid rehousing, vouchers, subsidies, emergency shelter, transitional housing, and permanent supportive housing. However, authors limited the population to American families who were experiencing homelessness and so any final conclusions on the efficacy of accommodation‐based interventions on the wider population of individuals experiencing homelessness are impossible to reach.

##### Differences in outcomes of interest

Fitzpatrick‐Lewis and colleagues (Fitzpatrick‐Lewis et al., 2011) conducted a rapid systematic review on the effectiveness of interventions to improve the health and housing status of individuals experiencing homeless which located 84 relevant studies. Only those studies published between January 2004 and December 2009 were included in this review and so the current review is more up to date and broader in scope. Additionally, the primary purpose of the review was to identify literature which improved health outcomes for those experiencing homelessness and so other important outcomes were not included.

Mathew and colleagues (Mathew et al., 2018) conducted a Campbell Collaboration systematic review which looks at how various interventions impact the physical and mental health of people who are homeless alongside other social outcomes. One objective listed in the title registration form is similar to the scope of the current review. Authors assessed “What are the effects of housing models (i.e., Housing First) on the health outcomes of homeless and vulnerably housed adults compared to usual or no housing?” However, the current review has a wider scope by including additional outcomes across a wider population.

A second Campbell Collaboration systematic review (Munthe‐Kaas et al., 2018) assessed the effectiveness of both housing and case management programmes for people experiencing, or at risk of experiencing homelessness. The main outcomes of interest to the authors were reduction in homelessness and housing stability. Authors searched the literature until January 2016 and uncovered 43 RCTs meeting the predetermined inclusion criteria. Authors did not include qualitative research or extract data related to the cost of the interventions, which are variables of interest to this proposed review.

##### Differences in analytic methods

A recent review by the What Works Centre for Wellbeing (Chambers et al., 2018) included 90 studies which included clusters of Housing First (*n* = 47), supported housing (*n *= 12), recovery housing (*n* = 10), housing interventions for ex‐prisoners (*n* = 7), housing interventions for vulnerable youth (*n* = 3) and “other” complex interventions targeted at those with poor mental health (*n* = 11). Authors presented a comprehensive search strategy of both commercial and grey literature, however, due to resource constraints were unable to conduct independent screening of the potential studies and therefore risk selection bias in the review. Additionally, only studies published after 2005 were included in this review and so the current review is much broader in scope. Finally, the authors' objective was to create a conceptual pathway and evidence map between housing and wellbeing and so the results were not meta‐analysed but described narratively instead.

##### Inclusion of qualitative studies

Finally, this review also includes qualitative data, to complement the quantitative results on effectiveness, by highlighting important implementation and process issues related to the delivery and uptake of accommodation‐based services. The qualitative studies included in this element of the report are drawn from CHI's implementation and process EGM and described in more detail below.

## 3 OBJECTIVES

1. What is the effect of accommodation‐based interventions on outcomes including housing stability, health, employment, crime, and wellbeing, for individuals experiencing or at risk of experiencing homelessness?
2. Which type of intervention is most/least effective compared to other interventions and compared to business as usual (passive control)?
3. Who do accommodation‐based interventions work best for?
  a. Young people or older adults?
  b. Individuals with high or low complex needs?
  c. Families or single individuals?
4. Does the geographical spread of housing (scattered site or conglomerate/congregate) affect the outcomes experienced by individuals experiencing or at risk of experiencing homelessness?
5. What implementation and process factors impact intervention delivery?

## 4 METHODS

### 4.1 Criteria for considering studies for this review

#### 4.1.1 Types of studies

The systematic review and network meta‐analysis was prospectively registered with the Campbell Collaboration to improve quality of the review, promote transparency and replicability, and avoid duplication of effort. The protocol was published in September 2020 (Keenan et al., 2020), and can be accessed through the Campbell Collaboration library.

We included all study designs where a comparison group was used. This included randomised controlled trials (RCTs), quasi‐experimental designs, matched comparisons and other study designs that attempt to isolate the impact of the intervention on homelessness using appropriate statistical modelling techniques.

As RCTs are accepted as more rigorous than nonrandomised studies, the potential impact of a nonrandomised study design on effect sizes was explored as part of the analysis of heterogeneity.

Studies were eligible for inclusion in the review if they included a comparison condition, for example:

- **No treatment**.
- **Treatment as usual** where people receive their normal level of support or intervention.
- **Waiting list** where individuals or groups are randomly assigned to receive the intervention at a later date.
- **Attention control**, where participants receive some contact from researchers but both participants and researchers are aware that this is not an active intervention.
- **Alternative treatment**, an active accommodation‐based approach used to compare treatments.
- **Placebo** where participants perceive that they are receiving an active intervention, but the researchers regard the treatment as inactive.

Studies with no control or comparison group, unmatched controls or national comparisons with no attempt to control for relevant covariates were not included. Case studies, opinion pieces or editorials were also not included.

#### 4.1.2 Types of participants

This systematic review focused on all individuals currently experiencing, or at risk of experiencing homelessness irrespective of age or gender, in high‐income countries. Homelessness is defined as those individuals who are sleeping “rough” (sometimes defined as street homeless), those in temporary accommodation (such as shelters and hostels), those in insecure accommodation (such as those facing eviction or in abusive or unsafe environments), and those in inadequate accommodation (environments which are unhygienic and/or overcrowded).

#### 4.1.3 Types of interventions

Interventions included those based on the typology outlined above and were classified according to the nature and characteristics of the intervention rather than the descriptor provided by the study author(s).

The control or comparison condition can include no services/intervention, services as usual, waitlist control, attention control, placebo or an alternative accommodation‐based intervention (see Section 4.1.1 for more detail).

#### 4.1.4 Types of outcome measures

##### Primary outcomes

This review primarily addresses how interventions can reduce homelessness and increase housing stability for those individuals experiencing, or at risk of experiencing, homelessness.

##### Secondary outcomes

Secondary outcomes include:

- Access to mainstream healthcare
- Crime and justice
- Employment and income
- Capabilities and wellbeing
- Cost of intervention.

These outcomes reflect the domains used in the EGM (White et al., 2020), with the addition of cost.

###### Types of settings

Settings where these accommodation‐based interventions take place were varied and included hostels, shelters, and community housing.

### 4.2 Search methods for identification of studies

This systematic review is based only on the evidence already identified in two existing EGMs commissioned by the Centre for Homelessness Impact (CHI) and built by White et al. (2020). The EGMs include studies on the effectiveness and implementation of interventions aimed at people experiencing, or at risk of experiencing, homelessness in high income countries.

#### 4.2.1 Electronic searches

The maps used a comprehensive three stage search and mapping process. Stage one was to map the included studies in an existing Campbell review on homelessness (Munthe‐Kaas et al., 2018), stage two was a comprehensive search of 17 academic databases, three EGM databases, and eight systematic review databases for primary studies and systematic reviews. Finally stage three included web searches for grey literature, scanning reference lists of included studies and consultation with experts to identify additional literature. Sample search terms can be found in the protocol (White et al., 2020).

#### 4.2.2 Searching other resources

We did not undertake any additional searching. However, while contacting authors for additional information, authors of the Chez Soi trial (Goering et al., 2011) provided additional reports of identified studies. The inclusion of these reports provided extra data necessary for conducting analysis and ROB assessments

### 4.3 Data collection and analysis

**Figure 1:**
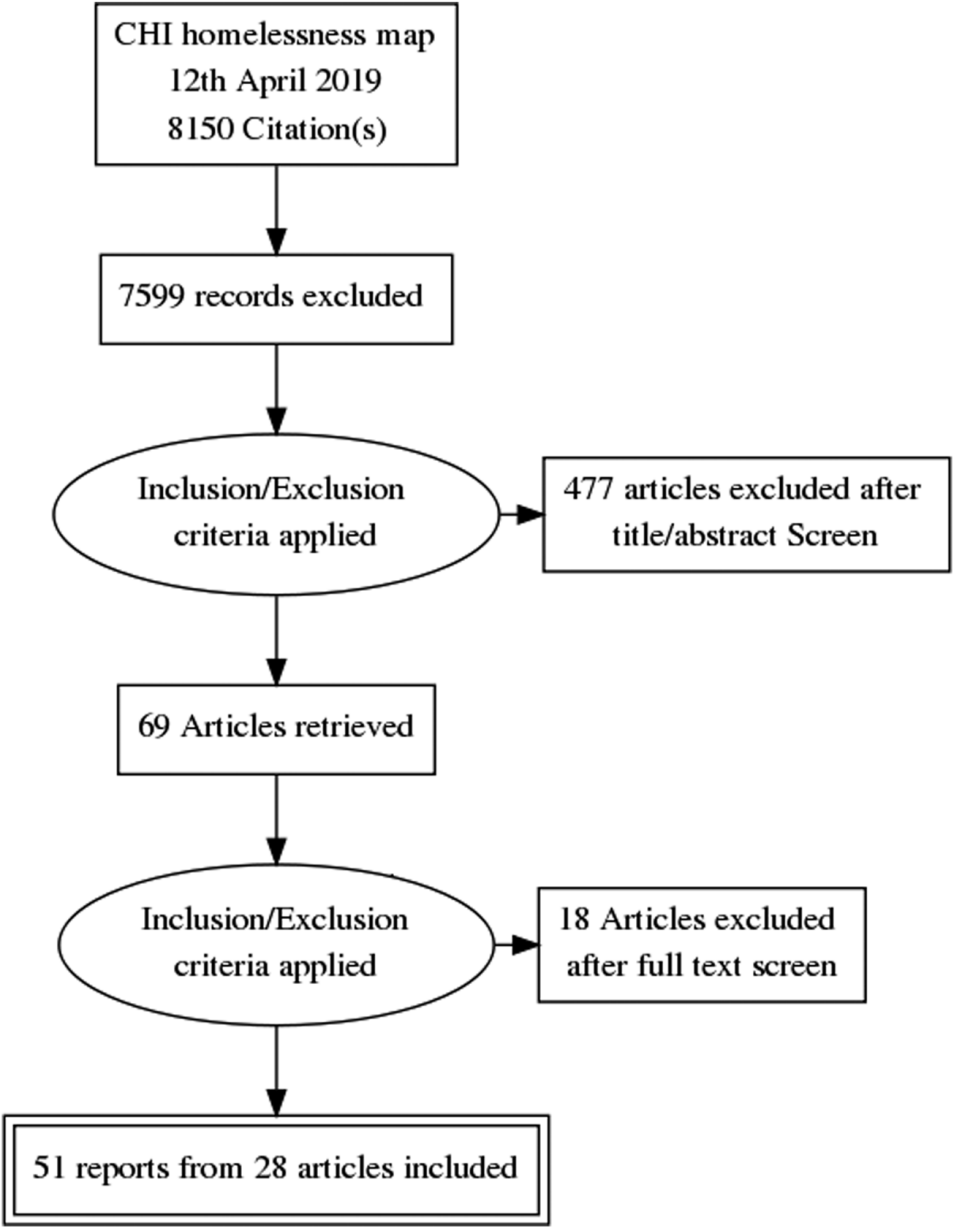
PRISMA flow diagram

To identify studies from the map that were eligible for inclusion in this review, two reviewers independently screened the title and abstract of all documents in the effectiveness map using EPPI Reviewer 4 software. The full text of studies that met or appeared to meet the inclusion criteria were then screened independently by two reviewers. Any disagreements were resolved in discussion with a third reviewer until a consensus was reached. The same process was applied to screening documents included in the process evaluation maps to identify studies eligible for inclusion in the qualitative synthesis. The flow of studies through the screening process are documented in a PRISMA flow chart (Figure 1).

#### 4.3.1 Description of methods used in primary research

Interventions included RCTs and quasi‐experimental studies measuring the effectiveness of accommodation‐based approaches against either a control group or through head‐to‐head comparisons with an alternative (accommodation‐based) treatment.

#### 4.3.2 Criteria for determination of independent findings

Often, authors reported data on the same participants across more than one outcome, this leads to multiple dependent effect sizes within each single study. The meta‐analysis therefore used robust variance estimation to adjust for effect size dependency (Hedges et al., 2010). The correction for small samples (Tipton & Pustejovsky, 2015) was implemented when necessary. Finally, in cases where study authors separate participants into subgroups relating to age, comorbid diagnosis, or gender and it is inappropriate to pool their data, these participants remained independent of each other and were treated as separate studies which each provide unique information.

#### 4.3.3 Selection of studies

To identify studies from the map that were eligible for inclusion in this review, two reviewers independently screened the title and abstract of all documents in the effectiveness map using EPPI Reviewer 4 software. The full text of studies that met or appeared to meet the inclusion criteria were then screened independently by two reviewers. Any disagreements were resolved in discussion with a third reviewer until a consensus was reached. The same process was applied to screening documents included in the process evaluation maps to identify studies eligible for inclusion in the qualitative synthesis. The flow of studies through the screening process are documented in a PRISMA flow chart Figure 1.

#### 4.3.4 Data extraction and management

Once eligible studies were identified, we undertook dual data extraction, where two authors completed data extraction and ROB assessments independently for each study. Coding was carried out by trained researchers. Any discrepancies in screening or coding were discussed with senior authors until a consensus was reached.

##### Details of study coding categories

A data extraction tool was designed by the authors and piloted by trained research assistants using EPPI Reviewer (Appendix A). At a minimum, we extracted the following data: publication details, intervention details including setting, implementation, delivery personnel, descriptions of the outcomes of interest including instruments used to measure, design and type of trial, sample size of treatment and control groups, data required to calculate Hedge's *g* effect sizes, quality assessment. We extracted more detailed information on the interventions such as: duration and intensity of the programme, timing of delivery, key programme components (as described by study authors), theory of change.

Alongside extracting data on programme components, descriptive information for each of the studies was extracted and coded to allow for sensitivity and subgroup analysis. This included information regarding:

- Setting in which the intervention is delivered
- Study characteristics in relation to design, sample sizes, measures and attrition rates, who funded the study and potential conflicts of interest.
- Demographic variables relating to the participants including age, complexity of needs, dependent children, and other relevant population characteristics.

Quantitative data were extracted at immediate post‐test to allow for calculation of effect sizes (such as mean change scores and standard error or pre‐ and post‐ means and *SD*s or binary 2 × 2 tables). Data were then extracted for the intervention and control groups on the relevant outcomes measured, in order to assess the intervention effects.

#### 4.3.5 Assessment of ROB in included studies

Assessment of methodological quality and potential for bias was conducted using the second version of the Cochrane Risk of Bias tool for RCTs (Higgins et al., 2019). The methodological quality of nonrandomised studies was coded using the ROBINS‐ I tool (Sterne et al., 2016).

#### 4.3.6 Measures of treatment effect

##### Statistical procedures and conventions

Most outcomes reported were based on continuous variables and so the main effect size metric that was used for the purposes of the meta‐analyses was the standardised mean difference, with its 95% confidence interval. Within this, Hedges' g was used to correct for any small sample bias. Where other effect sizes were reported, such as Cohen's *d* or risk ratios (for dichotomous outcomes) these were converted to Hedges' *g* for the purposes of the meta‐analysis using formulae provided in the Cochrane Handbook (Higgins et al., 2019).

Most outcomes were calculated using the David‐Wilson Calculator (Wilson, 2019), utilising formulae to find the effect size of several continuous data, including means and *SD*s. Hozo's Formula (Hozo et al., 2005) was also used to help calculate effect sizes when Interquartile range and Median data were provided.

#### 4.3.7 Unit of analysis issues

The analyses presented utilised a random effects model (REM), estimating the variance component with restricted (or residual, or reduced) maximum likelihood (REML). The REM was chosen as the statistical model as it accepts two main differences among primary studies, the first is within study variance, and the second is between study variance. This between study variance, or heterogeneity, can reflect important differences in populations, settings, or progression of time (Borenstein et al., 2009). To allow for estimation of the variance components, the Satterthwaite approximation was used to account for two different sample variances where only estimates of the variance are known. The analysis is useful to calculate an approximation to the effective degrees of freedom (Satterthwaite, 1946).

#### 4.3.8 Dealing with missing data

If study reports did not contain sufficient data to allow calculation of effect size estimates, authors were contacted to obtain necessary summary data, such as means and *SD*s or standard errors. If no information were forthcoming, the study could not be included in meta‐analysis and was instead included in a narrative synthesis.

#### 4.3.9 Assessment of heterogeneity

The meta‐analysis included the overall mean and prediction interval for all primary outcomes in the analysis to examine the distribution of effect sizes. The analysis was conducted in two phases: (a) the use of meta‐regression to examine heterogeneity across studies, and (b) a network meta‐analysis (NMA) to address the relative effects of the included interventions.

#### 4.3.10 Assessment of reporting biases

A problem which threatens the conclusions made by every meta‐analysis is the potential for publication bias. This threat arises from the decreased likelihood of studies which have negative or insignificant results to be published, and therefore the studies available to the researcher will not be representative of all the studies conducted on the topic of interest. Using the metafor package in R (Viechtbauer, 2010), the samples were visually investigated for publication bias using a funnel plot.

#### 4.3.11 Data synthesis

When conducting meta‐analysis on the effectiveness of accommodation‐based interventions, we were attentive to whether different types of accommodation‐based intervention (as defined by our typology) are more or less effective for individuals experiencing homelessness. Few of the included trials compared the effects of two interventions directly (*n* = 11) and so direct comparisons between some accommodation‐based interventions do not exist, however the majority of interventions were tested against equivalent control groups. Thus, through NMA, it is possible to calculate the indirect effects of comparative accommodation‐based interventions and produce this as a “network” of comparisons. These analyses were completed via a frequentist model using R package, *netmeta*, and are reported below.

#### 4.3.12 Subgroup analysis and investigation of heterogeneity

We conducted moderator analyses that test whether specific characteristics of the studies or the interventions can explain some of the heterogeneity in results. It is important to understand that moderator analyses are exploratory and should never be implemented to test hypotheses. Even if the meta‐analysis contains only studies with specific methodologies (RCTs and quasi‐experiments), the studies involved in these moderator analyses have not been randomised, they are observational in nature and at a higher ROB. Additionally, these type of analyses generally have lower power due to missing data in the primary research, there is an increased risk of presenting incorrect results which appear simply through chance (false positive conclusion), and potential for various biases (Borenstein et al., 2009; Higgins et al., 2019). Although these analyses are a common inclusion to many meta‐analyses as they are useful for developing ideas and exploring heterogeneity, moderator analysis have low statistical power and should always be interpreted with caution (Borenstein et al., 2009).

We used the R programmes *metafor* (Viechtbauer, 2010) for analyses, *netmeta* for NMA (Rücker et al., 2015), and *clubSandwich* (Pustejovsky, 2017) to adjust the standard errors of the model for dependencies. The intended moderators for subgroup analyses included: participant age, complexity of need, whether the intervention was focused on families or individuals, geographical spread of housing (scattered site or conglomerate), study design, and ROB.

##### Treatment of qualitative research

The qualitative research that was included in this review is based upon existing evidence collated through the second implementation and process EGM constructed by White et al. (2018). The EGM includes 292 qualitative process evaluations on the implementation issues associated with interventions designed to target homelessness. These are not the same studies that are included in the effectiveness EGM or included in the meta‐analyses reported below. These qualitative reports were downloaded from EPPI reviewer on 10th May 2019 and screened for relevance to the current review.

The EGM categorises included studies into broad categories of barriers and facilitators to the implementation of interventions. These categories were developed by the original authors of the EGM using an iterative process and were initially based on the implementation science framework (Aarons et al., 2011). The categories were independently piloted against a small number of process evaluations and agreement was reached by researchers in the Campbell Collaboration, Campbell UK and Ireland, and Heriot‐Watt University. The five broad categories are contextual factors, policy makers/funders, programme managers/implementing agency, staff/case workers, and recipients. The review team recognise that in the majority of accommodation‐based interventions, more than one of the agreed categories could act as a factor that impacts positively or negatively on the effectiveness of the intervention, or both in some cases. This potential overlap reflects the complexity of the implementation of the interventions and the multifaceted evaluation tools needed within this review. For this reason, the review team decided to focus on factors that influence intervention effectiveness in order to formulate a coherent Synthesis Framework.

We included process evaluations and other relevant qualitative studies that provide data that enables a deeper understanding of why the accommodation‐based programmes included in the quantitative synthesis do (or do not) work as intended, for whom and under what circumstances. We conducted a “Best Fit” Framework synthesis in order to have a highly structured approach to organising and analysing data, which can prove difficult to do with qualitative data. This method is largely informed by background material and team discussions to extract and synthesise findings. This is particularly useful given the mixed methods approach, as the quantitative and qualitative data can work in tandem to give the clearest results possible.

#### 4.3.13 Sensitivity analysis

Every meta‐analysis includes decisions made by the researchers which may affect the findings and inferences which can be drawn from the conclusions. In this meta‐analysis, two sensitivity analyses were employed to explore the robustness of the overall results by removing certain study characteristics which may cause influence on the outcome of the analysis.These included study design and ROB.

#### 4.3.14 Summary of findings and assessment of the certainty of the evidence

The quality of these mixed methods studies was assessed using a tool developed by White and Keenan (Appendix A, Part 7). The tool is similar to the fidelity assessment used by Stergiopoulos et al. (2016) and aims to provide an accurate account of the eligible qualitative studies. The tool considers methodology, recruitment and sampling, bias, ethics, analysis and findings. We also describe the characteristics of included qualitative studies in terms of what qualitative methods have been used to capture this rich data, the number of interviews/focus groups/observations that have taken place, who participated and the nature of qualitative data collection.

## 5 RESULTS

### 5.1 Description of studies

We identified 223 unique studies across 551 articles from the effectiveness map on 12th April 2019. Of these 551 articles, we deemed 69 to meet eligible inclusion criteria following title and abstract screening. Full text screening led to the exclusion of a further 18. More details can be found in the PRISMA Flow Diagram (Figure 1). In total, 28 eligible studies reported in 51 accommodation intervention papers were identified and included in this review:

**Study ID: Appel 2012**

- Housing first for severely mentally Ill homeless methadone patients (Appel et al., 2012).
**Study ID: Brown 2016**
- Housing first as an effective model for community stabilisation among vulnerable individuals with chronic and nonchronic homelessness histories (Brown et al., 2016).
**Study ID: Buchanan 2006**
- The effects of respite care for homeless patients: A cohort study (Buchanan et al., 2006).
**Study ID: Buchanan 2009**
- The health impact of supportive housing for HIV‐positive homeless patients: A RCT (Buchanan et al., 2009).
**Study ID: Cheng 2007**
- Impact of supported housing on clinical outcomes analysis of a randomised trial using multiple imputation technique (Cheng et al., 2007).
**Study ID: Gilmer 2010**
- Effect of full‐service partnerships on homelessness, use and costs of mental health services, and quality of life among adults with serious mental illness (Gilmer et al., 2010).
**Study ID: Goering 2011 (Chez Soi)**
- The at Home/Chez Soi trial protocol: A pragmatic, multi‐site, RCT of housing first in five Canadian cities (Goering et al., 2011).
- Effect of housing first on suicidal behaviour: A randomised controlled trial of homeless adults with mental disorders (Aquin et al., 2017).
- Housing First for people with severe mental illness who are homeless: A review of the research and findings from the At Home‐Chez soi demonstration project (Aubry et al., 2015).
- At Home/Chez Soi interim report (Goering, 2012).
- The impact of a Housing First RCT on substance use problems among homeless individuals with mental illness (Kirst et al., 2015).
- “Housing First” for homeless youth with mental illness (Kozloff et al., 2016).
- At Home/Chez Soi randomised trial: How did a Housing First intervention improve health and social outcomes among homeless adults with mental illness in Toronto? Two‐year outcomes from a randomised trial (O'Campo et al., 2016).
- Housing first improves subjective quality of life among homeless adults with mental illness: 12‐month findings from a RCT in Vancouver, British Columbia (Patterson et al., 2013).
- Effects of housing first on employment and income of homeless individuals: Results of a Randomised Trial (Poremski et al., 2016).
- Housing First improves adherence to antipsychotic medication among formerly homeless adults with schizophrenia: Results of a RCT (Rezansoff et al., 2017).
- Emergency department utilisation among formerly homeless adults with mental disorders after one year of Housing First interventions: a RCT (Russolillo et al., 2014).
- Effect of scattered‐site housing using rent supplements and intensive case management on housing stability among homeless adults with mental illness (Stergiopoulos et al., 2015).

**Study ID: Goldfinger 1999**

- Housing placement and subsequent days homeless among formerly homeless adults with mental illness (Goldfinger et al., 1999).
**Study ID: Gulcur 2003 (Pathways to Housing)**
- Housing, hospitalisation, and cost outcomes for homeless individuals with psychiatric disabilities participating in continuum of care and Housing First programmes (Gulcur et al., 2003).
- Decreasing psychiatric symptoms by increasing choice in services for adults with histories of homelessness (Greenwood et al., 2005).
- Housing first, consumer choice, and harm reduction for homeless individuals with a dual diagnosis (Tsemberis et al., 2004).
- Consumer preference programmes for individuals who are homeless and have psychiatric disabilities: A drop‐in centre and a supported housing programme (Tsemberis et al., 2003).

**Study ID: Howard 2011**

- Effectiveness and cost‐effectiveness of admissions to women's crisis houses compared with traditional psychiatric wards: Pilot patient‐preference RCT (Howard et al., 2011).
**Study ID: Hwang 2011**
- Health status, quality of life, residential stability, substance use, and health care utilisation among adults applying to a supportive housing programme (Hwang et al., 2011).
**Study ID: Kertesz 2007**
- Long‐term housing and work outcomes among treated cocaine‐dependent homeless persons (Kertesz et al., 2007).
- To house or not to house: The effects of providing housing to homeless substance abusers in treatment (Milby et al., 2005).
- Costs and effectiveness of treating homeless persons with cocaine addiction with alternative contingency management strategies (Mennemeyer et al., 2017).

**Study ID: Larimer 2009**

- Health care and public service use and costs before and after provision of housing for chronically homeless persons with severe alcohol problems (Larimer et al., 2009).
**Study ID: Levitt 2013**
- Randomised trial of intensive housing placement and community transition services for episodic and recidivist homeless families (Levitt et al., 2013).
**Study ID: Lim 2017**
- Impact of a supportive housing program on housing stability and sexually transmitted infections among young adults in New York City who were aging out of foster care (Lim et al., 2017).
**Study ID: Li m 2018**
- Impact of a New York City supportive housing programme on Medicaid expenditure patterns among people with serious mental illness and chronic homelessness (Lim et al., 2018).
**Study ID: Lipton 2000**
- Tenure in supportive housing for homeless persons with severe mental illness (Lipton et al., 2000).
**Study ID: McHugo 2004**
- A randomized controlled trial of integrated versus parallel housing services for homeless adults with severe mental illness (McHugo et al., 2004).
**Study ID: Milby 1996**
- Sufficient conditions for effective treatment of substance abusing homeless persons (Milby et al., 1996).
- Costs and effectiveness of treating homeless persons with cocaine addiction with alternative contingency management strategies (Mennemeyer et al., 2017).

**Study ID: Milby 2000**

- Initiating abstinence in cocaine abusing dually diagnosed homeless persons (Milby et al., 2000).
- Costs and effectiveness of treating homeless persons with cocaine addiction with alternative contingency management strategies (Mennemeyer et al., 2017).

**Study ID: Milby 2008**

- Toward cost‐effective initial care for substance‐abusing homeless (Milby et al., 2008).
- Effects of sustained abstinence among treated substance‐abusing homeless persons on housing and employment (Milby et al., 2010).
- Costs and effectiveness of treating homeless persons with cocaine addiction with alternative contingency management strategies (Mennemeyer et al., 2017).

**Study ID: O'Connell 2012**

- Differential impact of supported housing on selected subgroups of homeless veterans with substance abuse histories (O'Connell et al., 2012).
**Study ID: Sadowski 2009**
- Effect of a housing and case management program on emergency department visits and hospitalizations among chronically Ill homeless adults a randomized trial (Sadowski et al., 2009).
- Comparative cost analysis of housing and case management program for chronically Ill homeless adults compared to usual care (Basu et al., 2012).

**Study ID: Shern 1997 (Choices)**

- Housing outcomes for homeless adults with mental illness: Results from the second‐round McKinney Program (Shern et al., 1997).
- Serving street‐dwelling individuals with psychiatric disabilities: Outcomes of a psychiatric rehabilitation clinical trial (Shern et al., 2000).
- Consumer preference programmes for individuals who are homeless and have psychiatric disabilities: a drop‐in centre and a supported housing programme (Tsemberis et al., 2003).

**Study ID: Siegel 2006**

- Tenant outcomes in supported housing and community residences in New York City (Siegel et al., 2006).
**Study ID: Sos in 1996**
- Paths and impacts in the progressive independence model: A homelessness and substance abuse intervention in Chicago (Sosin et al., 1996).
**Study ID: Srebnik 2013 (Begin at Home)**
- A pilot study of the impact of Housing First‐supported housing for intensive users of medical hospitalisation and sobering services (Srebnik et al., 2013)
**Study ID: Stefancic 2007**
- Housing First for long‐term shelter dwellers with psychiatric disabilities in a suburban county: a four‐year study of housing access and retention (Stefancic & Tsemberis, 2007).

#### 5.1.1 Results of the search

The flow of studies through the screening process are documented in a PRISMA flow chart (Figure 1).

#### 5.1.2 Included studies

There was a total of 13,128 people included in the review, across 28 studies. Most of the included studies were carried out in the United States of America (25/28), with other locations including Canada (Goering et al., 2011; Hwang et al., 2011) and the UK (Howard et al., 2011). The location of the studies was largely urbanised, with 26/28 of the studies conducted in cities, with one study not specifying its location (O'Connell et al., 2012), and the other focusing on suburban homelessness (Stefancic & Tsemberis, 2007).

Twenty‐seven of the 28 studies were published in journal articles. Sixteen studies were RCTs (57%) and 12 were nonrandomised (quasi‐experimental) designs (43%).

The mean age of all participants was 36.7 years. Most participants were men, on average samples were 71.3% men (ranging from 47.5% to 100% men). In all but two studies the participants had complex needs with poor mental health and substance use issues the main needs identified, and in some studies, the population that participants were drawn from was specifically targeted because of chronic homelessness and multiple complex needs.

The two main sources of funding were research council funding and grants or loans from trusts and charities. Three studies did not specify their source of funding (Brown et al., 2016; Siegel et al., 2006; Stefancic & Tsemberis, 2007). More details on the characteristics of the included studies can be found in Table 2.

**Table 2:** Characteristics of Included studies

Study title	Name of intervention(s)	Complexity of needs	Sample size	Age	Sex	Design	Typology
Appel (2012)	Keeping “Home project”: Housing First approach	Poor Mental Health and Substance abuse issues	61	Mean: Intervention, 45.9 (range 26–63); Control, 39.7 (no range mentioned)	Male: Intervention, 26 (80.8%); Control, 19 (63.3%). Female: Intervention, 5 (19.2%); Control, 11 (36.7%)	Non‐RCT	High unconditional
Brown (2016)	Housing First	Poor Mental Health (70.9%) and Substance abuse issues (75.8%)	182	Mean, 42.79; *SD*, 11.14	Male: 73.6%; Female: 26.4%	Non‐RCT	High unconditional
Buchanan (2006)	Respite Care	Poor Physical Health	225	Mean (*SD*), Intervention, 43 (9); Control, 44 (10)	Male: Intervention, 78% (125); Control, 81% (52). Female: Intervention, 22% (36); Control, 19% (12)	Non‐RCT	Moderate conditional
Buchanan (2009)	The Chicago Housing for Health Partnership (CHHP)	Poor Physical Health and substance abuse issues	94	Intervention, mean 45 (*SD* 6.9); Control, mean 43 (*SD* 7.7)	Male: Intervention, 39 (72%); Control; 43 (84%). Female: Intervention, 15 (28%); Control, 8 (16%)	RCT	High—conditional and unconditional, depending on participant
Cheng (2007)	HUD‐VASH	Poor Physical Health and substance abuse issues	460	Not specified	Not specified	RCT	Moderate unconditional
Gilmer (2010)	Full‐Service Partnerships (FSP)	Poor Mental Health and Incarceration	363	Mean (*SD*), Intervention, 44 (9); Control, 43 (11)	Male: Intervention, 131/209 (63%); Control, 97/154 (63%). Female: Intervention, 78/209 (37%); Control, 57/154 (37%)	Non‐RCT	High conditional
Goering (2011)	Chez Soi—Housing First	Poor Mental Health	2131	40.89 (*SD*, 11.23)	Male: 1508 (67.9%); Female: 603 (31.2); Other: 20 (.9%)	RCT	High unconditional
Goldfinger (1999)	Group homes, or Independent Apartments.	Poor Mental Health and Substance abuse issues	110	Mean, 38	(Intervention only) Male: 85 (72%); Female 33 (28%)	RCT	High unconditional
Gulcur (2003)	HOUSING FIRST Continuum of Care	Poor Mental Health and Incarceration and Substance abuse issues	199	Not specified	Male: 173; Female: 52	RCT	High unconditional
Howard (2011)	Crisis House or Patient preference of Crisis House	Poor Mental Health	44	Mean (*SD*), 37.5(11.1)	Female: 102 participants (100%)	RCT	Moderate conditional
Hwang (2011)	Supportive housing	None specified	112	22 people aged 17–0, 90 people aged 31 and over	81 male 72% 30 (27%) women one (1%) transgendered individual	Non‐RCT	High conditional
Kertesz (2007)	ACH Vs NACH Vs Control	Poor psychical Health, poor mental health, incarceration and substance abuse issues.	99	Mean (*SD*): ACH: 38.25 (2.61); N‐ACH: 41.25 (3.19); Control: 38.5 (2.61)	Male: ACH, 74.6% (47); N‐ACH, 76.8% (50); Control/No Housing, 76.8% (50). Female: ACH, 25.4% (16); N‐ACH, 24.2% (16); Control/No Housing, 24.2% (16)	RCT	Intervention 1, high conditional. Intervention 2, high unconditional
Larimer (2009)	Housing First	Poor Physical health and substance abuse issues	134	Mean (*SD*), Overall, 48 (10); Intervention: 48 (9); Control: 48 (11)	Male, 94% (126); Female, 6% (8)	Non‐RCT	High unconditional
Levitt (2013)	Home to stay	None Specified		Mean (SD), Intervention: 33.5 (8.7); Control: 33.9 (7.2)	Not specified	RCT	Moderate conditional
Lim (2017)	NYNY IIII	Substance Abuse Issues, care leaver and high risk of harm and/or exploitation	895	Mean overall: 18.6	Male, 510; Female, 385	Non‐RCT	High unconditional
Lim (2018)	New York Supportive Housing Program	Poor Mental Health and Substance abuse issues	330 families/2827 individuals	Number of people in each group: 18–34 years, 16%; 35–44 years, 26%; 45–54 years, 38%; ≥55 years, 20%	Male, 70% of 2827; Female, 30% of 2827	Non‐RCT	High unconditional
Lipton (2000)	Intervention 1: High Intensity Housing Intervention 2: Moderate Intensity Housing Intervention 3: Low Intensity Housing	Poor Mental Health and Substance abuse issues	2937	Mean (*SD*), 40.3(10.3)	Male 67% *n* = 1980; Female 33%, *n* = 957	Non‐RCT	Intervention 1, high conditional. Intervention 2, moderate conditional
McHugo (2004)	Integrated housing services programme	Poor Mental Health	113	Ranged, 21–60 years	Male: Intervention, 29 (47.5%); Control, 29 (48.3%). Female: Intervention, 32; Control, 31	RCT	High unconditional
Milby (1996)	Enhanced Care	Substance abuse issues	176	Age, mean (*SD*) Usual Care, 35.7 (6.2); Enhanced Care, 36.0 (6.6)	Male Intervention 54 (87.1%) Usual Care 50 (72.5%) Female Usual Care 8 (12.9) Enhanced Care 19 (27.5)	RCT	High conditional
Milby (2000)	Day treatment+	Poor Mental Health and Substance Abuse Issues	110	Mean (*SD*), DT, 39.1 (7.5); DT+, 37.3 (7.2); Al, 38.1 (7.4)	Male: DT, 45 (83%); DT+, 39 (70%); All, 84 (76%). Female: DT, 9 (17); DT+, 17 (30); All, 26 (24%)	RCT	High conditional
Milby (2008)	CM and CM+ (Contingency Management plus behavioural day treatment)	Substance abuse issues	206	CM, 39.5 (7.2); CM+, 40.6 (7.1)	Male: CM, 77 (74.8%); CM+, 73 (70.9%). Female: CM, 26 (25.2); CM+, 30 (29.1	RCT	High conditional
O'Connell (2012)	Housing and Urban Development–Veterans Affairs Supported Housing (HUD‐VASH) Intensive Care Management (ICM)	Poor physical Health, Poor mental health, substance abuse issues	207	Age (median + *SD*), TAU, 42.3 + −7.5; ICM, 44.0 + −6.3; HUD‐VASH, 41.8 + −7.1	100% Male	RCT	Moderate unconditional
Sadowski (2009)	Housing and Case management (HOUSING FIRST)	Poor mental health and substance abuse issues	357	25 and over	Male, 310/405 75%; Female, 95/405 25%	RCT	High unconditional
Shern (1997)	New York Street Study—Specialised Housing	Poor Mental Health	168	37.5 (9.01) years	Male, 72%; Male, (644). Female, 28%; Female, (250)	RCT	Moderate conditional
Siegel (2006)	Supported Housing (SH) vs community residences (CR)	Poor Physical Health, poor mental health and Substance abuse issues	47	Mean (*SD*): Stratum 1 (N‐10): SH, 34.7 (7.9); CR, 36(7.9) (n‐37). Stratum 2: SH, 41.6 (11.2) (N‐18); CR, 41.3 (10) (N‐28). Stratum 3: SH, 47.4 (10.1) (N‐39); CR, 41.6 (8.5) (N‐7)	Male: 91/139 across three strata of interventions. Female: 48/139 Across three strata of interventions	Non‐RCT	Intervention 1, high unconditional. Intervention 2, high conditional
Sosin (1996)	Housing and case management Case management alone	Substance abuse issues	419	Mean, 35; Housing, 35.5; case management, 35.2; control, 34.6	Male: 74.5% 312. Female: 25.5% 107	Non‐RCT	Moderate conditional
Srebnik (2013)	Housing First	Poor Physical Health, poor mental health and substance abuse issues	60	Intervention mean, 51.3; SD, 9.2. Control mean, 50.0; SD, 6.9	Male: 52 (87%); Intervention, 21 (72%); Comparison, 31 (100%); Female: 8 (13%); Intervention, 8 (28%); Comparison, 0 (0%)	Non‐RCT	High unconditional
Stefancic and Tsemberis (2007)	Pathways Consortium (Housing First)	Poor mental health and substance abuse issues	392	over 18 to be eligible, otherwise not specified	Male:74.23% 193; Pathways, 71, 67.6%; Consortium, 83, 79.8%; Control, 39, 76.5%. Female: 25.77%. 67; Pathways, 34, 32.4%; Consortium, 21, 20.2%; Control, 12, 23.5%	RCT	High unconditional

##### Descriptive account of reported accommodation interventions

As presented in Table 2, interventions varied considerably between studies, with some evaluating Housing First interventions (e.g., Brown et al., 2016; Goering et al., 2011) and others evaluating accommodation with specific services like case management (e.g., Sosin et al., 1996) and enhanced care (Milby et al., 1996). The most common aspect of the interventions was providing accommodation alongside some other form of additional service such as case management (e.g., Sosin et al., 1996), continuum of care (e.g., Gulcur et al., 2003), and other services delivered through a supportive housing approach (e.g., Lipton et al., 2000).

All the interventions aimed to improve outcomes for those individuals experiencing homelessness by focusing primarily on providing some form of housing. Although the interventions shared the same basis, the theories of change varied due to the other, additional, services that may or may not have been offered to participants. Some focused more on addressing adherence to medical care services (e.g., Appel et al., 2012; Buchanan et al., 2006; Buchanan et al., 2009) while others focused primarily on improving housing stability outcomes (Cheng et al., 2007; Lim et al., 2017; Srebnik et al., 2013).

See Table 3 for frequencies of effect sizes and number of studies which measured each of the five outcomes of interest. Table 4 demonstrates the diversity of outcomes covered by these accommodation‐based approaches and provides detail on how primary study authors described the outcomes measured in the included studies. Appendix B contains a table which provides additional details on the included studies including the geographical spread of the intervention (Scattered vs. congregate), and the years in which the intervention was delivered.

**Table 3:** Frequencies of effect sizes per intervention type

Intervention type	Housing stability (59)^a^	Health (65)	Crime and justice (12)	Employment and income (13)	Capabilities and wellbeing (23)
Basic/conditional (0 studies)^b^	6	2			
Basic/unconditional (0 studies)	7	2	5		
Housing only/conditional (0 studies)					
Housing only/unconditional (0 studies)	4	5		2	9
Moderate support/conditional (six studies)	5	6			3
Moderate support/unconditional (two studies)	16	22			4
High support/conditional (nine studies)	5	11	3	4	3
High support/unconditional (15 studies)	45	33	9	8	9
No intervention (control groups) (25 studies)	30	47	7	12	20

^a^ Outcomes are followed by the number of effect sizes in parentheses. For example, there are 59 effect sizes for outcomes related to housing stability. The total number of effect sizes presented in the table will be double the number of outcomes measured as we calculate one effect size per group.

^b^ As this is a network meta‐analysis, sometimes the control group will be an alternative treatment, such as basic conditional, which is why there are available effect sizes, even where it says 0 studies. We define a study by the typology of the intervention group only, as demonstrated in Table 2.

**Table 4:** Outcomes as described by primary authors

Outcome domain	Studies which measured this outcome	Outcomes as described by primary authors
Capabilities and wellbeing	Gilmer (2010) Goering (2012) Howard (2011) McHugo (2004) O'Connell 2012) Shern (2000)	Suicide Victimisation Quality of life Functioning (globally assessment functioning) Life satisfaction Social contact Psychiatric symptoms Community functioning
Crime and justice	Gilmer (2010) Larimer (2009) Sadowski (2009) Srebnik et al. (2013)	Incarceration Use of justice system services Number of days in prison
Employment and income	Goering (2012) Kertesz (2007) Milby (2010) O'Connell (2012)	Number in stable employment Number of days in stable employment Number of days worked Hourly wage
Health	Appel (2012) Brown (2016) Buchanan (2006) Buchanan (2009) Goering (2012) Gilmer (2010) Howard (2011) Larimer (2009) Lim et al. (2017) McHugo (2004) Milby (2000) Milby (2005) O'Connell (2012) Sadowski (2009) Srebnik et al. (2013)	Diagnosed STI rates Substance use Victimisation Number of days in institutional settings Period of hospitalisations Number of emergency department visits Mental health (measured using several scales) Physical health (measured using several scales) Health services used Inpatient days Abstinence
Housing stability	Appel (2012) Brown (2016) Buchanan (2006) Buchanan (2009) Gilmer (2010) Goering (2012) Goldfinger (1999) Gulcur (2003) Howard (2011) Kertesz (2007) Larimer (2009) Lim et al. (2017) McHugo (2004) Milby (2000) Milby (2010) O'Connell (2012) Sadowski (2009) Shern (1997) Siegel et al. (2006) Srebnik et al. (2013) Stefancic and Tsemberis (2007)	Periods of time spent homeless Stable housing Participants housed Time spent homeless Time spent in specific residential setting Days in institution Days homeless

#### 5.1.3 Excluded studies

All studies removed during screening had a tag assigned and are stored in the project on the EPPI‐reviewer software.

### 5.2 ROB in included studies

Assessment of methodological quality and potential for bias was conducted using the second version of the Cochrane Risk of Bias tool for RCTs (Higgins et al., 2019). The 16 studies in this review that are labelled as RCTs were assessed for ROB and placed into one of three categories from the Cochrane ROB tool: low ROB, some concerns and high ROB. Nonrandomised studies were coded using the ROBINS‐ I tool (Sterne et al., 2016). The 12 studies in this review that are labelled as non RCT's were assessed in their ROB and placed into one of four categories from the ROBINS‐I tool, low, moderate, serious and critical.

**Figure 2:**
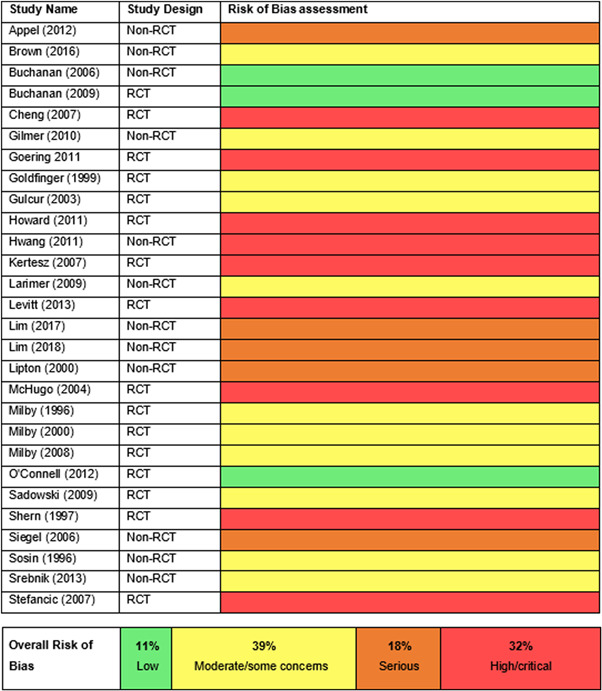
Risk of bias

Out of the 28 studies, three had sufficiently low ROB (11%), 11 (39%) had moderate ROB, five (18%) presented serious problems with ROB, and nine (32%) demonstrated high, critical problems with their methodology. Figure 2 provides a visual overview of the ROB assessment for all included studies.

### 5.3 Effects of interventions

The analyses presented utilised a REM, estimating the variance component with restricted (or residual, or reduced) maximum likelihood (REML). The REM was chosen as the statistical model as it accepts two main differences among primary studies, the first is within study variance, and the second is between study variance. This between study variance, or heterogeneity, can reflect important differences in populations, settings, or progression of time (Borenstein et al., 2009). To allow for estimation of the variance components, the Satterthwaite approximation was used to account for two different sample variances where only estimates of the variance are known. The analysis is useful to calculate an approximation to the effective degrees of freedom (Satterthwaite, 1946).

When conducting meta‐analysis on the effectiveness of accommodation‐based interventions, we were attentive to whether different types of accommodation‐based intervention (as defined by our typology) are more or less effective for individuals experiencing homelessness. Few of the included trials compared the effects of two interventions directly (*n* = 11) and so direct comparisons between some accommodation‐based interventions do not exist, however the majority of interventions were tested against equivalent control groups. Thus, through NMA, it is possible to calculate the indirect effects of comparative accommodation‐based interventions and produce this as a “network” of comparisons. These analyses were completed via a frequentist model using R package, *netmeta*, and are reported below.

In addition, we include moderator analyses that test whether specific characteristics of the studies or the interventions can explain some of the heterogeneity in results. It is important to understand that moderator analyses are exploratory and should never be implemented to test hypotheses. Even if the meta‐analysis contains only studies with specific methodologies (RCTs and quasi‐experiments), the studies involved in these moderator analyses have not been randomised, they are observational in nature and at a higher ROB. Additionally, these types of analyses generally have lower power due to missing data in the primary research, there is an increased risk of presenting incorrect results which appear simply through chance (false positive conclusion), and potential for various biases. Although these analyses are a common inclusion to many meta‐analyses as they are useful for developing ideas and exploring heterogeneity, moderator analysis have low statistical power and should always be interpreted with caution (Borenstein et al., 2009).

#### 5.3.1 Housing stability

##### Network meta‐analysis

**Figure 3:**
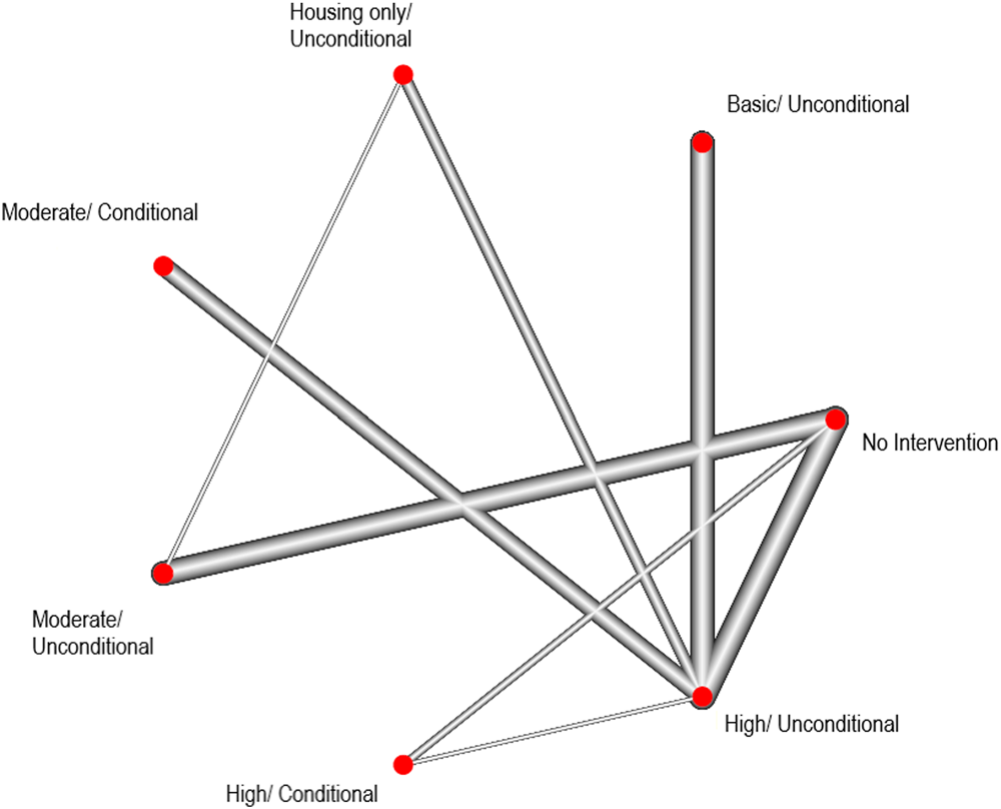
Network diagram of comparisons with the (no intervention) control group on housing stability outcomes

Using the available data from 21 studies which contained 59 measures of housing stability, it was possible to conduct a network meta‐analysis to estimate the relative effect of different categories of intervention (described by the typology), on housing stability. These head‐to‐head comparisons are shown in Figure 3 and in Table 5. When the numbers in the table are negative, it means that the intervention in the row had worse outcomes than the intervention in the column. The first number denotes the point estimates, while the number in brackets present the confidence intervals. The confidence intervals can be understood as “good” and “bad” scenarios that are also reasonably in line with the data.

**Table 5:** Estimates of pairwise differences in housing stability between each category of the intervention typology

Intervention type	Housing/unconditional	Moderate/conditional	Moderate/unconditional	High/Conditional	High/Unconditional	No intervention
Basic/unconditional	−0.97 (−2.15, 0.21)	−0.83 (−1.84, 0.18)	−0.87 (−1.87, 0.13)	−0.69 (−1.71, 0.32)	−1.10 (−1.82, −0.39)	−0.48 (−1.32, 0.36)
Housing only/unconditional	.	0.14 (−1.04, 1.31)	0.10 (−0.85, 1.06)	0.28 (−0.85, 1.41)	−0.13 (−1.07, 0.81)	0.49 (−0.46, 1.45)
Moderate/conditional		.	−0.03 (−1.03, 0.96)	0.14 (−0.87, 1.15)	−0.27 (−0.98, 0.44)	0.35 (−0.48, 1.18)
Moderate/unconditional			.	0.17 (−0.69, 1.04)	−0.24 (−0.93, 0.46)	0.39 (−0.19, 0.97)
High/conditional				.	−0.41 (−1.13, 0.31)	0.21 (−0.44, 0.86)
High/unconditional					.	0.62 (0.19, 1.06)

Two categories of interventions (Basic (Conditional) and Housing Only (Conditional)) did not have sufficient numbers of studies for head‐to‐head comparisons and so these are not included in Table 5.

Based on the point estimates, some important trends are noteworthy, even if only indicative as the confidence intervals for most comparisons remain wide suggesting that there is still substantial uncertainty around the plausible “good” and “bad” scenarios.

First, in the row categorised as Basic/Unconditional support, which describes interventions that offer only a bed (alongside some very basic sustenance such as an evening meal) suggests that all other available categories performed better for outcomes related to housing stability, even in the no intervention groups. Second, in the column categorised as high/unconditional support, which describes interventions than offer accommodation alongside assertive and individualised support, results suggest that this type of intervention provided better outcomes on housing stability than other available categories of intervention. Finally, we can see that all interventions performed better than no intervention, except for the group of interventions categorised as basic/unconditional.

Two of these comparisons of intervention categories were statistically significant (shaded in light grey):

1. Interventions classified as *high support and unconditional* resulted in greater housing stability than interventions classified as *basic and unconditional*. The effect size describing this difference is 1.10^1^ (95% CI [0.39, 1.82]).
2. Interventions classified as *high support and unconditional* resulted in greater housing stability than no intervention. The effect size describing this difference is 0.62 (95% CI [0.19, 1.06]).

**Figure 4:**
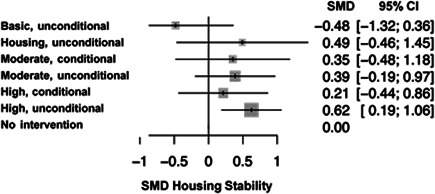
Forest plot of comparisons with the (no intervention) control group (housing stability)

We have the most information about comparisons with the no intervention control group. The forest plot in Figure 4 shows these estimated effects, comparing each intervention with the no‐treatment control.

Forest plots, which are the graphical representation of a meta‐analysis, present effect sizes in a way that provides simple interpretation of effectiveness. From Figure 4, one can quickly assess that five studies included in this analysis were not statistically significant as all have CI's which cross the line of no effect. In this review, the effect size metric used was the standardised mean difference, represented by square boxes. The size of the boxes is representative of the weight of the study, larger boxes mean more weight, while smaller mean less. The combined SMD of all the primary studies is represented by the black diamond at the bottom of the graph.

The only statistically significant comparison is with interventions that are categorised as *high support and unconditional*. As previously reported, this effect size is 0.62 (95% CI [0.19, 1.06]), in favour of the intervention.

##### Moderator analyses

Earlier, in Table 4, we see that 21 studies looked at outcomes related to housing stability. These outcomes include measures of periods of time spent homeless, stable housing, participants housed, time spent in specific residential setting, days in institution and days homeless. Table 3 reports the number of housing stability effect sizes per comparison of intervention categories. In order to conduct a moderator analysis, it is necessary to have a sufficient number of effect sizes within each comparison. Due to missing outcome data in the primary research, there is an increased risk of presenting incorrect results which appear simply through chance (false positive conclusion) (Hedges & Pigott, 2009). Thus, moderator analyses were attempted for only two comparisons with sufficient data (Table 6):

1. *Moderate support and unconditional* interventions versus no intervention (10 effect sizes)
2. *High support and unconditional* versus no intervention (16 effect sizes)

**Table 6:** Number of effect sizes per comparison of intervention categories

Intervention type	Basic/unconditional	Housing/unconditional	Moderate/conditional	High/conditional	High/unconditional	No intervention
Moderate/unconditional	0	2	0	0	0	10
High/conditional	0	0	0	1	1	5
High/unconditional	7	3	7	0	4	16

###### Moderate support and unconditional interventions versus no intervention

Although there were 10 effect sizes comparing interventions categorised as *moderate support and unconditional* with no intervention control, these were from only four studies. Thus, no moderator analyses were possible.

###### High support and unconditional interventions versus no intervention

There were 16 effect sizes from seven studies that compared interventions categorised as *high support and unconditional* with no intervention control. Subgroup analyses were conducted to investigate whether study design, study quality, age or the geographical spread of housing (scattered site vs. congregate) moderated the effectiveness of high support/unconditional interventions (compared to no intervention) on housing stability outcomes. There were insufficient data to explore the moderating influence of complexity of need and whether the intervention was focussed on families or individuals (as specified in the methods section).

Study Design: For these 16 effect sizes, six were from nonrandomised studies and 10 were from RCTs. There was no difference in effect size between non‐RCT and RCT studies.

Study Quality: The same was true when ROB was used as the moderator variable. There were four studies rated as moderate ROB and 12 studies rated as high ROB. There was no statistically significant difference in the mean effect size between these two groups.

Age: Nine of the 16 studies included an estimate of the age of the participants, coded as an integer. Age was not significantly related to effect size magnitude in these studies.

Geographical spread of housing: In this moderator analysis we have five effect sizes which are scattered site housing, and four that are congregate. Geographical spread was not significantly related to effect size magnitude in these studies.

#### 5.3.2 Health outcomes

##### Network meta‐analysis

**Figure 5:**
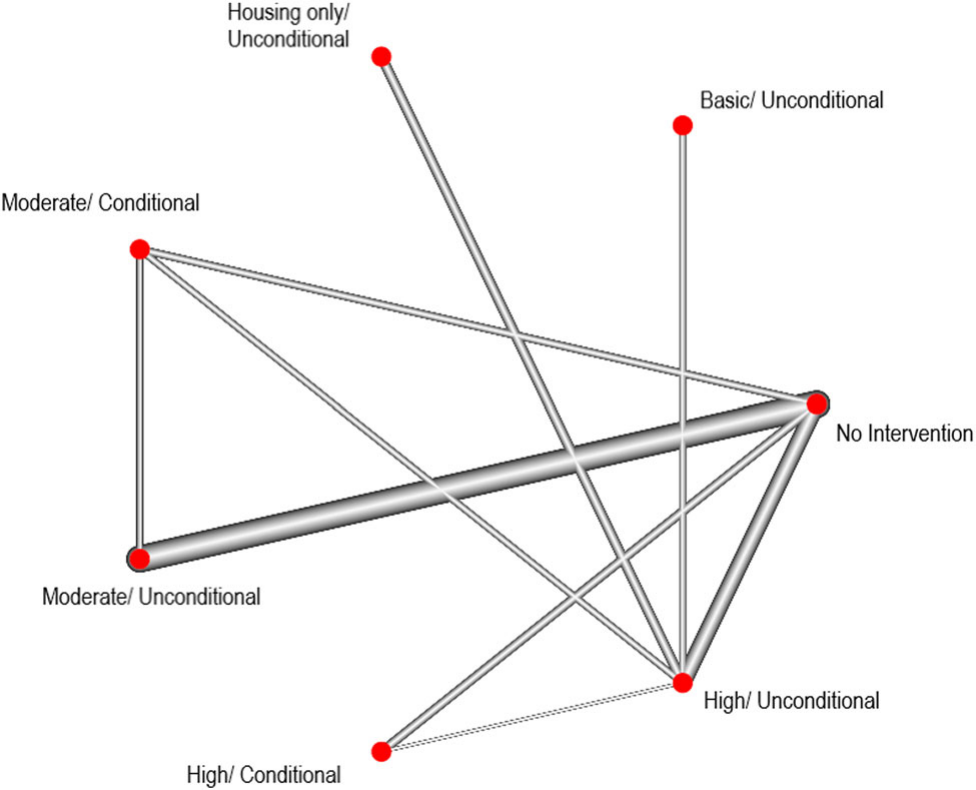
Network diagram of comparisons with the (no intervention) control group across health outcomes

There were 65 measures of treatment effect across 20 studies, therefore, it was possible to conduct a network meta‐analysis to estimate the relative effect of different categories of intervention (described by the typology), on health outcomes. These head‐to‐head comparisons are shown in Figure 5 and in Table 7. As in the previous analysis (housing stability outcomes) some obvious trends emerge when considering the point estimates. However, as above, the confidence intervals which denote “good” and “bad” scenarios, that are also consistent with the data, remain wide. First, Basic/Unconditional interventions again performed worse for health outcomes than all other interventions including no intervention groups. Also, noteworthy that all interventions performed better than no intervention, except for the interventions categorised as basic/unconditional. Two comparisons were statistically significant (shaded in light grey):

1. Interventions classified as *moderate support and conditional* resulted in better health outcomes than no intervention. The effect size describing this difference is 0.36 (95% CI [0.03, 0.69]).
2. Interventions classified as *high support and unconditional* resulted in better health outcomes than no intervention. The effect size describing this difference is 0.22 (95% CI [0.01, 0.43]).

**Table 7:** Estimates of head‐to‐head comparisons in health outcomes between each category of the intervention typology

Intervention type	Housing/unconditional	Moderate/conditional	Moderate/unconditional	High/conditional	High/Unconditional	No intervention
Basic/unconditional	−0.19 (−0.94, 0.55)	−0.45 (−1.00, 0.09)	−0.36 (−0.92, 0.20)	−0.31 (−0.85, 0.23)	−0.32 (−0.75, 0.12)	−0.10 (−0.57, 0.38)
Housing/unconditional	.	−0.26 (−0.95, 0.43)	−0.17 (−0.87, 0.54)	−0.12 (−0.80, 0.57)	−0.12 (−0.73, 0.48)	0.10 (−0.54, 0.74)
Moderate/conditional		.	0.09 (−0.30,0.49)	0.15 (−0.28, 0.57)	0.14 (−0.19, 0.47)	0.36 (0.03, 0.69)
Moderate/unconditional			.	0.05 (−0.37, 0.47)	0.04 (−0.32, 0.41)	0.27 (−0.05, 0.58)
High/conditional				.	−0.01 (−0.33, 0.31)	0.21 (−0.06, 0.49)
High/unconditional					.	0.22 (0.01, 0.43)

**Figure 6:**
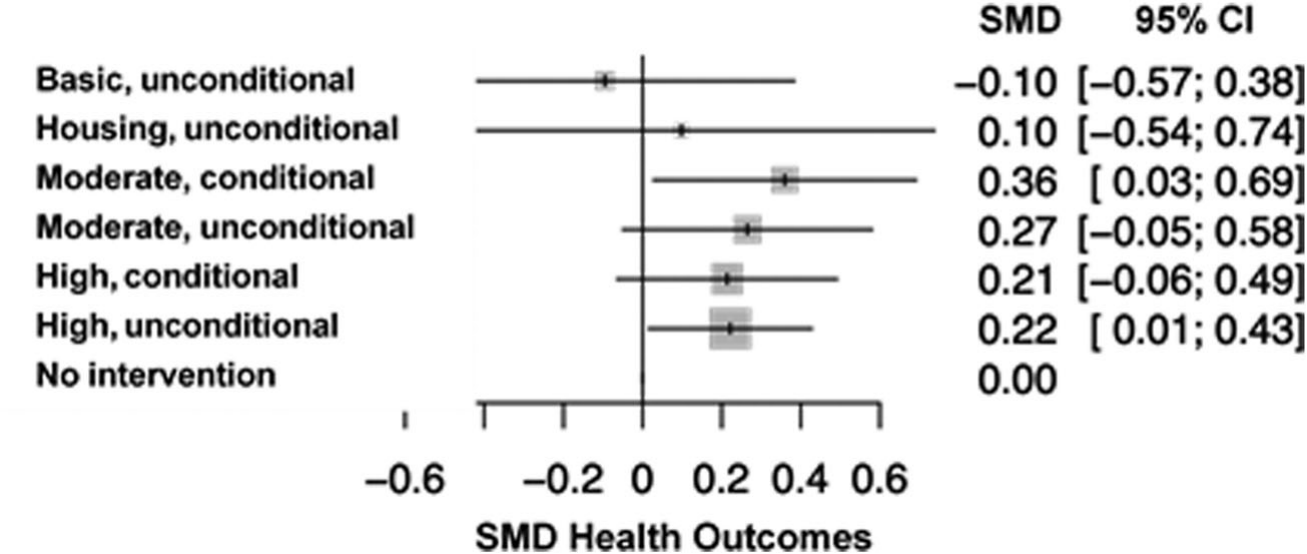
Forest plot of comparisons with the (no intervention) control group (health outcomes)

The forest plot in Figure 6 shows the estimated effects associated with comparing each intervention with the no‐treatment control. The only statistically significant comparisons are those already identified in Table 7, that is, interventions classified as either *moderate support and conditional* or *high support and unconditional*, resulted in better health outcomes for participants compared to a no intervention control.

#### 5.3.3 Moderator analyses

Table 8 reports the number of health‐related effect sizes per comparison of intervention categories. Moderator analyses were attempted for the two comparisons that had sufficient number of effect sizes:

1. *Moderate support and unconditional* interventions versus no intervention (16 effect sizes)
2. *High support and unconditional* versusvs no intervention (20 effect sizes)

**Table 8:** Number of effect sizes per comparison of intervention categories

Intervention type	Basic/unconditional	Housing/unconditional	Moderate/conditional	Moderate/Unconditional	High/conditional	No intervention
Moderate/conditional	0	0	0	6	0	3
Moderate/unconditional	0	0	0	0	0	16
High/conditional	0	0	0	0	0	7
High/unconditional	3	5	4	0	1	20

##### Moderate support and unconditional interventions versus no intervention

There were 16 effect sizes that compared the interventions categorised as *moderate support and unconditional* with no intervention control. These 16 effect sizes were from three studies and so no subgroup analysis was possible.

##### High support and unconditional interventions versus no intervention

There were 20 effect sizes that compared the interventions classified as *high support and unconditional* with no intervention control. These 20 effect sizes were from seven studies. The same subgroup analyses described above were also conducted for the health outcomes.

Study Design: For these 20 effect sizes, 11 were from nonrandomised studies and nine were from randomised controlled studies. There was no difference in effect size between non‐RCT and RCT studies.

Study Quality: Similarly, when using ROB as the moderator variable, there was no difference in the mean effect size between the 10 studies rated as moderate ROB and the 10 studies rated as high.

Age: Twelve of the 20 studies included an estimate of the age of the participants, coded as an integer. Age was not significantly related to effect size magnitude in these 20 studies.

Geographical spread of housing: In this moderator analysis we have two effect sizes which are scattered site housing, and nine that are congregate. Geographical spread was not significantly related to effect size magnitude in these studies.

#### 5.3.4 Crime and justice outcomes

There were five primary studies which measured 12 outcomes related to Crime and Justice. All five primary studies fell into either the High/Unconditional or High/Conditional category of housing intervention. Intervention groups were compared with control groups who received either Basic/Unconditional, waitlist, no treatment, or standard care services. The outcomes measured via these experiments included measures of number of days spent in prison/jail, conviction, arrest, and imprisonment.

**Figure 7:**
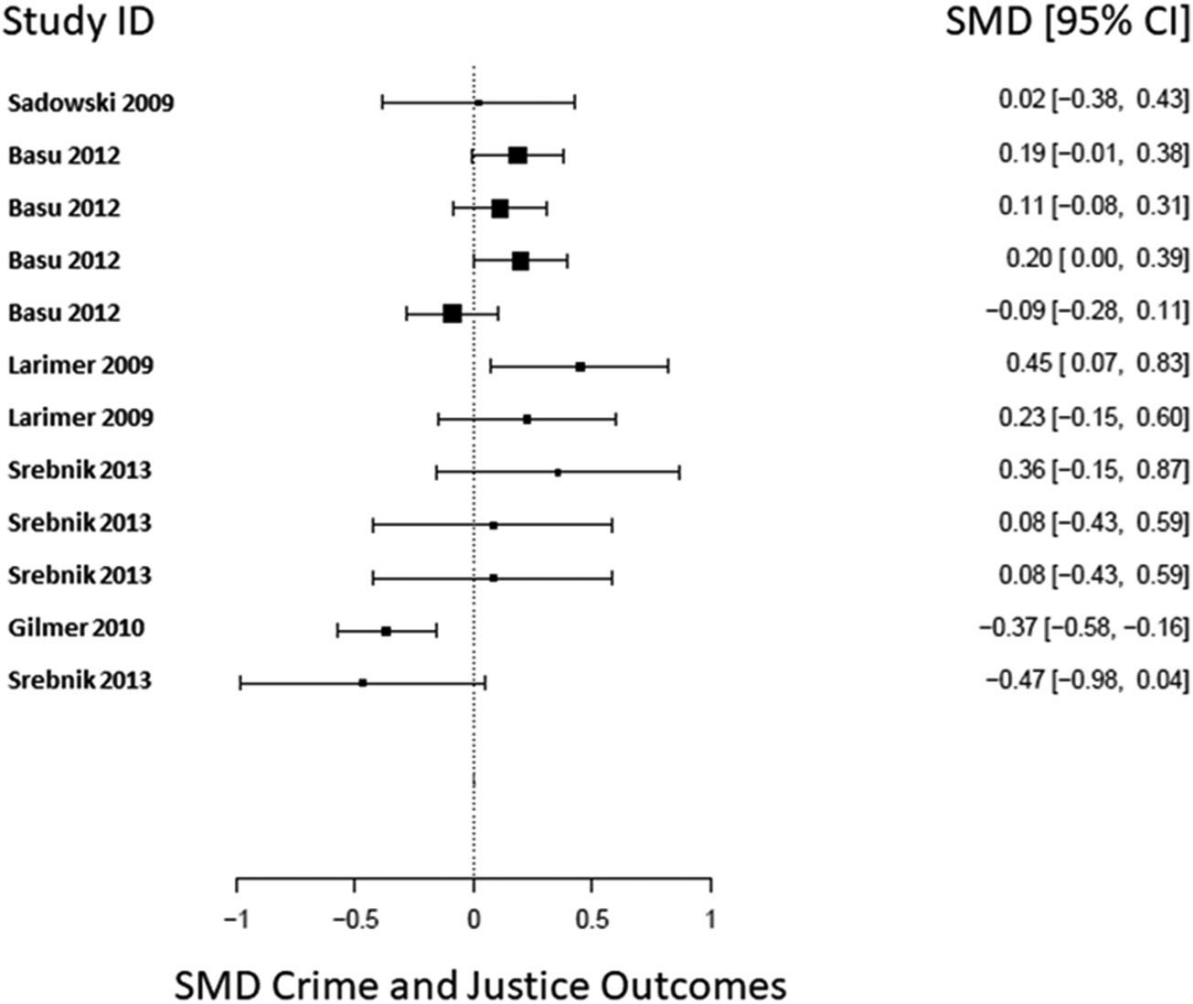
Forest plot of studies including crime and justice outcomes

The forest plot in Figure 7 shows the estimated effects associated with comparing accommodation‐based approaches with control groups. As shown in the forest plot in Figure 7, two studies had large sample sizes (Basu et al., 2012 (*n* = 201); Gilmer et al., 2010 (*n* = 209)). Smaller sample sizes, such as those presented in the Srebnik et al. (2013) study (*n* = 29), have wider confidence intervals, representing more variance.

Only one (8%) of the included 12 effect measures of Crime and Justice had an SMD which was statistically significant and favoured the intervention group (Gilmer et al., 2010: SMD = −0.37, CI [−0.58 to −0.16]). This study compared a High/Conditional intervention group to a control group not receiving an intervention. The outcome measured was the likelihood of using justice system services.

#### 5.3.5 Employment outcomes

There were five primary studies which measured 13 outcomes related to Employment. All five primary studies fell into either the High/Unconditional, High/Conditional, or Moderate/Unconditional category of housing intervention. Comparison groups received either High/Unconditional or standard care services. The outcomes measured via these experiments included measures of number of individuals in stable employment, number of days in stable employment, number of days worked, and hourly wage.

**Figure 8:**
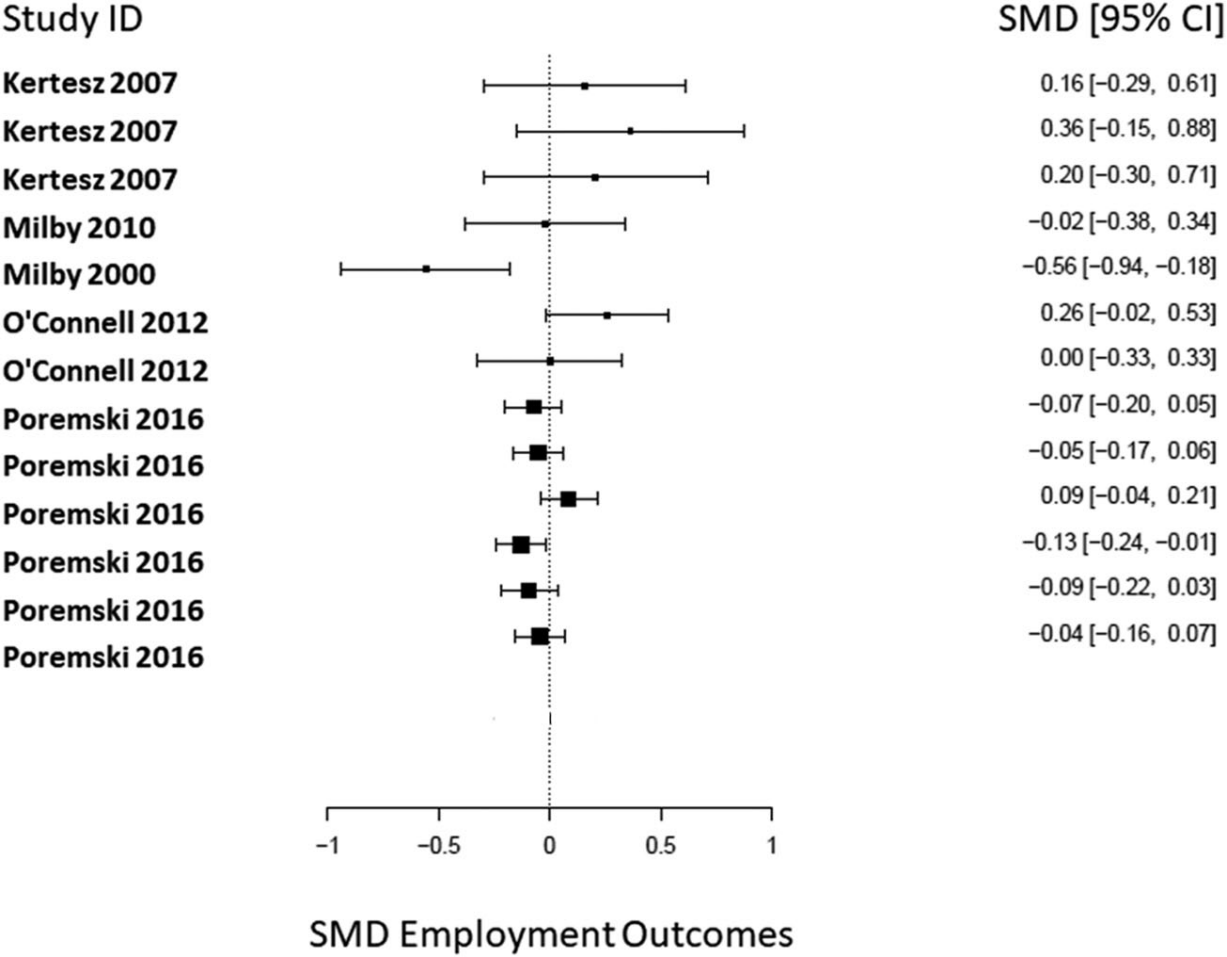
Forest plot of studies including employment outcomes

The forest plot in Figure 8 shows the estimated effects associated with comparing accommodation‐based approaches with control groups. As shown in the forest plot in Figure 8, one study had a large sample size (Poremski et al., 2016 (*n* = 689)). Smaller sample sizes, such as those presented in the Kertesz et al. (2007) study (*n* = 45), have wider confidence intervals, representing more variance.

Two (15%) of the included 13 effect measures of Employment had SMDs which were statistically significant and favoured the control groups (Milby et al., 2000; Poremski et al., 2016). The Milby study (Milby et al., 2000) measured employment as the percentage of days in full time employment in the past 60 days. They compared the mean difference between the intervention group which received High/Conditional support against a comparison group which received standard care. Poremski et al. (2016) compared an intervention group which received High/Unconditional support against a control group which received standard care. This study measured this employment outcome by asking participants the number of hours worked per week.

#### 5.3.6 Capabilities and wellbeing outcomes

There were 10 primary studies which measured 23 outcomes related to capabilities and wellbeing. All 10 primary studies provided interventions which met the criteria to be classified as high unconditional, Moderate/Conditional, or Moderate/Unconditional categories. Comparison groups received either Housing only/Unconditional, Moderate/Conditional, Moderate/Unconditional, or standard care. The outcomes measured via these experiments included, but were not limited to, measures of Quality of Life, life satisfaction, and social contact. Five of the effect sizes were presented as log‐odds ratios. All effect sizes were transformed to standardised mean differences for this presentation of the data.

**Figure 9:**
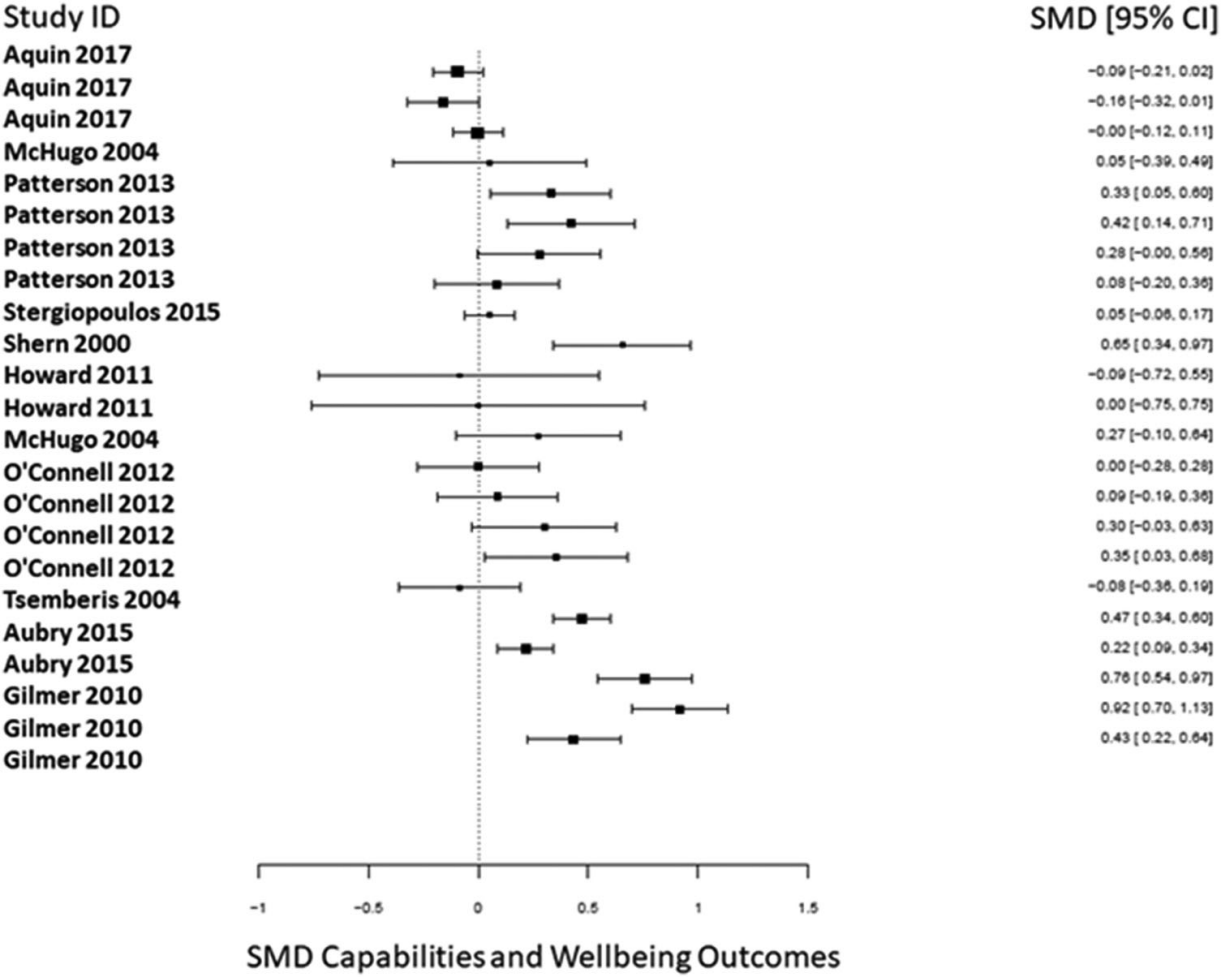
Forest plot of studies including capabilities and wellbeing outcomes

The forest plot in Figure 9 shows the estimated effects associated with comparing accommodation‐based approaches with control groups. As shown in the forest plot in Figure 9, three studies had large sample sizes (Aquin et al., 2017 (*n* = 1236); Gilmer et al., 2010 (*n* = 209); Stergiopoulos et al., 2015 (*n* = 689)). Smaller sample sizes, such as those presented in the Howard et al. (2011) study (*n* = 13), have wider confidence intervals, representing more variance.

**Figure 10:**
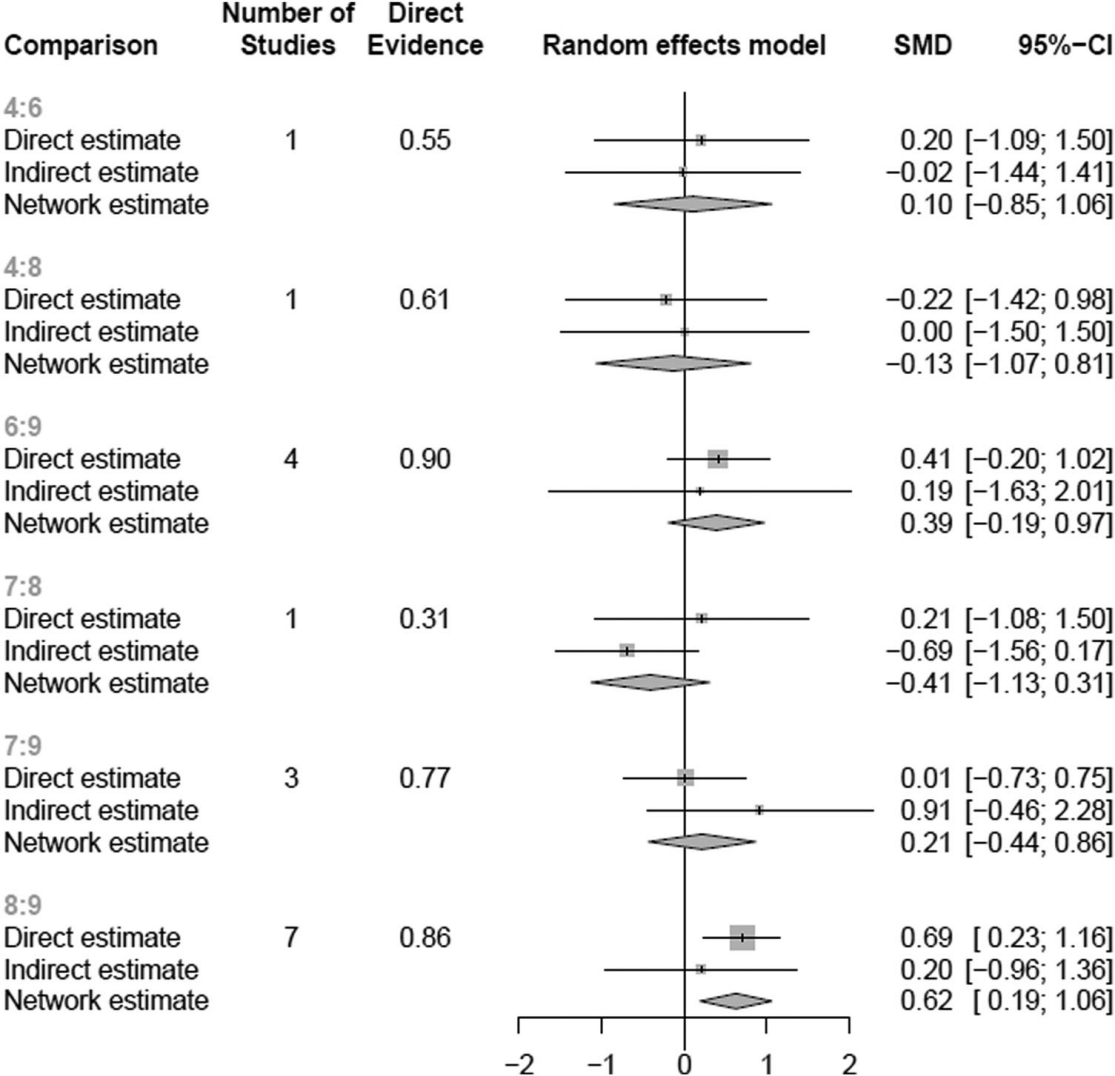
Comparison of direct and indirect estimates

Nine (39%) of the included 23 effect measures of capabilities and wellbeing had SMDs which were statistically significant. Of these studies, five were High/Unconditional, three were Moderate/Unconditional, and one was Moderate/Conditional.

#### 5.3.7 Cost analysis

The review team extracted cost data, where available. An overview of this is presented in Appendix C. In the studies that did include cost analysis and reported the outcomes, the intervention is generally more expensive, due to the acquisition and upkeep of property. However, this cost is, at least in part, offset due to the savings made in other settings, such as emergency departments and hospital inpatient care.

#### 5.3.8 Process and implementation synthesis

##### Background and aims

The second element of the current review involved synthesising qualitative data extracted from process evaluations included in CHI's implementation and process evaluation EGM. The purpose of this synthesis was to complement the quantitative evidence reported above and provide a better understanding of what factors influence programme effectiveness. It focused on the following question:

What implementation and process factors influence intervention effectiveness?

The typology used to construct the original EGM (White et al., 2018) was developed using a grounded theory approach piloted on 25 papers initially. This iterative process was combined with expert knowledge, ensuring that the broad concepts identified would adequately capture all papers included in the map. From the piloted typology, categories were created to include all process evaluations found during the searching period. The team in Heriot‐Watt coded each process evaluation under five main analytical categories of factors or levels of influence, namely: contextual factors, policy makers/funders, programme administrators/managers/implementing agencies, staff/case workers and recipients of the programme. Using a best fit synthesis framework, it is these five analytical categories that have been used to synthesise and organise the data analysis reported in the following section.

In this way, the EGM provided an initial framework around which to synthesise the data; a framework that, for the most part, fits better than anything else. This decision also ensured that the EGM structure could be used to inform the synthesis process but also provided the team with a degree of flexibility. This flexibility became an essential component to the review as the map captures barriers and facilitators to the process of implementing interventions whereas this review focuses on the process factors that impact upon the effectiveness of an intervention.

We included process evaluations and other relevant qualitative studies that provided data to enable a deeper understanding of why accommodation programmes, in general, do (or do not) work as intended, for whom and under what circumstances. Studies were selected on the basis of providing insight into implementing accommodation programmes with a diverse range of populations and geographical locations. Studies that provided most data were selected first and additional studies added until we reached saturation. There was no overlap between the studies in the effectiveness analysis and the qualitative papers.

##### Framework synthesis

Framework synthesis is an approach that originates from a process of analysing primary research data to address policy concerns. The background theoretical and empirical literature help create an understanding of the issue into an initial conceptual framework, which develops iteratively as new data are incorporated and themes are derived from the data. This process was carried out in collaboration with researchers and academics in Heriot Watt University and the Campbell Collaboration (White et al., 2018). This synthesis method presents an opportunity to use a “scaffold against which findings from the different components of an assessment may be brought together and organised” (Carroll et al., 2011, p. 1). Its flexibility captures new understanding as data are incorporated into the framework.

Framework synthesis comprises five methodological stages:

1. Familiarisation
2. Framework Selection
3. Indexing
4. Charting
5. Mapping and Interpretation

These stages are often overlapping and may be revisited throughout the process.

The first is the familiarisation stage in which a reviewer becomes familiar with current issues and ideas about the topic, by drawing iteratively on a variety of sources (Booth & Carroll, 2015). This leads to the second stage: framework selection where an initial framework is chosen, which might be a conceptual or policy framework, logic model, causal chain or established theory that might explain the issue (Bruton et al., 2020). During the third indexing stage, studies are searched for, screened and data extracted using the initial conceptual framework. Much of this work was carried out in the development of the Implementation issues EGM (White et al., 2018). Here, studies are sorted to determine their relevance to the review questions and to identify their main characteristics. During this stage, Campbell UK and Ireland screened the process evaluations for relevance to the review. During the fourth charting stage, the main characteristics of each study are analysed by grouping characteristics into categories and deriving themes directly from those data (Brunton et al., 2020). At this stage, a process of purposive sampling (Booth et al., 2016) was completed by Campbell UK and Ireland due to the available team expertise and resources. This purposive sample endeavoured to include process evaluations spanning geography, targeted populations and types of intervention in order to exhibit an accurate representation of accommodation programmes available. The selected process evaluations presented the most “rich” and “thick” data (Booth et al., 2016) from the studies included in the map. At this stage, Campbell UK and Ireland synthesised much of the available data from the selected studies against the original agreed framework embedded in the EGM. During the final stage of mapping and interpretation stage, the derived themes are considered in light of the original research questions (Brunton et al., 2020) and in this case, policy implications. This stage has been completed in collaboration with content experts who could consider these themes in light of the available empirical and theoretical literature.

#### 5.3.9 Results

##### Included papers

On 10th May 2019, 246 process evaluations were downloaded from the implementation and process EGM. Title and abstract screening of these evaluations for inclusion in this review was undertaken independently by the review team and 135 papers were identified as relevant to accommodation programmes for individuals experiencing or at risk of experiencing homelessness. Papers that considered a wide variety of factors from legislation and housing markets to perceptions held by services users were initially viewed for full text screening. From the 135 papers related to accommodation that were reconciled, 10 papers were selected for synthesis using purposive sampling to create a manageable and rich data set (Austin et al., 2014; Booth et al., 2016; Burt, 2009; Busch‐Geertsema, 2013; Greenwood et al., 2005; HRDC, 2003; Keast et al., 2008; Lawlor & Bowen, 2017; Pleace & Bretherton, 2013; Turner Research & Strategy, 2015; Sewel, 2016). These studies are presented in Tables 9 and 10 below.

**Table 9:** Characteristics of included studies

Study name	Name of intervention	Intervention type	Location	Setting	Population
Austin et al. (2014)	Housing and Urban Development Veteran's Affairs Supportive Housing	High/unconditional support	USA	Veteran's Affairs facilities	Veterans
Burt (2009)	Various permanent supportive housing projects	Not specified	USA	Various	Long term homeless and homeless populations with mental illnesses
Busch‐Geertsema (2013)	Housing First Europe	High/unconditional support	Europe	Amsterdam: Discus Housing First Copenhagen (ACT in Copenhagen)Glasgow (Turning Point Scotland Housing First) Lisbon (Casas Primeiro) Budapest (Pilisi Forest Project)	Homeless populations with complex needs
Greenwood (2005)	Dublin Housing First Demonstration Project	High/unconditional support	Ireland	Dublin	People with significant histories of homelessness
HRDC (2003)	National Homeless Strategy	Not specified	Canada	Various community‐ based case studies	People experiencing homelessness, particularly youth and Aboriginal groups
Keast (2008)	Responding to Homelessness Strategy	High/conditional support	Australia	Various service settings across Brisbane, the Gold Coast and Townsville	Various: newly homeless users, users with complex and multiple needs, users in persistent homelessness
Lawlor and Bowen (2017)	Limerick Youth Housing	High/unconditional support	Ireland	Limerick city and Midwest region of Ireland	Young people who are homeless or at risk of becoming homelessness
Pleace and Bretherton (2013)	Camden Housing First	High/unconditional support	UK	Camden hostels	People with co‐morbidities experiencing recurrent homelessness
Sewel (2016)	Supported lodgings services	High/conditional support	UK	Supported lodgings services for care experienced young people	Young people in care who are yet ready for independent living
Turner Research (2015)	Haven's Way Programme	High/conditional support	Canada	Haven's Way congregate housing	Vulnerable young women experiencing considerable housing instability

**Table 10:** Description of interventions

Study name	Theory of change	TAU	Tx Length	Dosage	Personnel delivering intervention	Personnel working with users	Intervention funding	Organisations involved	Participants
Austin et al. (2014)	To end veteran homelessness by 2015 using and Housing First model by providing permeant rental vouchers	NR	NR	NR	Housing First coordinator, HUD‐VASH programme manager	Housing specialist, Case workers, substance use or mental health specialist	Federal funding	DoH and Urban Development	95 facility managers and front‐ line staff interviewed
Burt (2009)	To reduce people experiencing long term homelessness with Predevelopment work on permanent supportive housing projects, collaborating with public officials and key stakeholders to stimulate increased commitment.	NR	Varied across the projects	Usually weekly meetings with case worker	Corporation for supportive housing	Case workers, health care specialists	Conrad N. Hilton Foundation	Los Angeles Council	35 staff and stakeholders interviewed
Busch‐Geertsema (2013)	To use the Housing First approach in various European cities to house individuals who are homeless	No control group	Varied between cities	Weekly home visits from staff	Case managers	Case managers	European Commission	Housing First Europe, National Board of Social Services, GISS Bremen	22 participants interviewed
Greenwood (2005)	To use Housing First (scattered site) in to house those who are currently homeless	Usual housing services	Three months at times to house	Weekly face to face meetings with programme staff	Team leader	Programme staff: key workers, substance specialist, nurse, employment support worker	European Commission	Dublin Region Homeless Executive	30 participants surveyed, 26 interviewed
HRDC (2003)	To provide housing and other services to vulnerable groups	No control groups due to ethical reasons	Varies between projects	Not described, probably varied across the projects	Various	Various	Federal funding	Local organisations to the projects	Interviews collected from 91 youth and Aboriginals
Keast (2008)	Integrate services to provide a smoother pathway to housing for people who are homeless	NR	NR	NR	Service staff	Homelessness workers	Queensland Government	DoH, Department of Communities, Queensland Health, Department of Justice and Attorney‐General, Queensland Police, Queensland Corrective Services, Department of Tourism, Fair Trade and Wine Industry Development	241 different projects but number of participants not specified
Lawlor and Bowen (2017)	The provision of secure, appropriate and supported accommodation allows young people the space and stability to plan for the future	NR	Varies from 3 to 18 months and more	Varied between twice a week and twice a month	Tusla and Focus staff	Key workers and social workers	Human Dignity Foundation, Focus Ireland and Tusla	Focus Ireland, Tusla, Limerick City and County Council	14 semi structured interviews with young people
Pleace and Bretherton (2013)	The provision of secure, appropriate and supported accommodation allows people the space and stability to plan for the future	NR	Up to 18 months	Weekly home visits	Camden Council staff	Case managers	Camden Council	Camden Council, University of York, SITRA	13 service users interviewed
Sewel (2016)	Providing care experienced young people with supported lodgings will assist in a young person developing the confidence and capability to live an independent adult life	NR	Up to 6 months before discharge	2‐3 face to face meetings per week with support worker, tapering to once a month as placement continues	Supported lodgings providers	Support worker	Local authority funding	Barnardos	7 service staff surveyed, 11 service staff, 14 young people and 20 supported lodgings providers interviewed
Turner Research (2015)	Providing young girls with supported lodgings will assist in a young person developing the confidence and capability to live an independent adult life	NR	Average of 8 months	Weekly case manager meetings	Boys and Girls Clubs of Calgary	Case manager	Safe Haven Foundation of Canada (Housing Firstc), private donors and Alberta Human Services.	Human Services, Government of Alberta, Boys & Girls Clubs of Calgary, Safe Haven Foundation	18 participants and 5 stakeholders interviewed

Abbreviations: DoH, department of housing; NR, none reported; TAU, treatment as usual; Tx, treatment.

Three process evaluations focus on using the Housing First programme to tackle homelessness. Another evaluation focuses on veterans accessing accommodation after discharging from the armed forces. One of the selected studies concentrates on people with mental health issues accessing appropriate accommodation. Three evaluations focus on young people as a target group, one of which focuses on ensuring care experienced young people move into secure and stable accommodation after “growing out” of care services; two others target a more general population. One of the selected studies are based on interventions conducted in the UK, two in Ireland, one in Australia, one across Europe and the remaining five were carried out in North America; three in the United States and two in Canada. All evaluations took place between 2003 and 2017.

The following analysis takes each of the five main analytical categories of factors or levels of influence (described above and reflected in the EGM) in turn, namely: contextual factors, policy makers/funders, programme administrators/managers/implementing agencies, staff/case workers and recipients of the programme.

##### Quality appraisal of included studies

The quality appraisal of the selected process evaluations was carried out using the tool developed by White and Keenan (2018) in collaboration with CHI. This tool assesses the quality of each of the 10 process evaluations by asking a series of questions regarding methodology, data analysis and usefulness of findings. This section aims to provide a synopsis of the quality of the process evaluations used in this synthesis.

The quality of these process evaluations varies across sectors, where they were published and by whom. None of the process evaluations are linked to an effectiveness study in this review; this creates issues in assessment as they may not follow the pattern of a RCT or quasi‐experimental study. However, in the context of assessing how these evaluations effect the implementation of access programmes, all provide relevance in recommendations for future accommodation programmes.

Six process evaluations presented clear research questions that the programme sought to explore, while two others presented a series of aims that they wanted to achieve during the programme; two process evaluations did not present any research questions or hypothesis; this may be a result of the succinct nature of the reports and their intended audiences.

Only four of the selected evaluations discussed a recognised qualitative research methodology, such as phenomenology or the use of case studies. However, most of the implementation studies did describe some data collection methods such as semi structured interviews, survey data and focus groups with study participants, staff and stakeholders. The methods reflect the researchers desire to collect and collate rich data from service users and staff implementers about factors that influenced the accommodation programme. It is this data that can facilitate in the development of future programmes targeting homeless populations for implementers as it uncovers what works and why.

The process of recruitment was discussed fully in only one of the evaluations, presenting full eligibility criteria. Others discussed this partly or not at all in some cases. Although all evaluations were focused on factors influencing the accessibility of accommodation, some evaluations were clear that their intake and referrals included users who were not homeless (but may have been previously). Although these users were generally separated in any results presented, this does reflect the wide scope of service users that many of these organisations need to accommodate. Another issue is that only four of the studies discussed ethical considerations in any detail, however this is expected as many of the evaluations are not based on trials. The others do not provide sufficient detail regarding ethical considerations or do not report these at all.

None of the interventions have a control group to compare outcomes against. Some evaluations discuss the ethical issues of a control group with no access to accommodation and the implications of this.

In three of the evaluations, a data analysis approach was fully described and with an approach that seemed systematic and sufficiently rigorous, therefore presenting a lower ROB. The other seven evaluations did not describe a rigorous and systematic analysis.

However, all of the selected evaluations present a clear list of recommendations that were based upon the evidence collected and collated in their separate programmes. These recommendations presented valuable insights into what worked, what did not and why for managers, staff and service users implementing and availing of the accommodation programme. It is these insights that are presented as implementation factors within levels of influence in this report and will be useful to implementers of homelessness programmes in the future.

##### Contextual factors

The framework that this synthesis was initially aligned with describes contextual factors as those involving housing and labour markets, however, these themes essentially point towards one issue: access to sufficient and suitable housing. Within this theme, four key topics emerge: social welfare, supply, prejudice, and conditionality.

###### Supply of affordable housing

Access to a sufficient supply of affordable housing emerges as a factor determining the effectiveness of accommodation‐based interventions. For example, Lawlor and Bowen (2017) describes how the Limerick Youth Housing project was able to expand into other areas of Ireland such as North Tipperary, Cork, Clare and Waterford. Despite this expansion of the project, the continued severe housing shortage caused prolonged challenges for project staff. Greenwood (2005) reported the same issues on the availability of housing in Dublin. Government funding restrictions on social housing meant that any property acquired could not be used as transitional housing (Lawlor & Bowen, 2017) and even when exceptions to this were made (e.g., in Limerick) it remained very difficult to purchase properties in a timely manner. Similarly, Busch‐Geertsema (2013) suggested that long wait times for users looking for accommodation, particularly in the case of scattered social housing, was a considerable challenge. Agencies such as the Y‐Foundation in Finland had some success gaining access to housing through the private rented or owner‐occupied sector for use in Housing First programmes. However, by the end of the evaluation, some participants were still waiting on scattered site accommodation, particularly in Scotland, which led to negative experiences for participants.

Keast et al. (2008) cites an innovative response to the accommodation crisis in Australia. One project purchased a motel and refurbished it for social housing purposes into individual self‐contained units. This not only increased capacity and resources on a tight rental housing market but was also highly appropriate for delivering services to recipients. However, it is important to recognise that although solutions and interventions like this may address supply issues for accommodation, they may not be desirable to service users both in terms of the quality of the renovation and the location of the property. This can present difficulties for staff implementing the intervention as although the accommodation and location maybe suitable for staff, it may be less suitable for service users and therefore cause feelings of resentment in the latter group.

Busch‐Geertsema (2013) explores some of these issues in the Housing First Europe evaluation. In this evaluation, it was suggested that dissatisfaction from participants, which was rare overall, related in some cases to the support provided (asking for more support, e.g., in Lisbon), but more often to the choice of housing and in some cases long‐waiting times before being allocated permanent housing. Such problems reflected structural issues, such as a shortage of (affordable and accessible) housing of good quality in preferred locations. Relating to the previous example of refurbishing motels (Keast et al., 2008), implementers of housing interventions need to consider not only the housing context of where the intervention is taking place but also the individual needs of each service user in order to make their journey out of homelessness as comfortable as possible.

###### Social welfare

Social welfare is a key issue that impacts upon homelessness. Due to the different approaches and implementation taken by different jurisdictions across the globe, it is difficult to hypothesise how the same intervention will work in these varying contexts. This is indicated in the Housing First Europe study. Busch‐Geertsema (2013) reports that there is no or almost no housing allowance available in Hungary. Comparably, Pleace and Bretherton (2013) found that restrictions such as “caps” limited the amount of rent that could be paid to someone who was eligible for housing benefits in the UK. The difference in approach towards social welfare can greatly benefit or inhibit an intervention such as Housing First, depending on the context.

Lawlor and Bowen (2017) supports young people who are in receipt of different levels of financial support depending on their circumstances (ranging from €100 to €188) as part of the Focus Ireland project. Staff felt that the amount of financial assistance affected the range of move‐on options available for young people who were only in receipt of reduced‐rate social welfare face particular barriers. Those on lower payments struggled more with budgeting and often owed money at the end of the week, while those on the higher payments felt that the amount was sufficient to live on. The stated policy objective of the reduction in welfare rates for young people was to increase the incentive to take up work. However, homelessness or housing insecurity can often present a much more fundamental barrier to training or employment. Focus Ireland have recommended that young people who are homeless and are engaged in a supported pathway out of homelessness should receive the full adult welfare rate, with labour market supports integrated into the support programme. The participation of the Department of Social Protection in the local partnerships would create an effective mechanism where existing discretion in this regard could be exercised without creating any unintended incentives. On another note, Busch‐Geertsema (2013) in the Housing First Europe evaluation further suggested that given the lack of any sustainable welfare provision to cover the costs of living and housing (except for those who could receive an old age or disability pension), it was essential for participants (in Budapest) to find a job, however most of them had only a very low level of education and no formal qualifications.

###### Prejudice and stigma

In locations where access to housing was not so much of a problem (e.g., Amsterdam, Lisbon, Budapest) participants encountered strong prejudice against them for being homeless, particularly those from the Roma community. Austin et al. (2014) and Sewel (2016) report comparable issues in accessing housing for males, military veterans, ex‐prisoners and care experienced young people, particularly in the variability of the rental market, for example, in the availability, affordability, desirability and safety of housing. The competition for housing can be so extreme that apartments are often rented within 1 h of public advertisement (Austin et al., 2014). Pleace and Bretherton (2013) also reported a lack of affordable housing in the Camden area of London for those who relied on low wages or small welfare packages. They suggested that private landlords were often reluctant to let to people reliant on welfare benefits to pay their rent and living costs, particularly when there was a market for employed people. Pleace and Bretherton (2013) also recorded that a stigma was still attached people with a history of homelessness, particularly those with drug use issues, mental health problems and/or a history of crime.

###### Conditionality

In other cities across Europe, Busch‐Geertsema (2013) and Burt (2009) report that the conditionality placed upon the welfare structure can become a barrier to people who are homeless seeking benefits. For example, in Lisbon, the minimum social income is only paid to people in need if they sign and comply with an inclusion contract and are enroled in the job centre in their neighbourhood. Similarly, in Budapest the conditions for receiving the minimum social income are comparable. Since 2012, service users have either to work or volunteer for 30 days during the year to be eligible for the basic benefit. This can become a barrier to how effective interventions can be in helping service users access housing and sustain enough income to support their accommodation. In essence, if a service user is excluded from receiving benefits due to the conditions that are placed upon them, they will not be able to sustain their accommodation.

##### Policy makers and funders

Policy makers and funders are key stakeholders in tackling homelessness. In this section, three key themes are explored: collaborative approaches, community engagement, and sustainable funding. Each theme can influence how well an intervention is implemented. This is discussed using examples in different jurisdictions and targeting various subgroups of homeless populations.

###### Collaborative approaches

Successful collaboration between stakeholders, agencies and the local community can be a key factor in the implementation of an intervention. For an intervention to have positive outcomes, there should be a shared commitment between policy makers, practitioners and funders to develop interest in intervention projects and create a culture of community buy in.

###### Shared commitment between policy makers, practitioners and funders

A recurring theme that emerges from many of the included studies is the importance of a shared commitment and vision between policy makers, practitioners and funders. This is illustrated in Turner Research and Strategy (2015), whereby the joint vision between funders SHFC (Safe Haven Foundation of Canada) and founders of the programme has created a long‐term relationship that works to support the growing community in their programme. Importantly, both the funders and the founders were extremely active in engaging with community building activities, particularly with programme graduates and current residents. Much of this is orchestrated through community funding for the programme and development of a recreation community fund used for both programme residents and graduates. Often, this affords graduates of the programme to help organise events, reconnect with others and build new relationships, therefore sustaining much of the social and emotional learning gained through the programme. Keast et al. (2008) also reports that considerable effort was invested by new services in brokering relationships with other long‐standing members of the Homelessness Service System. This took the form of significant attendance and involvement at Case Management Group meetings through which the new services were explained in detail and on‐going attempts were made to build relationships.

Keast et al. (2008) indicated that social services at times caused issues due to a reactive rather than proactive approach being taken. A lack of time in terms of users only being admitted for a short time, late referrals, unplanned discharge, discharge from emergency departments and transfer between hospitals can result in a loss of data and lack of support. HRDC, 2003 recommends creating longer time frames to allow for capacity building, consultation, planning and implementation of projects.

###### Coordinating different agencies

In Canada, another issue HRDC (2003) reported was that limited integration between federal departments and agencies into the homelessness initiative was damaging. The federal Minister charged with tackling homelessness lacked a formal mechanism to encourage broader departmental commitment to the homeless initiative. HRDC therefore recommends a more cohesive pan‐federal approach with greater direction, ensuring programmes and policies are informed by both government and community strategies. Keast et al. (2008) report that service implementers further supported coordination in their project by consolidating agreement between partner agencies through Memorandums of Understanding, thereby formalising the relationship and providing a basic framework to guide the shift from single agency working to a collective approach. However, co‐ordinating these agencies in a fashion that both works for the intervention and target group can present challenges. Differing processes, views and previous experiences can all impact upon the implementation of the intervention. Co‐ordinating these ideas into succinct actions between different agencies is key to successful collaboration.

###### Integrating services

Often when an accommodation intervention is implemented, it is paired with a number of different services, such as employment, healthcare or social welfare. These services are usually integrated into an intervention although this integration can be difficult to achieve. A barrier to integrated service provision, identified by Keast et al. (2008), was a sense of inequity in the varying levels of funding and employment conditions between new and established services. It was perceived that some government agencies were more successful in attracting funding than the nongovernment sector. It was recognised however, that without government involvement through agencies, there would be insufficient influence to make any substantial changes.

Where necessary, Pleace and Bretherton (2013) reported that support staff helped families to access support services and made referrals to other agencies when possible, creating a multi‐agency community for service users to engage with. Staff have suggested that the success of the intervention and being able to secure accommodation for users was a result of the relationships that they had built with estate agents. This approach is exemplified where two users explained that Shelter had taken such a lead in multiagency cases that social services had stopped working with the couple because they were being so well supported by other agencies. Keast et al. (2008) suggests that one way to facilitate this is to implement key integration mechanisms to create relationships that can help to encourage communication and engagement with other agencies. For example, in some cases, early intervention workers were often not anchored to their employing agency creating isolation for the workers and exacerbating poor outcomes for service users at the point of crisis. This could be remedied through regular meetings, building good working relationships through manageable contact routes and developing a culture of keeping up to date with other team members (Keast et al., 2008; Sewel, 2016).

##### Community engagement

Wider public and community engagement has also been identified as a key component of successful programme implementation. In their 2014 study, Austin et al. (2014) reports that while leaders in the Veterans Affairs facility were interested in the Housing First programme, they had little understanding of the challenging pragmatics associated with setting up a Housing First intervention. However, without the publicly visible commitment of facility leaders to tackling veteran homelessness the programme would have faced many more issues, particularly in the interface between Veteran Affairs and other agencies both at government and community levels. Human Resources Development Canada (HRDC, 2003) go further, reporting that one of the key success factors was that through their *Supporting Communities Partnership Initiative* model, devolving control of funding to a community level, with appropriate accountability safeguards in place, enables communities to mobilise together to address homelessness in their local area. This in turn can result in a significant increase in partnerships, planning and decision making. Paired with the flexible nature of the terms and conditions of the funding and requirements, this can create a culture of investment, therefore increasing community capacity. One way to mitigate this issue is illustrated in the HRDC evaluation where it was reported that because there were specific officers assigned to each of the project components, they worked closely together with the majority of staff and communities, building solid relationships and creating a culture of trust. This approach can increase buy in from managers and additional collaboration between staff and agencies. However, it should be noted that devolving funding to communities can cause problems in terms of intervention fidelity. Approaches such as this should be given careful consideration before they are embedded into a programme.

In another example, Lawlor and Bowen (2017) acknowledged that a key finding of the evaluation was the clear partnership between Limerick County Council, the agency Tusla and Focus Ireland in delivering the programme which garnered operational activity (allocations meetings every 6 weeks) and an organisational commitment to communication and problem solving. At the time of writing, the report suggested that this partnership approach was a unique working arrangement in the county; Lawlor and Bowen (2017) proposes that greater success could be achieved if this were adopted across the island of Ireland. However, Lawlor and Bowen (2017) suggests that this model requires interagency trust and understanding which should be established through joint staff meetings to encourage transparency. As Keast et al. (2008) observe, while a strong, coherent and meaningful policy framework, formulated on cross sector dialogue can facilitate greater cohesion of resources and effort (potentially resulting in more sustained tenancy and better relationships across organisations), it does not solve the problem of an under resourced service system.

##### Sustainable funding

The amount of funding available, the time constraints often placed on the funding (in terms of applying for it and spending it) as well as competing services and needs, all impact on the support and delivery of accommodation‐based programmes. For example, Busch‐Geertsema (2013) reported that in their Housing First project, overall funding was severally reduced across Europe by around 45% in comparison to the previous year. This had implications on rent subsidies, where lower rents had to be renegotiated with landlords and higher subsidies procured from elsewhere. The number of service users had to be decreased from 60 to 50 and staff capacity had to be reduced from six to four. Additionally, Busch‐Geertsema (2013) suggested that given the lack of any sustainable welfare provision to cover the costs of living and housing (except for those who could receive an old age or disability pension), it was essential for participants (in Budapest) to find a job, however most of them had only a very low level of education and no formal qualifications.

Sustainable funding is a key and ongoing issue for those implementing accommodation interventions. A commitment from government and other stakeholders to provide this sustained funding over several years is essential having a continued service. In relation to funding, Burt (2009) recommends that where possible, resources should be streamlined into one funding package from different government departments. This is evident in Keast et al. (2008) where three government departments (the Department of Housing, Department of Communities and Department of Justice & Attorney‐General) were involved in providing funding for the Responding to Homelessness Strategy. If this fully integrated approach to funding were implemented, it could create capacity to provide both sufficient accommodation and services for individuals experiencing homelessness. However, HRDC (2003) point to the fact that “urgent” funding needs are often in conflict with a planned, consultative approach, particularly for communities at an earlier stage in dealing with homelessness. Keast agrees, reporting that programmes driven by immediacy compounded with complex participant needs and housing shortages create an unsustainable model, particularly for funders to consider.

##### Programme administrators, managers and implementing agencies

People and agencies implementing an intervention hold much of the responsibility in ensuring successful outcomes for service users. The managers often lead on projects, set the tone for the culture that ultimately shapes the programme pathway and take responsibility for prioritising key targets and the resources needed for the intervention to work well. They also draw the map on how service users are referred and access the service in a timely and well managed fashion. The section below takes examples from previous interventions on some factors that have influenced why some aspects of interventions have worked well while others have not.

###### Buy‐in (Leadership, culture, priorities)

Across the process evaluations synthesised in this review, it appears that gaining buy in from programme leadership, managers and agencies involved is an important factor in the success of implementing the accommodation‐based programme. This can be illustrated in the succinct coordination of services and agencies.

Lawlor and Bowen (2017) and Pleace and Bretherton (2013) observe that achieving buy‐in from managers and agencies implementing the intervention can be difficult to manage as it takes time to build new relationships with staff and stakeholders; Burt (2009) acknowledges that this was often the first barrier arising from bringing new organisations with divergent views and approaches together. When organisations with different views do collaborate, there is an acknowledgement that there needs to be a considered route in defining target groups and how best to support them into sustainable accommodation. At the beginning of this process, there are usually discrepancies in the level of commitment and buy in to the cause in the collaborating organisations that needs to be addressed before any meaningful work can begin.

Similarly, Keast et al. (2008) reported that within the “Responding to Homelessness” Strategy, the appointment of a government employee as a “public spaces” co‐ordinator provided agencies with a more succinct way to communicate together, particularly in this case with reference to public intoxication. This helped shift the perception of the issue on the ground from being of a legal nature to one that was more grounded in health and social care frameworks. However, HRDC (2003) and Turner Research (2015) do report that although there was a wide and active representation from all stakeholders in most steering groups, there was some underrepresentation of some communities (in their case, the aboriginal community, LGBTQ+ and newcomer youth). It was noted that further representation from these groups in particular would increase the cultural diversity of the programme, allowing the approach to evolve more and engage more young people facing homelessness from vulnerable backgrounds.

Keast et al. (2008) suggests that the most important enabler of the “Responding to Homelessness Strategy,” is the very apparent sense of good will and commitment to make a real difference to homelessness. This phenomenon crossed government and nongovernment sectors and created good, sustained working relations. Moreover, these relationships and a commitment to service enhancement can serve to smooth over rising tensions between the sectors and maintain focus on the work at hand. The funding that was actioned as a result of this commitment, increased the number and quality of service available to those in need of them.

On another note, although buy in and priorities from leadership is needed, Busch‐Geertsema (2013) suggested that expectations of policy makers and service providers need to remain realistic. For some policy makers, the end goal is in fact full social inclusion. Although ending homelessness provides a platform for further steps towards social inclusion, it is not a guarantee and for the most marginalised individuals relative integration might often be a more realistic goal. Clear communication to policy makers is key in what can be achieved through an accommodation‐based intervention.

###### Identification of recipient/targeting mechanism/referral route (e.g., defined agency or contact)

How a programme or intervention identifies its target group and the pathway put in place to enable this are important. Referral routes provide a clear map to other agencies and contact personnel on who to contact to enable a vulnerable person to have the best level of care. It is important for organisations to think carefully about how to make this pathway clear to community partners and where (physically, structurally) is the most sensible place for a vulnerable person to first make contact with an agency or service. Lawlor and Bowen (2017) and Turner Research (2015) explains that having strong relationships between referrers, such as council staff members, and the service generates a referral route that is accountable for outcomes and decisions made. Regular review meetings create a regular communication pathway between the council and key workers, ensuring that issues are flagged early. However, referrals should be timely, before a household was at crisis point, except of course if this is the intended service design, for example, Housing First. Additionally, for some households there was a reluctance to be referred to another mainstream agency. This created a threat to longer term sustainability for households at risk of homelessness.

###### Sufficiency/adequacy of resources (space, time, staff, budget, appropriateness of services or facilities)

As with many interventions, sufficiency and adequacy of resources has a significant impact on the effectiveness of accommodation‐based interventions (Turner Research, 2015). Programme staff working on a smaller project indicated that its size was an advantage as more time was allowed for one‐to‐one intensive support, tailored for each person. However, this could present issues in maintaining high levels of liaison. This was the case for Barnardo's (Sewel, 2016), matching care leavers to suitable accommodation with providers at near capacity.

The location of staff and services to the accommodation programme was a key factor for implementation for some of the interventions analysed. For example, accessing accommodation was easier for services that had a self‐contained residential service. This clustering of services was demonstrated by by Keast et al. (2008) and users in particular found this beneficial as it provides stability to those with complex and comorbid needs. Similarly, Austin et al. (2014) found that there was wide variation in the size of the catchment areas for which VA facilities in their study were responsible. Some included urban city centres and distant rural communities in catchment areas that cover hundreds of square miles, necessitating specialised knowledge of housing geography as well as time and resources for travel. Staff addressed this issue by permanently stationing teams or individuals ensuring adequate support was available to those living within the catchment area.

##### Staff and case workers

Staff and case workers are often the key implementers of accommodation interventions and therefore play an essential role in ensuring that the intervention works for participants; their commitment to the programme is important to gain in order for it to be implemented correctly. They often have the most contact with participants and are well placed to engage with them and other implementing agencies. It is important for implementing agencies and managers to engage with staff and workers before a new programme is rolled out in order to have the best chance of achieving their buy in. It is of equal importance that staff are given the time and space to actively engage with service users and to receive quality training that will enable skills and attributes that many will have already developed.

###### Buy‐in (commitment to programme)

As with many programmes and interventions, staff on the ground working with people who are experiencing or are at risk of homelessness are key to the success of an intervention (Lawlor & Bowen, 2017). Lawlor and Bowen (2017) also acknowledges that achieving buy‐in from staff around the roll out of a new initiative can be difficult to manage. However, simple initiatives such as developing an induction pack for staff could be used alongside staff training. Once staff were engaged with the programme, Turner Research (2015) recognised that they exhibited a high level of commitment, often going “above and beyond” their formal job expectations. That being said, some staff expressed concern regarding the bureaucracy within the project and the need to meet various accreditation and funding contract requirements.

Staff qualities are an important factor in building rapport with participants and engaging with the community around them. For many experiencing homelessness, staff and case workers are the first point of contact. HRDC (2003) found that many managers and individual staff were frequently described by community members as extremely enthusiastic, energetic and creative. Similarly, Mackie and colleagues (2017) provide a number of individual stories of staff working well together and pushing the limits of their work remits to make sure that homeless individuals with significant needs were able to access the best support. In this example, staff exhibited qualities that were aligned to the overarching philosophy and vision of the programme, in their flexibility, knowledge, understanding and ability to empower homeless individuals. Sewel (2016) reports that when staff were trusting, nonjudgemental, respectful, compassionate and responsive, their users felt valued.

###### Communication and engagement with programme recipients

Lawlor and Bowen (2017) suggests that establishing communication pathways between staff and participants is important. In this Limerick study, frontline staff were given the opportunity to establish and develop good inter‐organisational working relationships which promoted a culture of mutual support from colleagues. Turner Research (2015) noted that staff visited each participant with a frequency that was tailored to individual participant needs and goals. Similarly, Busch‐Geertsema (2013) reported that across the project in Europe, the overwhelming majority of service users were positive about the support they received, how it was provided and the accessibility of staff. Participants felt comfortable with the “no judgement” Housing First approach from staff; they felt that the support provided met their needs and the basic ingredients of the Housing First approach led to high rates of satisfaction from the users. Pleace and Bretherton (2013) suggest that this good working relationship stems from clear and careful management between service staff and users. They admit that this does not preclude arising issues and challenges but does provide clear expectations for what issues staff could address and what other services users could be referred to.

###### Technical skills (capabilities, training)

Although the skills and capabilities of staff working with accommodation services are often viewed in a positive light, Burt (2009) and Lawlor and Bowen (2017) identified that gaps within the training and agency knowledge—including documenting activities and outcomes, harm reduction, suicide intervention, mental health, indigenous and LGBTQ2S+ cultural awareness training—would all be beneficial to the improvement of practice. Staff often did not have the appropriate expertise or qualifications for working with homeless people (Busch Geertsema, 2013). This was the case in Budapest where the teams were not sufficient to organise a successful harm reduction approach for most of the participants in need.

For young people and adults who are service users, the emotional awareness of staff delivering services is often touched upon in evaluation data. Sewel (2001) and Lawlor and Bowen (2017) report from young people that the friendly and nonjudgemental nature of staff empowered participants and made them feel that improving their circumstances was achievable and worthwhile. The young participants surveyed reported that they felt satisfied and supported by their key workers. With a similar participant cohort, Turner Research (2015) recommended providing live‐in support on the accommodation site to provide a positive role modelling for young people. They suggested this created a low turnover rate and helped to mitigate attachment issues. Similar positive remarks were noted in evaluations by Pleace and Bretherton (2013) and Busch‐Geertsema (2013) related to the Housing First approach.

##### Recipients of the programme

A focussed consideration of the recipients of accommodation‐based interventions is central to understanding effectiveness of interventions. Recipients may have particular responsibilities to ensuring success, such as a commitment to engage with the processes of an intervention or adherence to a conditionality advised by implementers. Recipients also have the right to expect good communication from project staff. They might also have particular needs that could be addressed prior to commencement of the programme, such as trust‐building, peer support, flexibility around conditions, or additional and individualised support for illness.

###### Buy‐in (emotional acceptance of programme)

Keast et al. (2008) were aware that promoting a programme to vulnerable people takes time, trust and patience in order to establish a strong relationship. In their studies of young people leaving the care system, Sewel (2016) and Turner Research (2015) recommend that each young person is encouraged to be involved in the decision‐making process right from the beginning of their engagement with the service. This can be facilitated in simple ways, for example, allowing young people to decorate their own rooms, have an active role in determining house rules and having the presence of physical items from their homes. If this is not achieved, placements can breakdown, no matter how much support a young person receives. Programmes such as Haven's Way (Turner Research, 2015) have taken advantage of their growing group of alumnae as a resource when participants need peer support. This strategy builds on the spirit of “giving back” for former participants by encouraging volunteering, peer mentorship, and some fundraising but also increases a sense of acceptance to the programme by participants.

###### Support over time and user independence

When developing a programme, it is important that enough time is built in to support service users sufficiently but that there is also an acknowledgement from the service user that one day they will no longer need the staff or service. Therefore, it is in the interest of staff to foster a sense of service user independence during the programme. Barnardos staff (Sewel, 2016) provided support for participants and independent living by helping young people to find potential properties online, advising on different areas to live, helping them to apply to council housing lists and helping them to physically move to their new home. Similarly, within the Turner Research (2015) evaluation, transition into secure housing plans were developed over lengthy periods as young people took steps to prepare for move‐out. Support was provided with housing location at exit, acquiring necessary basic items and furniture, savings planning and budgeting, as well as building community supports in the new context. One staff member commented there is a “constant dance between handholding and supporting them to do it on their own” reflecting on the issue that agencies and workers ultimately strive for service users to eventually withdraw from the programme because they no longer need support. However, finding the right time and circumstances for this can be lengthy and capacity intensive; it requires service users to gain confidence and trust both in themselves and the staff working with them.

Busch‐Geertsema (2013) recommends that Housing First programmes should carefully consider how to deal with nuisance and neighbourhood conflicts and should put in place clear agreements with both service users and landlords. This evaluation showed that successful management of such problems (if they occur at all) is possible in most cases.

###### Adequacy of information provided and ongoing communication

The process of ensuring both parties understand and sign‐up to accommodation placement agreements and ground rules is facilitated by Barnardo's project workers (Sewel, 2016). The procedure is thorough, covering crucial aspects of placements which need to be clear from the outset. This was perceived to help prevent problems occurring at a later date. By identifying arrangements for resolving any difficulties or disagreements that might arise during the course of the placement, the process also reassures providers. Agreeing ground rules involves negotiation and compromise. Providers and service staff reported that, for young people particularly, this process was very positive, establishing trust between providers and young people early on.

The availability of free calls to users and vice versa can provide users with a safe connection to immediate help and services (Keast et al., 2008). Busch‐Geertsema (2013) reported that satisfaction with the support provided by the NGO operating the Housing First project in Amsterdam was viewed very positively. The majority of interviewees (between 87 and 97%) agreed that:

- They could reach support workers most of the time or always, if necessary;
- They received the information they needed most of the time or always;
- They received information at the right moment;
- Support workers explained things in an understandable way.

###### Accessibility (time and place)

Regarding accessibility for participants, Keast et al. (2008) reported that services located adjacent to public transport were advantageous and considered to have contributed to a high number of presenting users. An important and novel feature of the service is that all calls to the service were free. This initiative was viewed as valuable to both users and to agencies. Users were able to make unconstrained contact including by mobile phone (which has been identified as a prominent mode of communication for users) thus enhancing the immediacy of response and the potential safety of callers. Service agencies have commented that the free service enables contact to be retained with users as they will often call and leave contact details for agency follow‐up.

## 6 DISCUSSION

Building on the gaps identified within previous reviews of accommodation‐based approaches, the present research provides an overview of the effectiveness of 51 articles from 28 studies of accommodation‐based interventions. Twenty‐five out of the 28 studies are from the United States, two from Canada and one from the UK.

We also summarise the qualitative evidence provided by 10 process evaluations. Using a best fit synthesis framework, we identify the key theme: access to sufficient and suitable housing. Within this theme, four key topics emerge: social welfare, supply, prejudice, and conditionality. One of the selected studies are based on interventions conducted in the UK, two in Ireland, one in Australia, one across Europe and the remaining five were carried out in North America; three in the United States and two in Canada

The review team had to create a typology to allow functional and useful comparison between the various intervention types. This requirement was due to the diverse nature of the literature on accommodation‐based approaches, the number of interventions which now exist, inconsistent descriptions of interventions and their elements (e.g., different models of housing, support services, expectations of engagement, etc.), the way in which the approaches are implemented in practice, and country by country differences in terminology had rendered previous categorisations meaningless. These also mask considerable heterogeneity between interventions that are often called the same.

For example, a “shelter” in the United States could not be directly comparable to a shelter in the UK, due to important differences in the services and support offered to individuals. Therefore, by closely examining the components of accommodation‐based approaches through textual analysis of the type of housing offered, the level of support offered, and behavioural conditions placed on the user it is possible to create homogenous categories of interventions. This exploration through a novel typology will contribute o the discourse of researchers in the field by allowing intervention developers to determine which category their intervention fits with best. This categorisation is summarised in Table 1, which we reproduce here for the reader's convenience.

This typology describes interventions from a basic accommodation that only offers relief to basic human needs such as a bed and food often including behavioural expectations (Basic/Conditional category) all the way to longer‐term housing that is offered along assertive, individualised support services with no behavioural conditions attached (High/Unconditional category). While this categorisation does not capture every element of accommodation‐based approaches, it does provide a comparative international framework from which policy makers and funders can work to understand the effectiveness of different accommodation‐based interventions.

Through the findings of the meta‐analysis, readers can identify which categories outlined in the typology are most effective. Through exploratory moderator analyses, it would also be possible to explore who they work best for, in which circumstances, and how they could be improved.

A systematic review was employed to allow synthesis of the available data. Through the rigorous methodological approaches employed, the review provides a robust evidence base outlining the importance and effectiveness of accommodation‐based approaches and details of the characteristics which influence their efficacy. Systematic reviews such as this one are valuable because they assess “bodies of evidence” instead of a single study. By synthesising the results of multiple studies, the findings of systematic reviews are more robust because they are less prone to biases or specific conditions that might be skew the results in any single study.

### 6.1 Summary of main results

Twenty‐eight studies containing 51 accommodation‐based intervention papers were identified from CHI's effectiveness EGM and included in this review of the effectiveness of accommodation‐based programmes for improving outcomes for individuals experiencing, or at risk of experiencing homelessness. Twenty‐five out of 28 of these studies are from the United States, two from Canada and one from the UK. Table 3 highlights that some intervention categories have been more thoroughly investigated than others. While the evidence base is relatively extensive for interventions in the High/Unconditional category (which includes, for instance Housing First), other categories in the lower end of support did not include many studies, or even no studies at all (e.g., Housing only/Conditional).

This study is also novel as it used a network meta‐analysis approach. This approach is very helpful because there are some categories in our typology that have been more investigated than others (e.g., High support/unconditional such as Housing First have been studied aplenty), while some direct comparisons have not been studied in previous research (e.g., there are no direct comparison between Basic/Conditional vs. Basic/Unconditional). By using a network meta‐analysis approach, we are able to make indirect comparisons (e.g., Basic/Conditional vs. Basic/Unconditional) on the basis of other direct comparisons that had been studied (e.g., Basic/Conditional vs. High/Unconditional and Basic/Unconditional vs. High/Unconditional).

The main outcomes of interest for this review were housing stability and health outcomes, which are the areas where the evidence base on accommodation‐based interventions is more extensive. However, not all the studies included all the outcomes we expected to measure. Thus, not all studies were included in each of the meta‐analyses. The evidence from this review indicates that certain models of accommodation‐based programmes can be effective in improving housing stability and health outcomes for those who receive them: namely, High/Unconditional.

We are also interested in understanding the impacts of accommodation‐based programmes on other outcome domains such as crime and justice, employment and income, and capabilities and wellbeing. As these outcomes have been studied considerably less than housing and health outcomes, it was not possible to compare between the different types of accommodation using our typology, thus, we narratively explore the available data on these secondary outcomes.

#### 6.1.1 Primary outcome

##### Housing

The primary outcome of interest was housing stability. Network meta‐analysis enabled us to examine head‐to‐head (direct and indirect) comparisons of different categories of intervention, based on the typology developed by the review team. Note that most of these comparisons are indirect, considering the relative impacts of different interventions versus control conditions to gauge their relative effectiveness. Results showed that interventions offering the highest levels of support alongside unconditional accommodation (High/Unconditional) were more effective in improving housing stability compared to basic support alongside unconditional housing (Basic/Unconditional) (ES = 1.10, 95% CI [0.39, 1.82]), and also in comparison to a no‐intervention control group (ES = 0.62, 95%CI [0.19, 1.06]). These results are comparable to a meta‐analysis on Housing First interventions (Baxter et al., 2019) where participants in the Housing First group spend more days stably housed than the control groups (SMD = 1.24; 95% CI 0.86 to 1.62). There were no other significant differences in effectiveness between the typology categories.

Comparing studies in the different categories of the typology also shows some important trends but two caveats are necessary. First, these results should only be considered indicative in nature given the intrinsic limitations of the indirect comparisons being made. Second, given the indirect comparison being made, the resulting confidence intervals are wide, which represent the uncertainty of “good” and “bad” scenarios that are also consistent with the data. With these caveats in place, there are very important trends that were observed.

First, Basic/Unconditional interventions might harm people: the housing stability outcomes are worse than for all other interventions, including control groups who are not receiving an intervention. The relative effect size is negative and favours control; or, in other words, comparing groups receiving Basic/Unconditional interventions against groups who are receiving no intervention at all (control condition), the Basic/Unconditional group have worse housing stability outcomes.

While many studies have demonstrated the existence of more effective interventions than basic/unconditional interventions (Fitzpatrick‐Lewis et al., 2011; Lako et al., 2018), few have quantified any harm relative to doing nothing. This review provides quantitative evidence congruent with qualitative works which have described the harmful effects of hostels (Johnsen et al., 2018; Watts & Blenkinsopp, 2021; Watts et al., 2018). Notable damage to the individual includes the risk of harm from others (Watts & Blenkinsopp, 2021) and the coercion experienced through lack of alternative offerings aside from acceptance of “highly institutionalised, restrictive and intimidating large‐scale hostels or shelters” (Watts et al., 2018; pg.14).

Second, interventions described as High/Unconditional support achieve better outcomes on housing stability than other available categories of intervention, including those classified as High/Conditional which have similar supports and services in place, but with conditions attached.

Third, in most cases any intervention at all (with the exception of Basic/Unconditional) will perform better than no intervention.

#### 6.1.2 Secondary outcomes

##### Health

A second network meta‐analysis was possible on health outcomes. Again, there were only two comparisons that were statistically significant, and both were against a no‐intervention control. In this analysis interventions categorised as offering Moderate/Conditional (ES = 0.36, 95% CI [0.03, 0.69]) and High/Unconditional (ES = 0.22, 95% CI [0.01, 0.43]) were effective in improving health outcomes compared to no intervention. These effects were smaller than those observed for housing stability. In a meta‐analysis on Housing First programmes, health effects were also much smaller than housing stability effects: Baxter et al. (2019) present effect sizes on three health outcomes including emergency room visits (incidence rate ratio (IRR) = 0.63; 95% CI, 0.48–0.82), number of hospitalisations (IRR = 0.76; 95% CI, 0.70–0.83) and time spent hospitalised (standardised mean difference (SMD) = −0.14; 95% CI, −0.41–0.14). They found no clear differences between the Housing First group and the control group on outcomes related to mental health, quality of life and substance use. Thus, these results are consistent with other related evidence syntheses on the topic. Examination of the relative effectiveness of each category of the typology compared to a no intervention control showed very similar effect sizes for those categories at the more intensive end of the typology (0.22–0.27) and smaller effects at the lower, less intensive end of the typology (−0.1–0.1).

Head‐to‐head comparisons of studies which tested health outcomes also showed some important trends, with the same caveats described above regarding the indirect comparisons and the width of the confidence intervals. As with housing stability outcomes, Basic/Unconditional interventions might harm people: their health outcomes were worse than for all other interventions, including control groups who are not receiving any intervention. This trend across both outcomes may have important implications for practice and policy. Additionally, in most cases any intervention at all (with the exception of Basic/Unconditional) performed better than no intervention for health outcomes.

#### 6.1.3 Additional outcomes

We present narrative summaries on outcomes related to crime and justice, employment, capabilities and wellbeing, and cost. Planned meta‐analyses were not possible due to lack of data across the included studies. If we are to fully understand the manifestation and outcomes associated with homelessness, these are additional avenues which should be explored through further research. Only a handful of studies found positive impacts on these other domains, but the evidence base in this regard remains too sparse to be conclusive.

### 6.2 Overall completeness and applicability of evidence

There are a number of studies which did not report useable data. We have contacted these study authors to request if they have the data we need, but as yet we have had no response to our enquiries.

### 6.3 Quality of the evidence

The quality of the evidence was relatively low but varied across the 28 included studies. Sixteen were RCTs and 12 were quasi‐experimental (nonrandomised) studies. Of the 16 RCTs, eight were high ROB, six were moderate and two were low ROB. Of the 12 quasi‐experimental (nonrandomised) studies one was high/critical ROB, five were serious ROB, five were moderate ROB and one was low ROB. Moderator analyses showed that neither study design, nor ROB, had any undue influence on the magnitude of effect sizes. However, as described in the limitations, these analyses should be interpreted with caution.

Twenty‐five out of the 28 studies are from the United States, two from Canada and one from the UK. This highlights that more high‐quality, rigorous evaluation research is required, especially in other countries and regions outside the United States. This is important because of the way in which country contexts can vary. Applying an evidence base generated in the United States to the UK, for example, risks assuming that the effects observed and reported in one country can be easily translated and result in similar effects in another country. This is not an assumption that we can make due to differences in welfare states and the different ways in which interventions are conceived, understood, costed and implemented between countries. Furthermore, we cannot assume that a no‐intervention condition in one country is in any way comparable to a no‐intervention condition in another. The political, social and historical contexts of different countries and regions may well interact with the effectiveness of interventions to produce very different impacts.

### 6.4 Potential biases in the review process

This review should also be interpreted in the context of its limitations. First, this review was not based on searches conducted by the author team—instead, we drew on the two EGM's already commissioned by CHI. These EGMs were conducted according to Campbell Collaboration standards and guidelines and this is a novel endeavour, for a separate author team to use the studies included in an EGM as the sole source of studies for a systematic review. Searches used for this review were conducted in September 2018.

Second, although subgroup analyses are a staple inclusion to many meta‐analyses as they are useful for developing ideas and exploring heterogeneity, moderator analysis are exploratory in nature and should always be interpreted with caution. Additionally, these types of analyses generally have low statistical power owing to missing data in the primary research due to the incomplete reporting of many of the variables of interest (Borenstein et al., 2009). For the smaller meta‐analyses on the categories of interventions, subgroup analyses were restricted considerably due to this issue and robust conclusions from these analyses are constrained,

#### 6.4.1 Summary of implementation and process (qualitative) findings

The analysis of the qualitative data followed the framework provided by the five main analytical categories of factors of influence (described above and reflected in the EGM). Namely: contextual factors, policy makers/funders, programme administrators/managers/implementing agencies, staff/case workers and recipients of the programme.

#### 6.4.2 Contextual factors

The primary issue raised in relation to context was the lack of stable, affordable accommodation and the variability in the rental market, such that actually sourcing accommodation to provide for individuals who are homeless is extremely challenging. Various agencies have tackled this issue, such as the Y Foundation in Finland who gained access to housing through the private rented or owner‐occupied sector for use in Housing First programmes. Similarly, Keast et al. (2008) purchased a motel in Australia and refurbished it for social housing purposes into individual self‐contained units. This increased capacity and resources in an under‐resourced rental housing market.

Notably, social welfare practices also influence the uptake and success of accommodation‐based approaches. Busch‐Geertsema (2013) state that there is no or almost no housing allowance available in Hungary and Pleace and Bretherton (2013) found that restrictions such as “caps” limited the amount of rent that could be paid to someone who was eligible for housing benefits in the UK. As part of the Focus Ireland project, recipients were provided with financial assistance and staff felt that the level of social welfare affected the range of move‐on options available for young people (Lawlor & Bowen, 2017).

Additionally, prejudice against people who are homeless can prevent their access to the private rental market. In locations where access to housing was not so much of a problem (for example Amsterdam, Lisbon, Budapest) participants encountered strong prejudice against them for being homeless, particularly those from the Roma community.

Finally, the conditionality of different countries' welfare structures can act as an additional barrier to accessing housing. In essence, if a service user is excluded from receiving benefits due to the conditions that are placed upon them, they will not be able to sustain their accommodation.

#### 6.4.3 Policy makers and funders

Policy makers and funders are key stakeholders in tackling homelessness. Successful collaboration and shared commitment between stakeholders, agencies and the local community, coordination between different agencies and integration of services are all key factors in successful provision of housing. Successful collaboration is important to foster and develop interest in intervention projects and creating a culture of community buy in. Shared commitment and vision between policy makers, practitioners and funders allows for capacity building, consultation, planning and implementation of projects. Coordination between different agencies leads to a more cohesive pan‐federal approach with greater direction, ensuring programmes and policies are informed by both government and community strategies. Finally, integrating services allow support staff to help families to access support services and made referrals to other agencies when possible, creating a multi‐agency community for service users to engage with. Keast et al. (2008) suggests that one way to facilitate this is to implement key integration mechanisms to create relationships that can help to encourage communication and engagement with other agencies.

Programme administrators, managers and implementing agencies integration and buy‐in was also considered key at these strategic levels of “on the ground” implementation. Forging positive relationships and identifying key “point people” to manage and coordinate inter agency communication was seen as very important. Clarity around referral procedures, early identification and prioritisation of need (in a participatory way) and employing a well‐planned approach was considered an ongoing challenge.

#### 6.4.4 Staff and case workers

Staff and case workers were identified as essential to the success of accommodation‐based interventions. This included both their individual qualities and enthusiasm but also their capacity to liaise with other relevant agencies to provide additional support to users. Staff spending time navigating the complex logistics associate with sourcing and providing suitable accommodation was seen to be at the expense of providing other additional support services. Easy, safe and nonjudgmental communication pathways between staff and participants was considered essential to tailor support to individual needs.

#### 6.4.5 Recipients of the programme

Effective and meaningful engagement with users and where possible, involving individuals in decisions about their housing needs and placement, was considered essential to both the success of the programme and the satisfaction of the user. Accessing additional, appropriate and practical nonhousing support was perceived to be a key enabler. The provision of support to set up home (e.g., furniture, help moving in) as well as ongoing support (e.g., liaising with landlords) was identified as promoting a more sustainable placement.

### 6.5 Agreements and disagreements with other studies or reviews

The primary outcome of interest was housing stability. Network meta‐analysis enabled us to examine head‐to‐head (direct and indirect) comparisons of different categories of intervention, based on the typology developed by the review team. Note that most of these comparisons are indirect, considering the relative impacts of different interventions versus control conditions to gauge their relative effectiveness. Results showed that interventions offering the highest levels of support alongside unconditional accommodation (High/Unconditional) were more effective in improving housing stability compared to basic support alongside unconditional housing (Basic/Unconditional) (ES = 1.10; 95% CI, 0.39–1.82), and also in comparison to a no‐intervention control group (ES = 0.62; 95% CI, 0.19, 1.06). These results are comparable to a meta‐analysis on Housing First interventions (Baxter et al., 2019) where participants in the Housing First group spend more days stably housed than the control groups (SMD = 1.24; 95% CI, 0.86–1.62). There were no other significant differences in effectiveness between the typology categories.

A second network meta‐analysis was possible on health outcomes. Again, there were only two comparisons that were statistically significant, and both were against a no‐intervention control. In this analysis interventions categorised as offering Moderate/Conditional (ES = 0.36; 95% CI, 0.03, 0.69) and High/Unconditional (ES = 0.22; 95% CI, 0.01, 0.43) were effective in improving health outcomes compared to no intervention. These effects were smaller than those observed for housing stability. In a meta‐analysis on Housing First programmes, health effects were also much smaller than housing stability effects: Baxter et al. (2019) present effect sizes on three health outcomes including emergency room visits (incidence rate ratio (IRR) = 0.63; 95% CI, 0.48–0.82), number of hospitalisations (IRR = 0.76; 95% CI, 0.70–0.83) and time spent hospitalised (standardised mean difference (SMD) = −0.14; 95% CI, −0.41–0.14). They found no clear differences between the Housing First group and the control group on outcomes related to mental health, quality of life and substance use. Thus, these results are consistent with other related evidence syntheses on the topic. Examination of the relative effectiveness of each category of the typology compared to a no intervention control showed very similar effect sizes for those categories at the more intensive end of the typology (0.22–0.27) and smaller effects at the lower, less intensive end of the typology (−0.1–0.1).

## 7 AUTHORS' CONCLUSIONS

### 7.1 Implications for practice

Policy makers and practitioners have had a responsibility to protect individuals experiencing or at risk of experiencing homelessness from the debilitating effects of living without a home. Due to these responsibilities, many researchers have now attempted to understand which accommodation‐based interventions may work best, for whom, and in which circumstances. Through this synthesis of the available and most robust research, this review provides a more accurate representation of reality, by combining more data than a primary research study feasibly could.

To investigate how the primary research could (and should) influence policy and practice changes, the researchers incorporated and facilitated all the theoretical presuppositions offered through the large body of empirical research to create a typology based on persistent and recurring descriptions including the type of housing, the level of support, and the behavioural expectations posited on the user. Using a new typology to categorise approaches and through inclusion of qualitative information, this research incorporated and facilitated many theoretical presuppositions offered through the empirical research to offer some suggestions to policy makers and practitioners.

The network meta‐analysis suggests that all types of accommodation which provided support are more effective than no intervention or Basic/Unconditional accommodation in terms of housing stability and health.

Additionally, accommodation with higher levels of support “blended” into the intervention, such as High/Conditional and High/Unconditional (which includes for instance Housing First), had the strongest evidence of effectiveness. However, this does not necessarily mean that interventions in the High Support/Unconditional category are required or would be appropriate for all individuals: substantial uncertainty remains when contrasting the relative effectiveness of interventions in the medium and high levels of support. Beyond housing stability, decision makers can expect accommodation‐based approaches with support (those which go further than the provision of a bed) to have positive outcomes on participants' health.

Those interventions which are described as Basic/Unconditional (i.e., those that only satisfy very basic human needs such as a bed and food) harm people: they had worse health and housing stability outcomes even when compared to no intervention. This invites policy makers and practitioners to question whether these types of accommodation‐based interventions are the best use of limited funds or whether they should be discontinued entirely when other more suitable and effective offers of support can be made available.

While the present quantitative evidence might be promising, most of the evidence summarised in this review was from North America, therefore, policy makers and practitioners outside North America should view this in the context of its limitations. Policy makers and practitioners in the UK, for example, should assess and acknowledge the level of support already available, and implement policy based on what already exists. For example, the social safety nets available in the UK context are more generous than in the US context. As such, some of the impacts observed in the North American context against their usual practices might not be directly translated to other countries where more extensive and generous social services are deployed as usual care.

The qualitative evidence, in turn, has been much more geographically dispersed and therefore provides a much more representative summary of accommodation‐based approaches. This type of evidence tells us that the success of accommodation‐ based approaches depends on many contextual factors including welfare policy, the skills and approach of staff and access to housing for example in the private rental marker. This type of evidence also identifies practical service delivery factors which may impact on the success of accommodation‐based approaches. The following factors were identified as enablers:

- Clear identification of suitable users, referral routes and approaches to prioritisation
- Effective and meaningful engagement with users and involving people in decisions about their housing and support, was a factor in outcomes and user satisfaction
- Many of the features of a person‐centred and holistic approach including flexibility in support work, a nonjudgemental approach and clear communication
- The time and knowledge to assist with navigating systems, like those used to secure accommodation, for example, were also identified as enablers to housing stability
- Collaboration with other agencies. Everyone needs to commit to shared objectives and principles to avoid confusion, misunderstanding and wasting resources
- Ability to recruit and retain quality programme staff. They need robust training and to be able to share in the vision of the intervention. This secures buy in and confidence in their ability to improve outcomes
- Long‐term, sustainable funding. Funding should come from committed sources so that when the intervention ends, service‐users have received the support they require to avoid falling back into homelessness.

Accommodation provision is likely to be an essential, evidence‐based element of overall local plans to alleviate and prevent homelessness. There are a multitude of models that commissioners can draw on. There is evidence that suggests but does not prove, that accommodation interventions with support reduce the costly use of public services due to offending and poor health and may therefore “save” more overall than they cost; in this context local cost benefit modelling may help to make a case for accommodation interventions for people facing homelessness.

### 7.2 Implications for research

As with any meta‐analysis, the quality of the conclusion made have been dependent on the rigour of the primary studies. Through the conduct of this research, some consistent limitations were exposed and there are several potential areas to address through further empirical research.

First, although there was enough research to look at the effectiveness of the various accommodation‐based approaches (as defined by our typology) on housing stability and health outcomes, the other outcomes measured by our review (Crime and justice, Employment, Capabilities and wellbeing, Cost) could not be measured by individual approaches and instead we present only a narrative description for these secondary outcomes. Further research could either focus on the effectiveness of accommodation‐based approaches on less reported outcomes (Crime and justice, Employment, Capabilities and wellbeing, Cost) specifically, or at least add measurements of these outcomes in future research.

Second, half of the research included in this review has been judged to have serious problems (18%) and high, critical (32%) ROB due to issues with their methodology and how the research was conducted. ROB due to lack of blinding is more common in social science research than in other disciples (such as medicine). Reasons for this are due to difficulties in blinding participants and study personnel who are assigned to the intervention group (e.g., it will be obvious that the group who are in receipt of accommodation are likely to be the intervention group). However, it is possible to add methodological rigour to social science research by ensuring that the outcome assessors (e.g., those who analyse the comparisons between groups) are blinded to which group received the intervention.

Third, aside from the importance of conducting relevant and methodologically rigorous research, there are specific gaps related to the geographical context of the research conducted. A large majority of the studies included in this systematic review are from the United States and Canada which have very different social welfare systems to those of the UK, for example. Primary research from those locations which are not currently represented in the literature will bring unique perspectives to the evidence‐base and would ensure they are better adapted to the differences in context and policy environment in each of these areas.

Fourth, it is difficult to draw concise conclusions if primary studies do not routinely report important intervention characteristics (e.g., age and gender of participants, level of support needed to address additional needs, whether housing is scattered or congregate, conditionality placed on the participants etc.). To allow more accurate testing of these important moderating variables, it is also recommended that these characteristics be described separately for intervention and control participants. This will enable more robust conclusions which can be implemented by others with more confidence.

Finally, this current research makes a significant and unique contribution to research through the development and application of a novel typology. Future researchers should clearly define their interventions based on the typology by (1) highlighting the intensity of support; (2) whether conditionality was applied, and (3) the type of housing provided to the participants. This will facilitate understanding of the effectiveness of these three intervention components and allow for more detailed comparison.

## CONTRIBUTIONS OF AUTHORS

Sarah Miller was the principal investigator and manager of this project. Prof. Miller contributed to all aspects of the review. Ciara Keenan was responsible for the day‐to‐day operation of the review, contributed to all aspects of the review process and drafted all reports. Jennifer Hanratty and Christopher Coughlan contributed to screening, extraction, and quality appraisal of included studies. Jayne Hamilton led on all sections related to qualitative evidence synthesis and assisted with screening, extraction, and quality appraisal of studies. Terri Pigott conducted all analyses presented in the review and provided detail on their interpretation. Peter Mackie, Suzanne Fitzpatrick, and John Cowman provided content expertise throughout the review process.

## DECLARATIONS OF INTEREST

### Preliminary timeframe

March 2021.

### PLANS FOR UPDATING THIS REVIEW

Dependant on additional funding.

## DIFFERENCES BETWEEN PROTOCOL AND REVIEW

There was an occasion during the conduct of the review where it was necessary to deviate from the protocol. The review team wanted to understand if implementation fidelity was related to the effectiveness of the intervention. However, this data was not present in the primary studies and therefore this analysis was not possible.

## CHARACTERISTICS OF STUDIES

**Characteristics of included studies**

Appel et al. (2012)

**Table:** 

**Methods**
**Participants**
**Interventions**
**Outcomes**
**Notes**

Risk of bias table

**Table:** 

Bias	Authors' judgement	Support for judgement
Random sequence generation (selection bias)	Unclear risk	
Allocation concealment (selection bias)	Unclear risk	
Blinding of participants and personnel (performance bias)	Unclear risk	
Blinding of outcome assessment (detection bias)	Unclear risk	
Incomplete outcome data (attrition bias)	Unclear risk	
Selective reporting (reporting bias)	Unclear risk	
Other bias	Unclear risk	

Aquin et al. (2017)

**Table:** 

**Methods**
**Participants**
**Interventions**
**Outcomes**
**Notes**

Risk of bias table

**Table:** 

Bias	Authors' judgement	Support for judgement
Random sequence generation (selection bias)	Unclear risk	
Allocation concealment (selection bias)	Unclear risk	
Blinding of participants and personnel (performance bias)	Unclear risk	
Blinding of outcome assessment (detection bias)	Unclear risk	
Incomplete outcome data (attrition bias)	Unclear risk	
Selective reporting (reporting bias)	Unclear risk	
Other bias	Unclear risk	

Aubry et al. (2015)

**Table:** 

**Methods**
**Participants**
**Interventions**
**Outcomes**
**Notes**

Risk of bias table

**Table:** 

Bias	Authors' judgement	Support for judgement
Random sequence generation (selection bias)	Unclear risk	
Allocation concealment (selection bias)	Unclear risk	
Blinding of participants and personnel (performance bias)	Unclear risk	
Blinding of outcome assessment (detection bias)	Unclear risk	
Incomplete outcome data (attrition bias)	Unclear risk	
Selective reporting (reporting bias)	Unclear risk	
Other bias	Unclear risk	

Austin et al. (2014)

**Table:** 

**Methods**
**Participants**
**Interventions**
**Outcomes**
**Notes**

Risk of bias table

**Table:** 

Bias	Authors' judgement	Support for judgement
Random sequence generation (selection bias)	Unclear risk	
Allocation concealment (selection bias)	Unclear risk	
Blinding of participants and personnel (performance bias)	Unclear risk	
Blinding of outcome assessment (detection bias)	Unclear risk	
Incomplete outcome data (attrition bias)	Unclear risk	
Selective reporting (reporting bias)	Unclear risk	
Other bias	Unclear risk	

Basu et al. (2012)

**Table:** 

**Methods**
**Participants**
**Interventions**
**Outcomes**
**Notes**

Risk of bias table

**Table:** 

Bias	Authors' judgement	Support for judgement
Random sequence generation (selection bias)	Unclear risk	
Allocation concealment (selection bias)	Unclear risk	
Blinding of participants and personnel (performance bias)	Unclear risk	
Blinding of outcome assessment (detection bias)	Unclear risk	
Incomplete outcome data (attrition bias)	Unclear risk	
Selective reporting (reporting bias)	Unclear risk	
Other bias	Unclear risk	

Brown et al. (2016)

**Table:** 

**Methods**
**Participants**
**Interventions**
**Outcomes**
**Notes**

Risk of bias table

**Table:** 

Bias	Authors' judgement	Support for judgement
Random sequence generation (selection bias)	Unclear risk	
Allocation concealment (selection bias)	Unclear risk	
Blinding of participants and personnel (performance bias)	Unclear risk	
Blinding of outcome assessment (detection bias)	Unclear risk	
Incomplete outcome data (attrition bias)	Unclear risk	
Selective reporting (reporting bias)	Unclear risk	
Other bias	Unclear risk	

Buchanan et al. (2006)

**Table:** 

**Methods**
**Participants**
**Interventions**
**Outcomes**
**Notes**

Risk of bias table

**Table:** 

Bias	Authors' judgement	Support for judgement
Random sequence generation (selection bias)	Unclear risk	
Allocation concealment (selection bias)	Unclear risk	
Blinding of participants and personnel (performance bias)	Unclear risk	
Blinding of outcome assessment (detection bias)	Unclear risk	
Incomplete outcome data (attrition bias)	Unclear risk	
Selective reporting (reporting bias)	Unclear risk	
Other bias	Unclear risk	

Buchanan et al. (2009)

**Table:** 

**Methods**
**Participants**
**Interventions**
**Outcomes**
**Notes**

Risk of bias table

**Table:** 

Bias	Authors' judgement	Support for judgement
Random sequence generation (selection bias)	Unclear risk	
Allocation concealment (selection bias)	Unclear risk	
Blinding of participants and personnel (performance bias)	Unclear risk	
Blinding of outcome assessment (detection bias)	Unclear risk	
Incomplete outcome data (attrition bias)	Unclear risk	
Selective reporting (reporting bias)	Unclear risk	
Other bias	Unclear risk	

Burt (2009)

**Table:** 

**Methods**
**Participants**
**Interventions**
**Outcomes**
**Notes**

Risk of bias table

**Table:** 

Bias	Authors' judgement	Support for judgement
Random sequence generation (selection bias)	Unclear risk	
Allocation concealment (selection bias)	Unclear risk	
Blinding of participants and personnel (performance bias)	Unclear risk	
Blinding of outcome assessment (detection bias)	Unclear risk	
Incomplete outcome data (attrition bias)	Unclear risk	
Selective reporting (reporting bias)	Unclear risk	
Other bias	Unclear risk	

Busch‐Geertsema (2013)

**Table:** 

**Methods**
**Participants**
**Interventions**
**Outcomes**
**Notes**

Risk of bias table

**Table:** 

Bias	Authors' judgement	Support for judgement
Random sequence generation (selection bias)	Unclear risk	
Allocation concealment (selection bias)	Unclear risk	
Blinding of participants and personnel (performance bias)	Unclear risk	
Blinding of outcome assessment (detection bias)	Unclear risk	
Incomplete outcome data (attrition bias)	Unclear risk	
Selective reporting (reporting bias)	Unclear risk	
Other bias	Unclear risk	

Cheng et al. (2007)

**Table:** 

**Methods**
**Participants**
**Interventions**
**Outcomes**
**Notes**

Risk of bias table

**Table:** 

**Bias**	**Authors' judgement**	**Support for judgement**
Random sequence generation (selection bias)	Unclear risk	
Allocation concealment (selection bias)	Unclear risk	
Blinding of participants and personnel (performance bias)	Unclear risk	
Blinding of outcome assessment (detection bias)	Unclear risk	
Incomplete outcome data (attrition bias)	Unclear risk	
Selective reporting (reporting bias)	Unclear risk	
Other bias	Unclear risk	

Gilmer et al. (2010)

**Table:** 

**Methods**
**Participants**
**Interventions**
**Outcomes**
**Notes**

Risk of bias table

**Table:** 

Bias	Authors' judgement	Support for judgement
Random sequence generation (selection bias)	Unclear risk	
Allocation concealment (selection bias)	Unclear risk	
Blinding of participants and personnel (performance bias)	Unclear risk	
Blinding of outcome assessment (detection bias)	Unclear risk	
Incomplete outcome data (attrition bias)	Unclear risk	
Selective reporting (reporting bias)	Unclear risk	
Other bias	Unclear risk	

Goering et al. (2011)

**Table:** 

**Methods**
**Participants**
**Interventions**
**Outcomes**
**Notes**

Risk of bias table

**Table:** 

Bias	Authors' judgement	Support for judgement
Random sequence generation (selection bias)	Unclear risk	
Allocation concealment (selection bias)	Unclear risk	
Blinding of participants and personnel (performance bias)	Unclear risk	
Blinding of outcome assessment (detection bias)	Unclear risk	
Incomplete outcome data (attrition bias)	Unclear risk	
Selective reporting (reporting bias)	Unclear risk	
Other bias	Unclear risk	

Goering (2012)

**Table:** 

**Methods**
**Participants**
**Interventions**
**Outcomes**
**Notes**

Risk of bias table

**Table:** 

Bias	Authors' judgement	Support for judgement
Random sequence generation (selection bias)	Unclear risk	
Allocation concealment (selection bias)	Unclear risk	
Blinding of participants and personnel (performance bias)	Unclear risk	
Blinding of outcome assessment (detection bias)	Unclear risk	
Incomplete outcome data (attrition bias)	Unclear risk	
Selective reporting (reporting bias)	Unclear risk	
Other bias	Unclear risk	

Goldfinger et al. (1999)

**Table:** 

**Methods**
**Participants**
**Interventions**
**Outcomes**
**Notes**

Risk of bias table

**Table:** 

Bias	Authors' judgement	Support for judgement
Random sequence generation (selection bias)	Unclear risk	
Allocation concealment (selection bias)	Unclear risk	
Blinding of participants and personnel (performance bias)	Unclear risk	
Blinding of outcome assessment (detection bias)	Unclear risk	
Incomplete outcome data (attrition bias)	Unclear risk	
Selective reporting (reporting bias)	Unclear risk	
Other bias	Unclear risk	

Greenwood et al. (2005)

**Table:** 

**Methods**
**Participants**
**Interventions**
**Outcomes**
**Notes**

Risk of bias table

**Table:** 

Bias	Authors' judgement	Support for judgement
Random sequence generation (selection bias)	Unclear risk	
Allocation concealment (selection bias)	Unclear risk	
Blinding of participants and personnel (performance bias)	Unclear risk	
Blinding of outcome assessment (detection bias)	Unclear risk	
Incomplete outcome data (attrition bias)	Unclear risk	
Selective reporting (reporting bias)	Unclear risk	
Other bias	Unclear risk	

Gulcur et al. (2003)

**Table:** 

**Methods**
**Participants**
**Interventions**
**Outcomes**
**Notes**

Risk of bias table

**Table:** 

Bias	Authors' judgement	Support for judgement
Random sequence generation (selection bias)	Unclear risk	
Allocation concealment (selection bias)	Unclear risk	
Blinding of participants and personnel (performance bias)	Unclear risk	
Blinding of outcome assessment (detection bias)	Unclear risk	
Incomplete outcome data (attrition bias)	Unclear risk	
Selective reporting (reporting bias)	Unclear risk	
Other bias	Unclear risk	

Howard et al. (2011)

**Table:** 

**Methods**
**Participants**
**Interventions**
**Outcomes**
**Notes**

Risk of bias table

**Table:** 

Bias	Authors' judgement	Support for judgement
Random sequence generation (selection bias)	Unclear risk	
Allocation concealment (selection bias)	Unclear risk	
Blinding of participants and personnel (performance bias)	Unclear risk	
Blinding of outcome assessment (detection bias)	Unclear risk	
Incomplete outcome data (attrition bias)	Unclear risk	
Selective reporting (reporting bias)	Unclear risk	
Other bias	Unclear risk	

HRDC (2003)

**Table:** 

**Methods**
**Participants**
**Interventions**
**Outcomes**
**Notes**

Risk of bias table

**Table:** 

Bias	Authors' judgement	Support for judgement
Random sequence generation (selection bias)	Unclear risk	
Allocation concealment (selection bias)	Unclear risk	
Blinding of participants and personnel (performance bias)	Unclear risk	
Blinding of outcome assessment (detection bias)	Unclear risk	
Incomplete outcome data (attrition bias)	Unclear risk	
Selective reporting (reporting bias)	Unclear risk	
Other bias	Unclear risk	

Hwang et al. (2011)

**Table:** 

**Methods**
**Participants**
**Interventions**
**Outcomes**
**Notes**

Risk of bias table

**Table:** 

Bias	Authors' judgement	Support for judgement
Random sequence generation (selection bias)	Unclear risk	
Allocation concealment (selection bias)	Unclear risk	
Blinding of participants and personnel (performance bias)	Unclear risk	
Blinding of outcome assessment (detection bias)	Unclear risk	
Incomplete outcome data (attrition bias)	Unclear risk	
Selective reporting (reporting bias)	Unclear risk	
Other bias	Unclear risk	

Keast et al. (2008)

**Table:** 

**Methods**
**Participants**
**Interventions**
**Outcomes**
**Notes**

Risk of bias table

**Table:** 

Bias	Authors' judgement	Support for judgement
Random sequence generation (selection bias)	Unclear risk	
Allocation concealment (selection bias)	Unclear risk	
Blinding of participants and personnel (performance bias)	Unclear risk	
Blinding of outcome assessment (detection bias)	Unclear risk	
Incomplete outcome data (attrition bias)	Unclear risk	
Selective reporting (reporting bias)	Unclear risk	
Other bias	Unclear risk	

Kertesz et al. (2007)

**Table:** 

**Methods**
**Participants**
**Interventions**
**Outcomes**
**Notes**

Risk of bias table

**Table:** 

Bias	Authors' judgement	Support for judgement
Random sequence generation (selection bias)	Unclear risk	
Allocation concealment (selection bias)	Unclear risk	
Blinding of participants and personnel (performance bias)	Unclear risk	
Blinding of outcome assessment (detection bias)	Unclear risk	
Incomplete outcome data (attrition bias)	Unclear risk	
Selective reporting (reporting bias)	Unclear risk	
Other bias	Unclear risk	

Kirst et al. (2015)

**Table:** 

**Methods**
**Participants**
**Interventions**
**Outcomes**
**Notes**

Risk of bias table

**Table:** 

Bias	Authors' judgement	Support for judgement
Random sequence generation (selection bias)	Unclear risk	
Allocation concealment (selection bias)	Unclear risk	
Blinding of participants and personnel (performance bias)	Unclear risk	
Blinding of outcome assessment (detection bias)	Unclear risk	
Incomplete outcome data (attrition bias)	Unclear risk	
Selective reporting (reporting bias)	Unclear risk	
Other bias	Unclear risk	

Kozloff et al. (2016)

**Table:** 

**Methods**
**Participants**
**Interventions**
**Outcomes**
**Notes**

Risk of bias table

**Table:** 

Bias	Authors' judgement	Support for judgement
Random sequence generation (selection bias)	Unclear risk	
Allocation concealment (selection bias)	Unclear risk	
Blinding of participants and personnel (performance bias)	Unclear risk	
Blinding of outcome assessment (detection bias)	Unclear risk	
Incomplete outcome data (attrition bias)	Unclear risk	
Selective reporting (reporting bias)	Unclear risk	
Other bias	Unclear risk	

Larimer et al. (2009)

**Table:** 

**Methods**
**Participants**
**Interventions**
**Outcomes**
**Notes**

Risk of bias table

**Table:** 

Bias	Authors' judgement	Support for judgement
Random sequence generation (selection bias)	Unclear risk	
Allocation concealment (selection bias)	Unclear risk	
Blinding of participants and personnel (performance bias)	Unclear risk	
Blinding of outcome assessment (detection bias)	Unclear risk	
Incomplete outcome data (attrition bias)	Unclear risk	
Selective reporting (reporting bias)	Unclear risk	
Other bias	Unclear risk	

Lawlor and Bowen (2017)

**Table:** 

**Methods**
**Participants**
**Interventions**
**Outcomes**
**Notes**

Risk of bias table

**Table:** 

Bias	Authors' judgement	Support for judgement
Random sequence generation (selection bias)	Unclear risk	
Allocation concealment (selection bias)	Unclear risk	
Blinding of participants and personnel (performance bias)	Unclear risk	
Blinding of outcome assessment (detection bias)	Unclear risk	
Incomplete outcome data (attrition bias)	Unclear risk	
Selective reporting (reporting bias)	Unclear risk	
Other bias	Unclear risk	

Levitt et al. (2013)

**Table:** 

**Methods**
**Participants**
**Interventions**
**Outcomes**
**Notes**

Risk of bias table

**Table:** 

Bias	Authors' judgement	Support for judgement
Random sequence generation (selection bias)	Unclear risk	
Allocation concealment (selection bias)	Unclear risk	
Blinding of participants and personnel (performance bias)	Unclear risk	
Blinding of outcome assessment (detection bias)	Unclear risk	
Incomplete outcome data (attrition bias)	Unclear risk	
Selective reporting (reporting bias)	Unclear risk	
Other bias	Unclear risk	

Lim et al. (2017)

**Table:** 

**Methods**
**Participants**
**Interventions**
**Outcomes**
**Notes**

Risk of bias table

**Table:** 

Bias	Authors' judgement	Support for judgement
Random sequence generation (selection bias)	Unclear risk	
Allocation concealment (selection bias)	Unclear risk	
Blinding of participants and personnel (performance bias)	Unclear risk	
Blinding of outcome assessment (detection bias)	Unclear risk	
Incomplete outcome data (attrition bias)	Unclear risk	
Selective reporting (reporting bias)	Unclear risk	
Other bias	Unclear risk	

Lim (2018)

**Table:** 

**Methods**
**Participants**
**Interventions**
**Outcomes**
**Notes**

Risk of bias table

**Table:** 

Bias	Authors' judgement	Support for judgement
Random sequence generation (selection bias)	Unclear risk	
Allocation concealment (selection bias)	Unclear risk	
Blinding of participants and personnel (performance bias)	Unclear risk	
Blinding of outcome assessment (detection bias)	Unclear risk	
Incomplete outcome data (attrition bias)	Unclear risk	
Selective reporting (reporting bias)	Unclear risk	
Other bias	Unclear risk	

Lipton et al. (2000)

**Table:** 

**Methods**
**Participants**
**Interventions**
**Outcomes**
**Notes**

Risk of bias table

**Table:** 

Bias	Authors' judgement	Support for judgement
Random sequence generation (selection bias)	Unclear risk	
Allocation concealment (selection bias)	Unclear risk	
Blinding of participants and personnel (performance bias)	Unclear risk	
Blinding of outcome assessment (detection bias)	Unclear risk	
Incomplete outcome data (attrition bias)	Unclear risk	
Selective reporting (reporting bias)	Unclear risk	
Other bias	Unclear risk	

McHugo et al. (2004)

**Table:** 

**Methods**
**Participants**
**Interventions**
**Outcomes**
**Notes**

Risk of bias table

**Table:** 

Bias	Authors' judgement	Support for judgement
Random sequence generation (selection bias)	Unclear risk	
Allocation concealment (selection bias)	Unclear risk	
Blinding of participants and personnel (performance bias)	Unclear risk	
Blinding of outcome assessment (detection bias)	Unclear risk	
Incomplete outcome data (attrition bias)	Unclear risk	
Selective reporting (reporting bias)	Unclear risk	
Other bias	Unclear risk	

Mennemeyer et al. (2017)

**Table:** 

**Methods**
**Participants**
**Interventions**
**Outcomes**
**Notes**

Risk of bias table

**Table:** 

Bias	Authors' judgement	Support for judgement
Random sequence generation (selection bias)	Unclear risk	
Allocation concealment (selection bias)	Unclear risk	
Blinding of participants and personnel (performance bias)	Unclear risk	
Blinding of outcome assessment (detection bias)	Unclear risk	
Incomplete outcome data (attrition bias)	Unclear risk	
Selective reporting (reporting bias)	Unclear risk	
Other bias	Unclear risk	

Milby et al. (1996)

**Table:** 

**Methods**
**Participants**
**Interventions**
**Outcomes**
**Notes**

Risk of bias table

**Table:** 

Bias	Authors' judgement	Support for judgement
Random sequence generation (selection bias)	Unclear risk	
Allocation concealment (selection bias)	Unclear risk	
Blinding of participants and personnel (performance bias)	Unclear risk	
Blinding of outcome assessment (detection bias)	Unclear risk	
Incomplete outcome data (attrition bias)	Unclear risk	
Selective reporting (reporting bias)	Unclear risk	
Other bias	Unclear risk	

Milby et al. (2000)

**Table:** 

**Methods**
**Participants**
**Interventions**
**Outcomes**
**Notes**

Risk of bias table

**Table:** 

Bias	Authors' judgement	Support for judgement
Random sequence generation (selection bias)	Unclear risk	
Allocation concealment (selection bias)	Unclear risk	
Blinding of participants and personnel (performance bias)	Unclear risk	
Blinding of outcome assessment (detection bias)	Unclear risk	
Incomplete outcome data (attrition bias)	Unclear risk	
Selective reporting (reporting bias)	Unclear risk	
Other bias	Unclear risk	

Milby et al. (2005)

**Table:** 

**Methods**
**Participants**
**Interventions**
**Outcomes**
**Notes**

Risk of bias table

**Table:** 

Bias	Authors' judgement	Support for judgement
Random sequence generation (selection bias)	Unclear risk	
Allocation concealment (selection bias)	Unclear risk	
Blinding of participants and personnel (performance bias)	Unclear risk	
Blinding of outcome assessment (detection bias)	Unclear risk	
Incomplete outcome data (attrition bias)	Unclear risk	
Selective reporting (reporting bias)	Unclear risk	
Other bias	Unclear risk	

Milby et al. (2008)

**Table:** 

**Methods**
**Participants**
**Interventions**
**Outcomes**
**Notes**

Risk of bias table

**Table:** 

Bias	Authors' judgement	Support for judgement
Random sequence generation (selection bias)	Unclear risk	
Allocation concealment (selection bias)	Unclear risk	
Blinding of participants and personnel (performance bias)	Unclear risk	
Blinding of outcome assessment (detection bias)	Unclear risk	
Incomplete outcome data (attrition bias)	Unclear risk	
Selective reporting (reporting bias)	Unclear risk	
Other bias	Unclear risk	

Milby et al. (2010)

**Table:** 

**Methods**
**Participants**
**Interventions**
**Outcomes**
**Notes**

Risk of bias table

**Table:** 

Bias	Authors' judgement	Support for judgement
Random sequence generation (selection bias)	Unclear risk	
Allocation concealment (selection bias)	Unclear risk	
Blinding of participants and personnel (performance bias)	Unclear risk	
Blinding of outcome assessment (detection bias)	Unclear risk	
Incomplete outcome data (attrition bias)	Unclear risk	
Selective reporting (reporting bias)	Unclear risk	
Other bias	Unclear risk	

O'Campo et al. (2016)

**Table:** 

**Methods**
**Participants**
**Interventions**
**Outcomes**
**Notes**

Risk of bias table

**Table:** 

Bias	Authors' judgement	Support for judgement
Random sequence generation (selection bias)	Unclear risk	
Allocation concealment (selection bias)	Unclear risk	
Blinding of participants and personnel (performance bias)	Unclear risk	
Blinding of outcome assessment (detection bias)	Unclear risk	
Incomplete outcome data (attrition bias)	Unclear risk	
Selective reporting (reporting bias)	Unclear risk	
Other bias	Unclear risk	

O'Connell et al. (2012)

**Table:** 

**Methods**
**Participants**
**Interventions**
**Outcomes**
**Notes**

Risk of bias table

**Table:** 

Bias	Authors' judgement	Support for judgement
Random sequence generation (selection bias)	Unclear risk	
Allocation concealment (selection bias)	Unclear risk	
Blinding of participants and personnel (performance bias)	Unclear risk	
Blinding of outcome assessment (detection bias)	Unclear risk	
Incomplete outcome data (attrition bias)	Unclear risk	
Selective reporting (reporting bias)	Unclear risk	
Other bias	Unclear risk	

Patterson et al. (2013)

**Table:** 

**Methods**
**Participants**
**Interventions**
**Outcomes**
**Notes**

Risk of bias table

**Table:** 

Bias	Authors' judgement	Support for judgement
Random sequence generation (selection bias)	Unclear risk	
Allocation concealment (selection bias)	Unclear risk	
Blinding of participants and personnel (performance bias)	Unclear risk	
Blinding of outcome assessment (detection bias)	Unclear risk	
Incomplete outcome data (attrition bias)	Unclear risk	
Selective reporting (reporting bias)	Unclear risk	
Other bias	Unclear risk	

Pleace and Bretherton (2013)

**Table:** 

**Methods**
**Participants**
**Interventions**
**Outcomes**
**Notes**

Risk of bias table

**Table:** 

Bias	Authors' judgement	Support for judgement
Random sequence generation (selection bias)	Unclear risk	
Allocation concealment (selection bias)	Unclear risk	
Blinding of participants and personnel (performance bias)	Unclear risk	
Blinding of outcome assessment (detection bias)	Unclear risk	
Incomplete outcome data (attrition bias)	Unclear risk	
Selective reporting (reporting bias)	Unclear risk	
Other bias	Unclear risk	

Poremski et al. (2016)

**Table:** 

**Methods**
**Participants**
**Interventions**
**Outcomes**
**Notes**

Risk of bias table

**Table:** 

Bias	Authors' judgement	Support for judgement
Random sequence generation (selection bias)	Unclear risk	
Allocation concealment (selection bias)	Unclear risk	
Blinding of participants and personnel (performance bias)	Unclear risk	
Blinding of outcome assessment (detection bias)	Unclear risk	
Incomplete outcome data (attrition bias)	Unclear risk	
Selective reporting (reporting bias)	Unclear risk	
Other bias	Unclear risk	

Rezansoff et al. (2017)

**Table:** 

**Methods**
**Participants**
**Interventions**
**Outcomes**
**Notes**

Risk of bias table

**Table:** 

Bias	Authors' judgement	Support for judgement
Random sequence generation (selection bias)	Unclear risk	
Allocation concealment (selection bias)	Unclear risk	
Blinding of participants and personnel (performance bias)	Unclear risk	
Blinding of outcome assessment (detection bias)	Unclear risk	
Incomplete outcome data (attrition bias)	Unclear risk	
Selective reporting (reporting bias)	Unclear risk	
Other bias	Unclear risk	

Russolillo et al. (2014)

**Table:** 

**Methods**
**Participants**
**Interventions**
**Outcomes**
**Notes**

Risk of bias table

**Table:** 

Bias	Authors' judgement	Support for judgement
Random sequence generation (selection bias)	Unclear risk	
Allocation concealment (selection bias)	Unclear risk	
Blinding of participants and personnel (performance bias)	Unclear risk	
Blinding of outcome assessment (detection bias)	Unclear risk	
Incomplete outcome data (attrition bias)	Unclear risk	
Selective reporting (reporting bias)	Unclear risk	
Other bias	Unclear risk	

Sadowski et al. (2009)

**Table:** 

**Methods**
**Participants**
**Interventions**
**Outcomes**
**Notes**

Risk of bias table

**Table:** 

Bias	Authors' judgement	Support for judgement
Random sequence generation (selection bias)	Unclear risk	
Allocation concealment (selection bias)	Unclear risk	
Blinding of participants and personnel (performance bias)	Unclear risk	
Blinding of outcome assessment (detection bias)	Unclear risk	
Incomplete outcome data (attrition bias)	Unclear risk	
Selective reporting (reporting bias)	Unclear risk	
Other bias	Unclear risk	

Sewel (2016)

**Table:** 

**Methods**
**Participants**
**Interventions**
**Outcomes**
**Notes**

Risk of bias table

**Table:** 

Bias	Authors' judgement	Support for judgement
Random sequence generation (selection bias)	Unclear risk	
Allocation concealment (selection bias)	Unclear risk	
Blinding of participants and personnel (performance bias)	Unclear risk	
Blinding of outcome assessment (detection bias)	Unclear risk	
Incomplete outcome data (attrition bias)	Unclear risk	
Selective reporting (reporting bias)	Unclear risk	
Other bias	Unclear risk	

Shern et al. (1997)

**Table:** 

**Methods**
**Participants**
**Interventions**
**Outcomes**
**Notes**

Risk of bias table

**Table:** 

Bias	Authors' judgement	Support for judgement
Random sequence generation (selection bias)	Unclear risk	
Allocation concealment (selection bias)	Unclear risk	
Blinding of participants and personnel (performance bias)	Unclear risk	
Blinding of outcome assessment (detection bias)	Unclear risk	
Incomplete outcome data (attrition bias)	Unclear risk	
Selective reporting (reporting bias)	Unclear risk	
Other bias	Unclear risk	

Shern (2000)

**Table:** 

**Methods**
**Participants**
**Interventions**
**Outcomes**
**Notes**

Risk of bias table

**Table:** 

Bias	Authors' judgement	Support for judgement
Random sequence generation (selection bias)	Unclear risk	
Allocation concealment (selection bias)	Unclear risk	
Blinding of participants and personnel (performance bias)	Unclear risk	
Blinding of outcome assessment (detection bias)	Unclear risk	
Incomplete outcome data (attrition bias)	Unclear risk	
Selective reporting (reporting bias)	Unclear risk	
Other bias	Unclear risk	

Siegel et al. (2006)

**Table:** 

**Methods**
**Participants**
**Interventions**
**Outcomes**
**Notes**

Risk of bias table

**Table:** 

Bias	Authors' judgement	Support for judgement
Random sequence generation (selection bias)	Unclear risk	
Allocation concealment (selection bias)	Unclear risk	
Blinding of participants and personnel (performance bias)	Unclear risk	
Blinding of outcome assessment (detection bias)	Unclear risk	
Incomplete outcome data (attrition bias)	Unclear risk	
Selective reporting (reporting bias)	Unclear risk	
Other bias	Unclear risk	

Sosin et al. (1996)

**Table:** 

**Methods**
**Participants**
**Interventions**
**Outcomes**
**Notes**

Risk of bias table

**Table:** 

Bias	Authors' judgement	Support for judgement
Random sequence generation (selection bias)	Unclear risk	
Allocation concealment (selection bias)	Unclear risk	
Blinding of participants and personnel (performance bias)	Unclear risk	
Blinding of outcome assessment (detection bias)	Unclear risk	
Incomplete outcome data (attrition bias)	Unclear risk	
Selective reporting (reporting bias)	Unclear risk	
Other bias	Unclear risk	

Srebnik et al. (2013)

**Table:** 

**Methods**
**Participants**
**Interventions**
**Outcomes**
**Notes**

Risk of bias table

**Table:** 

Bias	Authors' judgement	Support for judgement
Random sequence generation (selection bias)	Unclear risk	
Allocation concealment (selection bias)	Unclear risk	
Blinding of participants and personnel (performance bias)	Unclear risk	
Blinding of outcome assessment (detection bias)	Unclear risk	
Incomplete outcome data (attrition bias)	Unclear risk	
Selective reporting (reporting bias)	Unclear risk	
Other bias	Unclear risk	

Stefancic and Tsemberis (2007)

**Table:** 

**Methods**
**Participants**
**Interventions**
**Outcomes**
**Notes**

Risk of bias table

**Table:** 

Bias	Authors' judgement	Support for judgement
Random sequence generation (selection bias)	Unclear risk	
Allocation concealment (selection bias)	Unclear risk	
Blinding of participants and personnel (performance bias)	Unclear risk	
Blinding of outcome assessment (detection bias)	Unclear risk	
Incomplete outcome data (attrition bias)	Unclear risk	
Selective reporting (reporting bias)	Unclear risk	
Other bias	Unclear risk	

Stergiopoulos et al. (2015)

**Table:** 

**Methods**
**Participants**
**Interventions**
**Outcomes**
**Notes**

Risk of bias table

**Table:** 

Bias	Authors' judgement	Support for judgement
Random sequence generation (selection bias)	Unclear risk	
Allocation concealment (selection bias)	Unclear risk	
Blinding of participants and personnel (performance bias)	Unclear risk	
Blinding of outcome assessment (detection bias)	Unclear risk	
Incomplete outcome data (attrition bias)	Unclear risk	
Selective reporting (reporting bias)	Unclear risk	
Other bias	Unclear risk	

Tsemberis et al. (2003)

**Table:** 

**Methods**
**Participants**
**Interventions**
**Outcomes**
**Notes**

Risk of bias table

**Table:** 

Bias	Authors' judgement	Support for judgement
Random sequence generation (selection bias)	Unclear risk	
Allocation concealment (selection bias)	Unclear risk	
Blinding of participants and personnel (performance bias)	Unclear risk	
Blinding of outcome assessment (detection bias)	Unclear risk	
Incomplete outcome data (attrition bias)	Unclear risk	
Selective reporting (reporting bias)	Unclear risk	
Other bias	Unclear risk	

Tsemberis et al. (2004)

**Table:** 

**Methods**
**Participants**
**Interventions**
**Outcomes**
**Notes**

Risk of bias table

**Table:** 

Bias	Authors' judgement	Support for judgement
Random sequence generation (selection bias)	Unclear risk	
Allocation concealment (selection bias)	Unclear risk	
Blinding of participants and personnel (performance bias)	Unclear risk	
Blinding of outcome assessment (detection bias)	Unclear risk	
Incomplete outcome data (attrition bias)	Unclear risk	
Selective reporting (reporting bias)	Unclear risk	
Other bias	Unclear risk	

Turner Research & Strategy (2015)

**Table:** 

**Methods**
**Participants**
**Interventions**
**Outcomes**
**Notes**

Risk of bias table

**Table:** 

Bias	Authors' judgement	Support for judgement
Random sequence generation (selection bias)	Unclear risk	
Allocation concealment (selection bias)	Unclear risk	
Blinding of participants and personnel (performance bias)	Unclear risk	
Blinding of outcome assessment (detection bias)	Unclear risk	
Incomplete outcome data (attrition bias)	Unclear risk	
Selective reporting (reporting bias)	Unclear risk	
Other bias	Unclear risk	

## SOURCES OF SUPPORT

### INTERNAL SOURCES

- No sources of support provided

### EXTERNAL SOURCES

- This review is funded by the Centre for Homelessness Impact., UK

This review is funded by the Centre for Homelessness Impact.

## Footnotes

1 In Table 5 this effect size is negative because it describes the difference as the mean housing stability score for *basic/unconditional* interventions minus the mean housing stability score for *high/unconditional* interventions.

## APPENDIX A Dyo

**Table:**

**1. Bibliographic information**	
Article ID	FREETEXT
Linked articles	FREETEXT
Extracted by	FREETEXT
Checked by	FREETEXT
Year of publication	FREETEXT
Type of publication	1. Journal Article 2. Book/book chapter 3. Government report 4. Conference proceedings 5. Presentation 6. Thesis or Dissertation 7. Unpublished report 8. Other (please specify)
Location of study *The location in which the study is set **not** where the study authors are based*.	1. UK 2. ROI 3. Rest of Europe 4. USA 5. Canada 6. South America 7. Central America 8. Oceania 9. Middle‐East 10. Asia 11. Africa 12. Other (Please Specify)
	Not Specified
Study funding sources	1. Research council funding 2. University scholarships and bursaries 3. Salaried research assistantships from university departments 4. Grants or loans from trusts and charities 5. Local enterprise initiatives 6. Company sponsorship 7. Government loans 8. EU Scholarships 9. Industry sponsorship 10. Other (please specify)
Possible conflicts of interest	1. Yes, possible/definite conflict of interest 2. No, study appears to be free of CoI 3. Can't tell

**2. Participant information**	
Recruitment setting *Where were participants recruited from?*	1. Clinical setting 2. Accommodation for individuals experiencing homelessness 3. Family home 4. The street 5. Community setting 6. Referred by friends or family 7. Referred by medical health professional 8. Housing Agency 9. Other (Please specify)
Homelessness Status at intake *Describe the housing status of the sample at intake and/or any information given about housing status prior to intake. Tick all that apply and try to extract numbers were available*. *Homelessness is defined as those individuals who are sleeping 'rough' (sometimes defined as street homeless), those in temporary accommodation (such as shelters and hostels), those in insecure accommodation (such as those facing eviction or in abusive or unsafe environments), and those in inadequate accommodation (environments which are unhygienic and/or overcrowded)*.	1. Sleeping 'Rough' (or rooflessness) 2. Temporary Accommodation 3. Insecure Accommodation 4. Inadequate Accommodation 5. Involuntary sharing, for example, domestic violence 6. Hidden/concealed homelessness 7. Other (please specify)
	Not Specified
Geographical context *Where participants receive treatment?*	1. Urban 2. Rural 3. Suburban 4. Mixed 5. Other (please specify)
	Not Specified
Gender *% (actual number)*	FREETEXT
Age *Extract mean age, SD and range*. *Choose multiple options if the analysis is reported separately for different age groups*.	1. Under 25 2. 25 and Over
Complexity of needs *What other challenges does the individual face, if any, aside from the risk or experience of homelessness?* *High Risk of Harm and/or Exploitation ‐ For example, women in shelters, newcomer families, refugee/asylum seeker, care leavers*	1. Poor Physical Health 2. Poor Mental Health 3. Incarceration 4. Substance Abuse Issues 5. Care leaver 6. Limited access to integrated support services 7. High Risk of Harm and/or Exploitation 8. Other (please specify) Not RelevantNot Specified
Mental health status	1. Receiving treatment 2. Not receiving treatment 3. Other (please specify)
	Not relevant
	Not Specified
Substance use status	1. Receiving treatment 2. Not receiving treatment 3. Other (please specify)
	Not relevant
	Not Specified
Homelessness status *Homelessness is defined as those individuals who are sleeping “rough” (sometimes defined as street homeless), those in temporary accommodation (such as shelters and hostels), those in insecure accommodation (such as those facing eviction or in abusive or unsafe environments), and those in inadequate accommodation (environments which are unhygienic and/or overcrowded)*.	1. Sleeping “rough” 2. Temporary accommodation 3. Insecure accommodation 4. Inadequate accommodation 5. Other (please specify)
	Not Specified
Family vs. No Family *Family* = *any child involved* *Non‐family* = *single person or couple without children* *If mixed sample select both and describe*	1. Family 2. Non‐Family
	Not Specified
Sample size of treatment group*Number of people assigned to treatment. If more than one treatment group extract all and be clear which group is which*.	FREETEXT
Sample size of control group *Number of people assigned to control. If more than one control group extract all and be clear which group is which*.	FREETEXT

**3. Intervention information**	
How many intervention arms in this trial? *List how many study arms there are and given each a name. e.g. Intervention* = *Critical Time Intervention; Control* = *Treatment as usual* *If there is more than one intervention arm go to the "Study Arm" tab and add the RELEVANT study arms. You must then extract data for each relevant study arm*.	FREETEXT
Name of intervention *Write in the name of the program, intervention, or treatment under study. This may be specific like “critical time intervention” or it may be something more generic like “supported housing”*	FREETEXT
Briefly Describe the intervention *Briefly describe the intervention, what participants are offered and any important factors such as conditionality, nature of housing, case management, substance abuse treatment included etc*.	FREETEXT
Theory of change *How does the intervention aim to bring about change? What is the underlying theoretical rationale for why the intervention might work to improve outcomes?* *If not specified write "not specified"*	FREETEXT
What is the size of accommodation/How many beds?	FREETEXT
Duration of treatment period from start to finish *In the dosage items, we are interested in the amount of treatment received by the participants. If the treatment was delivered directly to participants, the authors will probably provide at least some information about dosage and you can code these items accordingly. If minimal information is provided, you should try to give estimates for these items if you can come up with a reasonable estimate*.	FREETEXT
Timing *Frequency of contact between participants and provider/program activity*	1. Once a month2. Less than weekly3. Once a week4. 1‐2 times a week5. 2 times a week6. 2‐3 times a week7. 3 times a week8. 3‐4 times a week9. 4 times a week10. Daily contactCan't Estimate
Length of each individual session *How long does each contact/session last?*	FREETEXT
Study Personnel *The primary individual/s who have direct contact with the participants served by the program*. *If the report is the author's dissertation (or based on the author's dissertation), then code as "Graduate Researcher"*. *If the delivery is performed by graduate or undergraduate students assisting the author then select "Grad/Undergrad Students"*. *Code “Self‐directed” for studies where electronic/computer programs are used*. *If the intervention is solely environmental i.e. community housing, then code “environmental change”*	1. Graduate Researcher 2. Grad/Undergrad Students 3. Author 4. Homelessness professional
	*Includes case manager, social worker, outreach worker*
	5. Peers 6. Interventionist (Not Hired by Researcher) 7. Interventionist (Hired by Researcher) 8. Self‐Directed 9. Medical Professionals 10. Other (please specify)
	Not Specified
Did provider receive specialised training? *This refers to whether or not the “interventionist” received specialised training to equip them to deliver the intervention proficiently*.	1. Yes 2. The interventionist IS program developer 3. No
	Not specified
Resource requirements *Time, staff, housing provision etc*	FREETEXT
Cost	FREETEXT

**4a. Study Design**	
Design *The studies included in all reviews must include an intervention group and at least one untrained control group. Control groups can include placebo, no treatment, waitlist, or treatments vs “treatment as usual.” Any study which includes one group pre‐test/post‐test or in which a treatment group is only compared to another treatment group will not be eligible for inclusion*.	1. Randomised control trial
	*Individual or cluster randomised*
	2. Non‐randomised control trial
What do control subjects receive?	1. Placebo 2. Treatment as usual 3. No treatment 4. Not specified
1. *Placebo (or attention) treatment. Group gets some attention or a sham treatment* 2. *Treatment as usual. Group gets “usual” handling instead of some special treatment*. 3. *No treatment. Group gets no treatment at all*.	
Unit of allocation *Individual (i.e., some were assigned to treatment group, some to comparison group)* *Group (i.e., whole subsets assigned to treatment and comparison groups)* *Regions (i.e., region assigned as an intact unit)*	1. Individual 2. Group 3. Regions 4. Other (Please Specify)
Not Specified
Method of assignment	1. Randomly after matching 2. Randomly without matching 3. Regression discontinuity design 4. Cluster assigned 5. Wait list control 6. Non‐random, but matched 7. Other (Please Specify)
*Method of group assignment. How participants/units were assigned to groups. This item focuses on the initial method of assignment to groups, regardless of subsequent degradations due to attrition, refusal, etc. prior to treatment onset*.	
1. *Randomly after matching, yoking, stratification, blocking, etc. The entire sample is matched or blocked first, then assigned to treatment and comparison groups within pairs or blocks. This does not refer to blocking after treatment for the data analysis*. 2. *Randomly without matching, etc. This also includes cases when every other person goes to the control group*. 3. *Regression discontinuity design: quantitative cutting point defines groups on some continuum (this is rare)*. 4. *Cluster assigned, this is to be used in cluster assignment studies only, specify the number of clusters in the treatment group and the number of clusters in control*. 5. *Wait list control or other quasi‐random procedure presumed to produce comparable groups (no obvious differences). This applies to groups which have individuals apparently randomly assigned by some naturally occurring process, e.g. first person to walk in the door. The key here is that the procedure used to select groups doesn't involve individual characteristics of persons so that the groups generated should be essentially equivalent*. 6. *Non‐random, but matched: Matching refers to the process by which comparison groups are generated by identifying individuals or groups that are comparable to the treatment group using various characteristics of the treatment group. Matching can be done individually, e.g., by selecting a control subject for each intervention subject who is the same age, gender, and so forth, or on a group basis*.	
	Not Specified
Was there >20% attrition in either/both groups? *Attrition occurs when participants are lost from an intervention over time or over a series of sequential processes. Studies may describe this as “lost to follow‐up,” or “drop outs.”*	FREETEXT

**4b. Non‐random studies**	
How were groups matched? *If matching was used prior to assignment of condition, how were groups matched?*	1. Matched on Pre‐test measure 2. Matched on personal characteristics 3. Matched on demographics 4. Groups weren't matched 5. Other (please specify)
	Not specified
Was the equivalence of groups tested at pre‐test?	FREETEXT
Results of statistical comparisons of pre‐test differences	1. No statistically significant differences 2. Significant differences judged unimportant by coder 3. Significant differences judged of uncertain importance by coder 4. Significant differences judged important by coder 5. Other (please specify)
Were there pre‐test adjustments?	FREETEXT

**5. Qualitative information**	
Qualitative methods used	FREETEXT
Data analysis technique and procedure	FREETEXT
Was the intervention implemented as intended?	1. Yes 2. No
	Not specified
How was this measured?	FREETEXT
What implementation and process factors impact intervention delivery?	1. Contextual factors 2. Policy makers/funders 3. Programme managers/Implementing agency, 4. Staff/case workers 5. Recipients

**6. Assessing quality in RCTs (Cochranes ROB2 tool)**	
**Domain 1:** Risk of bias arising from the randomization process	
1.1 Was the allocation sequence random?	1. Yes 2. Probably yes 3. Probably No 4. No
1.2 Was the allocation sequence concealed until participants were enrolled and assigned to interventions?	1. Yes 2. Probably yes 3. Probably No 4. No
1.3 Did baseline differences between intervention groups suggest a problem with the randomization process?	1. Yes 2. Probably yes 3. Probably No 4. No
Risk‐of‐bias judgement	1. Low 2. High 3. Some concerns
Optional: What is the predicted direction of bias arising from the randomization process?	1. Favours experimental 2. Favours comparator 3. Towards null 4. Away from null 5. Unpredictable
**Domain 2:** Risk of bias due to deviations from the intended interventions (effect of assignment to intervention)	
2.1. Were participants aware of their assigned intervention during the trial?	1. Yes 2. Probably yes 3. Probably No 4. No
2.2. Were carers and people delivering the interventions aware of participants' assigned intervention during the trial?	1. Yes 2. Probably yes 3. Probably No 4. No
2.3. If Y/PY/NI to 2.1 or 2.2: Were there deviations from the intended intervention that arose because of the experimental context?	1. Yes 2. Probably yes 3. Probably No 4. No
2.4. If Y/PY to 2.3: Were these deviations from intended intervention balanced between groups?	1. Yes 2. Probably yes 3. Probably No 4. No
2.5 If N/PN/NI to 2.4: Were these deviations likely to have affected the outcome?	1. Yes 2. Probably yes 3. Probably No 4. No
2.6 Was an appropriate analysis used to estimate the effect of assignment to intervention?	1. Yes 2. Probably yes 3. Probably No 4. No
2.7 If N/PN/NI to 2.6: Was there potential for a substantial impact (on the result) of the failure to analyse participants in the group to which they were randomized?	1. Yes 2. Probably yes 3. Probably No 4. No
Risk‐of‐bias judgement	1. Low 2. High 3. Some concerns
Optional: What is the predicted direction of bias due to deviations from intended interventions?	1. Favours experimental 2. Favours comparator 3. Towards null 4. Away from null 5. Unpredictable
**Domain 3:** Missing outcome data	
3.1 Were data for this outcome available for all, or nearly all, participants randomized?	1. Yes 2. Probably yes 3. Probably No 4. No
3.2 If N/PN/NI to 3.1: Is there evidence that result was not biased by missing outcome data?	1. Yes 2. Probably yes 3. Probably No 4. No
3.3 If N/PN to 3.2: Could missingness in the outcome depend on its true value?	1. Yes 2. Probably yes 3. Probably No 4. No
3.4 If Y/PY/NI to 3.3: Do the proportions of missing outcome data differ between intervention groups?	1. Yes 2. Probably yes 3. Probably No 4. No
3.5 If Y/PY/NI to 3.3: Is it likely that missingness in the outcome depended on its true value?	1. Yes 2. Probably yes 3. Probably No 4. No
Risk‐of‐bias judgement	1. Low 2. High 3. Some concerns
Optional: What is the predicted direction of bias due to missing outcome data?	1. Favours experimental 2. Favours comparator 3. Towards null 4. Away from null 5. Unpredictable
**Domain 4:** Risk of bias in measurement of the outcome	
4.1 Was the method of measuring the outcome inappropriate?	1. Yes 2. Probably yes 3. Probably No 4. No
4.2 Could measurement or ascertainment of the outcome have differed between intervention groups?	1. Yes 2. Probably yes 3. Probably No 4. No
4.3 If N/PN/NI to 4.1 and 4.2: Were outcome assessors aware of the intervention received by study participants?	1. Yes 2. Probably yes 3. Probably No 4. No
4.4 If Y/PY/NI to 4.3: Could assessment of the outcome have been influenced by knowledge of intervention received?	1. Yes 2. Probably yes 3. Probably No 4. No
4.5 If Y/PY/NI to 4.4: Is it likely that assessment of the outcome was influenced by knowledge of intervention received?	1. Yes 2. Probably yes 3. Probably No 4. No
Risk‐of‐bias judgement	1. Low 2. High 3. Some concerns
Optional: What is the predicted direction of bias in measurement of the outcome?	1. Favours experimental 2. Favours comparator 3. Towards null 4. Away from null 5. Unpredictable
**Domain 5:** Risk of bias in selection of the reported result	
5.1 Was the trial analysed in accordance with a pre‐specified plan that was finalized before unblinded outcome data were available for analysis?	1. Yes 2. Probably yes 3. Probably No 4. No
Is the numerical result being assessed likely to have been selected, on the basis of the results, from…	
5.2. … multiple outcome measurements (e.g. scales, definitions, time points) within the outcome domain?	1. Yes 2. Probably yes 3. Probably No 4. 4. No
5.3 … multiple analyses of the data?	1. Yes 2. Probably yes 3. Probably No 4. No
Risk‐of‐bias judgement	1. Low 2. High 3. Some concerns
Optional: What is the predicted direction of bias due to selection of the reported result?	1. Favours experimental 2. Favours comparator 3. Towards null 4. Away from null 5. Unpredictable
**Overall risk of bias**	
Risk‐of‐bias judgement	1. Low 2. High 3. Some concerns

**7. Assessing quality in Non‐random control trials (ROBINS‐I tool)**	
**Bias due to confounding**	
1.1 Is there potential for confounding of the effect of intervention in this study? **If N/PN to 1.1:** the study can be considered to be at low risk of bias due to confounding and no further signalling questions need be considered	1. Yes 2. Probably yes 3. Probably No 4. No
**If Y/PY to 1.1**: determine whether there is a need to assess time‐varying confounding:	1. Yes 2. Probably yes 3. Probably No 4. No
1.2. Was the analysis based on splitting participants' follow up time according to intervention received?**If N/PN**, answer questions relating to baseline confounding (1.4 to 1.6)**If Y/PY**, go to question 1.3.	1. Yes 2. Probably yes 3. Probably No 4. No
1.3. Were intervention discontinuations or switches likely to be related to factors that are prognostic for the outcome? **If N/PN**, answer questions relating to baseline confounding (1.4 to 1.6) **If Y/PY**, answer questions relating to both baseline and time‐varying confounding (1.7 and 1.8)	1. Yes 2. Probably yes 3. Probably No 4. No
**Questions relating to baseline confounding only**	
1.4. Did the authors use an appropriate analysis method that controlled for all the important confounding domains?	1. Yes 2. Probably yes 3. Probably No 4. No
1.5. **If Y/PY to 1.4**: Were confounding domains that were controlled for measured validly and reliably by the variables available in this study?	1. Yes 2. Probably yes 3. Probably No 4. No
1.6. Did the authors control for any post‐intervention variables that could have been affected by the intervention?	1. Yes 2. Probably yes 3. Probably No 4. No
**Questions relating to baseline and time‐varying confounding**	
1.7. Did the authors use an appropriate analysis method that controlled for all the important confounding domains and for time‐varying confounding?	1. Yes 2. Probably yes 3. Probably No 4. No
1.8. If **Y/PY** to 1.7: Were confounding domains that were controlled for measured validly and reliably by the variables available in this study?	1. Yes 2. Probably yes 3. Probably No 4. No
Risk‐of‐bias judgement	1. Low 2. Moderate 3. Serious 4. Critical
Optional: What is the predicted direction of bias due to confounding?	1. Favours experimental 2. Favours comparator 3. Unpredictable
**Bias in selection of participants into the study**	
2.1. Was selection of participants into the study (or into the analysis) based on participant characteristics observed after the start of intervention? **If N/PN to 2.1:** go to 2.4	1. Yes 2. Probably yes 3. Probably No 4. No
2.2. **If Y/PY to 2.1**: Were the post‐intervention variables that influenced selection likely to be associated with intervention?	1. Yes 2. Probably yes 3. Probably No 4. No
2.3 **If Y/PY to 2.2**: Were the post‐intervention variables that influenced selection likely to be influenced by the outcome or a cause of the outcome?	1. Yes 2. Probably yes 3. Probably No 4. No
2.4. Do start of follow‐up and start of intervention coincide for most participants?	1. Yes 2. Probably yes 3. Probably No 4. No
2.5. **If Y/PY to 2.2 and 2.3, or N/PN to 2.4**: Were adjustment techniques used that are likely to correct for the presence of selection biases?	1. Yes 2. Probably yes 3. Probably No 4. No
Risk‐of‐bias judgement	1. Low 2. Moderate 3. Serious 4. Critical
Optional: What is the predicted direction of bias due to selection of participants into the study?	1. Favours experimental 2. Favours comparator 3. Towards null 4. Away from null 5. Unpredictable
**Bias in classification of interventions**	
3.1 Were intervention groups clearly defined?	1. Yes 2. Probably yes 3. Probably No 4. No
3.2 Was the information used to define intervention groups recorded at the start of the intervention?	1. Yes 2. Probably yes 3. Probably No 4. No
3.3 Could classification of intervention status have been affected by knowledge of the outcome or risk of the outcome?	1. Yes 2. Probably yes 3. Probably No 4. No
Risk‐of‐bias judgement	1. Low 2. Moderate 3. Serious 4. Critical
Optional: What is the predicted direction of bias due to classification of interventions?	1. Favours experimental 2. Favours comparator 3. Towards null 4. Away from null 5. Unpredictable
**Bias due to deviations from intended interventions**	
If your aim for this study is to assess the effect of assignment to intervention, answer questions 4.1 and 4.2	
4.1. Were there deviations from the intended intervention beyond what would be expected in usual practice?	1. Yes 2. Probably yes 3. Probably No 4. No
4.2. **If Y/PY to 4.1**: Were these deviations from intended intervention unbalanced between groups *and* likely to have affected the outcome?	1. Yes 2. Probably yes 3. Probably No 4. No
If your aim for this study is to assess the effect of starting and adhering to intervention, answer questions 4.3 to 4.6	
4.3. Were important co‐interventions balanced across intervention groups?	1. Yes 2. Probably yes 3. Probably No 4. No
4.4. Was the intervention implemented successfully for most participants?	1. Yes 2. Probably yes 3. Probably No 4. No
4.5. Did study participants adhere to the assigned intervention regimen?	1. Yes 2. Probably yes 3. Probably No 4. No
4.6. **If N/PN to 4.3, 4.4 or 4.5**: Was an appropriate analysis used to estimate the effect of starting and adhering to the intervention?	1. Yes 2. Probably yes 3. Probably No 4. No
Risk‐of‐bias judgement	1. Low 2. Moderate 3. Serious 4. Critical
Optional: What is the predicted direction of bias due to deviations from the intended interventions?	1. Favours experimental 2. Favours comparator 3. Towards null 4. Away from null 5. Unpredictable
**Bias due to missing data**	
5.1 Were outcome data available for all, or nearly all, participants?	1. Yes 2. Probably yes 3. Probably No 4. No
5.2 Were participants excluded due to missing data on intervention status?	1. Yes 2. Probably yes 3. Probably No 4. No
5.3 Were participants excluded due to missing data on other variables needed for the analysis?	1. Yes 2. Probably yes 3. Probably No 4. No
5.4 **If PN/N to 5.1, or Y/PY to 5.2 or 5.3**: Are the proportion of participants and reasons for missing data similar across interventions?	1. Yes 2. Probably yes 3. Probably No 4. No
5.5 **If PN/N to 5.1, or Y/PY to 5.2 or 5.3**: Is there evidence that results were robust to the presence of missing data?	1. Yes 2. Probably yes 3. Probably No 4. No
Risk of bias judgement	1. Low 2. Moderate 3. Serious 4. Critical
Optional: What is the predicted direction of bias due to missing data?	1. Favours experimental 2. Favours comparator 3. Towards null 4. Away from null 5. Unpredictable
**Bias in measurement of outcomes**	
6.1 Could the outcome measure have been influenced by knowledge of the intervention received?	1. Yes 2. Probably yes 3. Probably No 4. No
6.2 Were outcome assessors aware of the intervention received by study participants?	1. Yes 2. Probably yes 3. Probably No 4. No
6.3 Were the methods of outcome assessment comparable across intervention groups?	1. Yes 2. Probably yes 3. Probably No 4. No
6.4 Were any systematic errors in measurement of the outcome related to intervention received?	1. Yes 2. Probably yes 3. Probably No 4. No
Risk of bias judgement	1. Low 2. Moderate 3. Serious 4. Critical
Optional: What is the predicted direction of bias due to measurement of outcomes?	1. Favours experimental 2. Favours comparator 3. Towards null 4. Away from null 5. Unpredictable
**Bias in selection of the reported result**	
Is the reported effect estimate likely to be selected, on the basis of the results, from…	
7.1. … multiple outcome measurements within the outcome domain?	1. Yes 2. Probably yes 3. Probably No 4. No
7.2 … multiple analyses of the intervention‐outcome relationship?	1. Yes 2. Probably yes 3. Probably No 4. No
7.3 … different subgroups?	1. Yes 2. Probably yes 3. Probably No 4. No
Risk of bias judgement	1. Low 2. Moderate 3. Serious 4. Critical
Optional: What is the predicted direction of bias due to selection of the reported result?	1. Favours experimental 2. Favours comparator 3. Towards null 4. Away from null 5. Unpredictable
**Overall risk of bias**	
Risk‐of‐bias judgement	1. Low 2. Moderate 3. Serious 4. Critical

**8. Assessing quality in Qualitative studies (White and Keenan tool)**	
Are the evaluation questions clearly stated?	1. Yes 2. No
Is the qualitative methodology described?	1. Yes 2. No
Is the qualitative methodology appropriate to address the evaluation questions?	1. Yes 2. No 3. Insufficient detail
Is the recruitment or sampling strategy described?	1. Yes 2. No
Is the recruitment or sampling strategy appropriate to address the evaluation questions?	1. Yes 2. No 3. Insufficient detail
Are the researcher's own position, assumptions and possible biases outlined?	1. Yes 2. No
Have ethical considerations been sufficiently considered?	1. Yes 2. No 3. Insufficient detail
Is the data analysis approach adequately described?	1. Yes 2. No
Is the data analysis sufficiently rigorous?	1. Yes 2. No
Is there a clear statement of findings?	1. Yes 2. No
Are the research findings useful?	1. Yes 2. No

## APPENDIX B ADDITIONAL INTERVENTION CHARACTERISTIC

**Table:**

Study title	Intervention name	Intervention description	Control	Scattered versus congregate	Intervention setting and time	Typology
Appel (2012)	Keeping Home project" "Housing First approach	Keeping Home uses the Housing First approach to address the needs of homeless, mentally ill substance abusers. Specifically, Keeping Home secures market‐rate, scattered site apartments for seriously mentally ill methadone maintenance treatment patients and then uses assertive community treatment to address patients service needs	Usual treatment: available housing and support services	Scattered site	New York City, USA. Between March 2005 and March 2007	High unconditional
Brown (2016)	Housing First	Held at a 75‐unit single‐site HOUSING FIRST program. Residents were not required to abstain from substance use, nor are they required to participate in mental health or substance abuse treatment. However, staff provides assertive engagement to encourage participation. Participants in the HOUSING FIRST program received a breadth of intensive, consumer‐driven support services based on personal need and interest along with housing	Usual treatment: comparison group participants received usual care, comprising access to a variety of supports, including outpatient mental health and/or substance abuse treatment, sobering services, shelter, and/or other supportive housing programs	Congregate (75‐unit single site)	Washington, USA	High unconditional
Buchanan (2006)	Respite care	Provides short‐term shelter along with access to additional services (food, case management and referrals to permanent housing is provided). Conditional upon being capable of living in a drug and alcohol free environment	No intervention	Congregate	Chicago, Illinois, USA. Between October 1, 1998, and December 31, 2000	Moderate conditional
Buchanan (2009)	The Chicago Housing for Health Partnership (CHHP)	Two strands within the intervention, one is respite care and case management the other, for those unable to abstain from alcohol or drugs, is referral to shelter with case management	Unclear	Usual treatment: Referrals to overnight shelters or to interim housing providers. All usual care participants were eligible to receive case management through an existing Ryan White program in the hospital‐affiliated HIV/AIDS clinic	Chicago, Illinois, USA. October 2003–May 2006	High, conditional and unconditional, depending on participant
Cheng (2007)	HUD‐VASH	HUD‐VASH: VA intensive case management were linked to Section 8 housing vouchers, which provide rent subsidies to low‐income individuals with disabilities	Usual treatment: standard VA homeless services, which consisted of short termbroker case management linking users to VA and community services	Not specified	San Francisco, California (N = 107); San Diego, California (N = 91); New Orleans, Louisiana (N = 165); and Cleveland, Ohio (N = 97).Around 1992	Moderate unconditional
Gilmer (2010)	Full‐Service Partnerships (FSP)	The FSP programs implemented in San Diego County provide a combination of subsidized permanent housing and team based services with a focus on rehabilitation and recovery	No treatment	Some scattered, some congregate	San Diego, Caliifornia USA. Between October 2005‐June 2008	High conditional
Goering 2011	Chez Soi—Housing First	The Housing First model used here involves the provision of permanent, private housing units to qualifying individuals, with consumer choice on services and housing location being fundamental	Usual treatment: treatment as usual in each of the current systems of care; does not mean “no care”	Mix of both depending on city and intervention given. Congregate specifically in vancouver	Five Canadian cities of Vancouver, Winnipeg, Toronto, Montreal and Moncton	High unconditional
Goldfinger (1999)	Assigned to group homes, or assigned to independent apartments.	The seven group homes each accommodated between 6 and 10 tenants with shared living, dining, recreational, and kitchen facilities, but separate bedrooms. They provided 24‐h daily coverage, with project staff constantly at hand. All study participants were provided with an intensive case manager. Each participant met with the case manager at least once a week for counselling, hands‐on help with daily activities, and help with access to needed services	High unconditional: participants were given an individual apartment and case manager, all were encouraged,but not required, to participatein community mental healthcentre programs55 scattered housing	63 to congregate housing	Boston, Massachusetts, USA. 18 Months (January 1991 and March 1992)	High unconditional
Gulcur (2003)	Housing First	The experimental Housing First programme offered immediate access to independent housing without requiring psychiatric treatment or sobriety	Continuum of Care: users begin with outreach programmes and drop‐in centres, and then progress through a series of congregate living arrangements with varying levels of on‐site support	Independent housing (scattered)	New York, New York, USA	High unconditional
Howard (2011)	Crisis House or Patient preference of Crisis House	Womens Crisis Housing was available at two London centres where there were also psychiatric wards. The women's crisis houses were located in ordinary residential streets, had a domestic atmosphere and were funded by the UK National Health Service (NHS). Staff were available 24 h per day and were either nurses or healthcare workers with a background in mental health.	Usual Treatment: Usual NHS psychiatric hospital care	Congregate – Crisis houses	London, UK, Around 2007	Moderate conditional
Hwang (2011)	Supportive housing	Tenants had access to a shared kitchen facility, a drop‐in center offering meals and outreach services, as well as a medical and dental clinic providing free services. Individuals accepted into the program received rental subsidies and paid rent‐geared‐to‐income (not exceeding 30% of income). Specifically, tenant support workers assisted residents with mental illness to transition into the housing program.	Usual Treatment, but waitlist (no housing)	Congregate, 84 units in one building	Toronto, Canada. Between 2005 and 2008	High conditional
Kertesz (2007)	ACH Vs NACH Vs Control	All participants were offered behavioral day treatment services and randomly assigned to receive either no program‐provided housing during treatment (no housing), program provided housing contingent upon abstinence (abstinence‐contingent housing), or program‐ provided housing not contingent upon abstinence (nonabstinence‐contingent housing).	Usual outpatient treatment program	Not specified	Birmingham, Alabama, United states	Intervention 1, high conditional; Intervention 2, high unconditional
Larimer (2009)	Housing First	Participants were given a room at an apartment building. No treatment requirements, but on‐site case managers work to engage residents about substance use and life goals. Meals and on‐site health care services are also offered. No behavioural conditions are placed on participants.	Waitlist	Not specified	King county, Washington, United states	High unconditional
Levitt (2013)	Home to stay	The Home to Stay pilot was focused on 3 strategies: moving families out of shelter rapidly using a locally funded, temporary housing subsidy; securing sufficient household income to enable families to pay market rent on expiration of the subsidy; and connecting families to community‐based services that would help them to maintain housing stability after the termination of Home to Stay services.	Usual Treatment, servicestaffing and delivery vary widely across the 150New York City DHS family shelters, but sheltercaseworkers are typically assigned mixed caseloadsof approximately 25 families.	Not specified	New York City, New York, USA. Around 2010–2012	Moderate conditional
Lim (2017)	NYNY IIII	The NYNY III program was for young adults aging out of foster care provides affordable housing and access to various supportive services to help achieve independent lives, including case management, job training, and education support, and provides connections to health services.	Usual Treatment: government‐subsidized housing programs	Not specified	New York City, New York, USA. 2007–2010	High unconditional
Lim (2018)	New York Supportive Housing Program	The program followed the Housing First model with housing placement not being contingent on adhering to treatment or services	No treatment	Not specified	New York City, New York, USA. 2007–2010	High unconditional
Lipton (2000)	Intervention 1: High Intensity Housing Intervention 2: Moderate Intensity Housing Intervention 3: Low Intensity Housing	High‐intensity settings are exclusively dedicated to persons with serious mental illness. It had the highest intensity of housing requirements and services. Residents in moderate intensity settings have their own rooms or studio apartments, and cooking facilities were shared. Tenants at low‐intensity sites hold legally binding leases for individual furnished private rooms or small studio type apartments, with access to either shared or private bathrooms and cooking facilities.	No Intervention	Intervention 1: Congregate Intervention 2: Congregate Intervention 3: Mix of congregate and Scattered	New York City, New York, USA. 1990–1995	Intervention 1, high conditional; Intervention 2, moderate conditional
McHugo (2004)	Integrated housing services program	The integrated housing services approach provided comprehensive mental health services through intensive case management and housing services through dedicated teams that controlled a variety of housing settings	The ACT teams assisted users in finding and affording housing	Mostly congregate housing	Washington, USA	High unconditional
Milby (1996)	Enhanced Care	Some received abstinent contingent work therapy (44.9% of EC subjects) and housing (37.7% of EC subjects). Work therapy, day treatment focused on relapse prevention, social skills training, goal review, weekend planning, community resources for homeless persons.	Usual Treatment: twice weekly group and individual counselling, referral for medical treatment, housing and other agencies. Less frequent aftercare visits.	Not specified	Birmingham, Alabama, United States. Recruited between April 1990‐August 1991, not done beyond this.	High conditional
Milby (2000)	Day treatment +	Day treatment met week days from 07:30‐14:00 h and included lunch and transportation to and from shelters. The following groups were conducted: participant governed morning meeting, process group, AIDS education, relapse prevention training, goal development, goal review, assertiveness training, and many more services.	Usual treatment: once weeklyindividual and group counselling to substance abuseday treatment which included unique interventions forhomelessness in addition to substance abuse.	Group housing: congregate	Birmingham, Alabama, United States	High conditional
Milby (2008)	CM and CM+ (Contingency Management plus behavioral day treatment)	The study compared the abbreviated treatment model (offering contingency‐managed housing, vocational training and work therapy, i.e. CM) to the full treatment model (CM+), which included the same interventions plus extensive behavioral day treatment	Offering contingency‐managed housing, vocational training and work therapy.	Not specified	Birmingham, Alabama, USA. Participants were recruited, randomized, and treated from 2001 to 2004	High conditional
O'Connell (2012)	Housing and Urban Development–Veterans Affairs Supported Housing (HUD‐VASH) Intensive Care Management (ICM)	HUD VASH: intensive case management [ICM] plus rent subsidy vouchers ICM: Intensive case management only	Usual Treatment	Not specified	New Haven, Connecticut, USA. Around 1992–1995	Moderate unconditional
Sadowski (2009)	Housing and Case management (HOUSING FIRST)	Subjects are offered interim housing upon discharge from enrolling hospitalizations, followed by stable housing within 90 days. They have a case manager at each stage (hospital, respite/interim housing, and stable housing)	Usual Treatment: these patients receive usual social services for hospital discharge planning	Mix of scattered and congregate	Chicago, Illinois, USA. Around 2008	High unconditional
Shern (1997)	New York Street Study—specialised housing	The Boston project compared congregate consumer‐run housing with independent living. In New York specialized housing for homeless persons with severe mental illness was the primary housing resource. The San Diego project tested the importance of Section 8 housing certificates in obtaining and maintaining housing.	Usual treatment, usually some sort of case management	Varied. Some congregate. Some scattered	Baltimore, Boston, San Diego, New York City, USA	Moderate conditional
Siegel (2006)	Supported Housing vs community residences	Two types of supported housing. (1) tenants, mostly living alone, reside in studio or one‐bedroom apartments. No preconditions (e.g. sobriety) for housing, with ACT also provided. (2) Sobriety a requirement. Could be asked to leave if they did not maintain good neighbour status—for example, without too many complaints from neighbors. On‐site crisis services are continuously available to tenants in coordination with a New York City psychiatric emergency service	Community Residences: The three agencies that operate thenine community residences provideintensive supportive services in theresidences, which are only for personswith mental illness.	Supported Housing (congregate) vs. community residences, control (scattered)	New York City, USA	Intervention 1, high unconditional; Intervention 2, high conditional
Sosin (1996)	Housing and case management Case management alone	Housing and case management: This is conditional on abstinence and complying with treatment plan. The housing intervention provided the case management model along with supported housing. Brokerage case management without direct provision of services	No Treatment—"The users placed in the control condition were referred by the relevant short‐term program staff to an outpatient or inpatient substance abuse agency, to welfare offices''	Not specified	Chicago, United States. Recruitment around 1991–1992, intervention took place around 1995	Moderate conditional
Srebnik (2013)	Housing First	Intervention is characterized by rapid placement from homelessness directly into permanent housing, supported by assertive on‐site engagement and services but no requirement to participate or to achieve or maintain sobriety	No treatment	Congregate: BAH set aside 20 units at a newly renovated downtown building	King County, the City of Seattle, Washington, USA	High unconditional
Stefancic and Tsemberis (2007)	Pathways (HOUSING FIRST) Consortium (HOUSING FIRST)	Pathways to Housing (HOUSING FIRST) and Consortium (HOUSING FIRST) both provide Housing First services, in the form of independent scatter‐site apartments and ACT. Pathways had a more straightforward HOUSING FIRST approach, whereas Consortium was a newly formed mix of treatment and housing agencies from within the county but with no prior experience operating Housing First	Usual Treatment: usual array of services that included shelter basedprograms and transitional housing	Scatter Site	New York City, USA. 2000–2002	High unconditional

## APPENDIX C COST OF INTERVENTIONS

It was not possible to meaningfully or quantitatively synthesise any cost data that were reported in the included studies, due to the various and diverse ways in which such data were collected and analysed. Thus, a narrative synthesis is presented instead.

Mennemeyer (2017) summarised the cost effectiveness, per participant, of various interventions at six months. In this group of studies, a range of accommodation interventions—along with work therapy, day treatment and counselling—were offered to the intervention group. The control group received similar therapies, however, usually without any housing intervention. Both intervention arms were conditional upon sobriety. Mennemeyer (2017) includes reported average costs such as direct programme treatment, estimated costs of time spent in jail and in hospital, and the estimated cost to society when an individual failed to maintain abstinence.

Throughout this study, the intervention arm was much more costly than the control arm, however, the research team claim that this cost is justified due to the improved outcomes of the participants involved. In the study the review team name “Homeless 3,” authors created a new intervention called NACH (nonabstinent contingent housing), which was more cost effective compared to other intervention and control arms.

Second, in a large study named “Chez Soi” (Goering, 2012), the control group received treatment as usual; they were directed to existing community resources, which included supports, such as homeless outreach, support centres and mental health resources. The authors discuss the economic implications of this brand of Housing First intervention while the savings from other housing and services are balanced against the investment in Chez Soi for the total group and for a group of the highest previous service users. The average shelter, health and justice costs for one year were $23,849 for the treatment group and $14,599 for the intervention group. The difference of $9250 partially offset the annual intervention cost of $17,160, resulting in an average net investment of $7910 per person per year to deliver the intervention. For example, for every dollar that was spent on Housing First, 54 cents was saved through the reduction in other shelter and health care services.

However, the high service user group (defined as the top 10% of all study participants) presented a different outcome. Average costs per person of non‐study shelter, health and justice services were $56,431 for the treatment group and $30,216 for the intervention group. The difference of $26,215 not only covered the annual cost of $16,825 for the Housing First intervention, it represented a net savings of $9390 per person per year. In other words, for every dollar that was spent on Housing First for these participants, $1.54 was saved through the reduction in other shelter, health and justice services.

Third and similarly, Gulcur (2003) tested a version of the Housing First intervention. The control group was offered similar treatment but with conditions attached, such as attendance at treatment sessions and sobriety. Gulcur (2003) reported significant programme costs attributed to the control group in comparison to the intervention group overall (*F*(1, 173) = 6.1, *p *< .05). There was a small but reliable effect of programme, with participants randomly assigned to the experimental programme costing less. The pattern for costs was similar to the pattern for psychiatric hospitalisation, since costs of days in psychiatric hospitals dwarf costs of other placements. Savings were largest in the first year of the study when the most drastic reduction in hospitalisation occurred; the groups gradually converged thereafter.

Fourth, the full‐service partnership programme (Glimer, 2010) describes the provision of a combination of subsidised permanent housing and team‐based services with a focus on rehabilitation and recovery. In this study, the control group received no treatment. Gilmer (2010) presents the difference in cost for services such as outpatient, inpatient and emergency health services. Justice system costs are also calculated along with the average change in cost across all services. The data presented in this study (Gilmer, 2010) demonstrate that providing safe and secure housing to those are homeless or at risk of homelessness, can reduce costs to expensive services such as those measured. The largest reduction in cost was in inpatient services to the intervention group (‐$3246) in comparison to the control (+$3636). Emergency service costs were also reduced (‐$1305 in the intervention and +$416 in the control group). Housing was provided at an average cost of $3180 per participant, creating an increase in total costs in the intervention group ($8888) over the control ($6771).

Finally, the “Begin a Home” (BAH) intervention (Srebnik et al., 2013) follows a similar structure to that of Housing First in that BAH aims to provide low‐barrier access to permanent housing and a comprehensive team that provides integrated medical, psychiatric and chemical dependency services that are voluntary, intensive and easily accessible. In this study, the control group did not receive any treatment. Srebnik et al. (2013) estimates that BAH participants reduced inpatient and emergency department claims by $1,467,126, jail usage by $10,228, sobering centre usage by $23,856 and medical respite use by $311,420. This created a total estimated reduction in costs of $1,812,630 or $62,504 per person. The difference in service use associated cost reductions between the participants and comparison group of $36,579 appeared to outweigh the programme operating cost of $18,600 per person per year.

## APPENDIX D HOUSING STABILITY ANALYSIS

### Inconsistency of the network

*Quantifying heterogeneity/inconsistency:*

*τ* ^2^ = 0.371, *I* ^2^ = 96.4% with CI: [95.3%, 97.2%]

*Tests of heterogeneity (within designs) and inconsistency (between designs):*

**Table:**

Source	Q	df	p
Total	471.76	17	< 0.001
Within designs	455.15	15	< 0.001
Between designs	16.61	2	< 0.001

*Comparison of Direct and Indirect Estimates, Random Effects Model:*

**Table:**

Comparison	*k*	prop	nma	direct	indir.	Diff	z	p‐value
2:04	0	0	−0.9714	.	−0.9714	.	.	.
2:05	0	0	−0.8316	.	−0.8316	.	.	.
2:06	0	0	−0.8665	.	−0.8665	.	.	.
2:07	0	0	−0.6928	.	−0.6928	.	.	.
2:08	3	1	−1.1043	−1.1043	.	.	.	.
2:09	0	0	−0.4795	.	−0.4795	.	.	.
4:05	0	0	0.1398	.	0.1398	.	.	.
4:06	1	0.55	0.1048	0.2045	−0.0163	0.2208	0.23	0.8218
4:07	0	0	0.2786	.	0.2786	.	.	.
4:08	1	0.61	−0.133	−0.2197	0.001	−0.2208	−0.23	0.8218
4:09	0	0	0.4919	.	0.4919	.	.	.
5:06	0	0	−0.035	.	−0.035	.	.	.
5:07	0	0	0.1388	.	0.1388	.	.	.
5:08	3	1	−0.2728	−0.2728	.	.	.	.
5:09	0	0	0.3521	.	0.3521	.	.	.
6:07	0	0	0.1737	.	0.1737	.	.	.
6:08	0	0	−0.2378	.	−0.2378	.	.	.
6:09	4	0.9	0.3871	0.4095	0.1887	0.2208	0.23	0.8218
7:08	1	0.31	−0.4115	0.2092	−0.6911	0.9003	1.14	0.2561
7:09	3	0.77	0.2133	0.0106	0.9109	−0.9003	−1.14	0.2561
8:09	7	0.86	0.6249	0.6934	0.2014	0.492	0.77	0.4417

*Note*: See Figure 10 for comparison of direct and indirect estimates.

Abbreviations: Comparison, treatment comparison; Diff, Difference between direct and indirect treatment estimates; Direct, estimated treatment effect (SMD) derived from direct evidence; indir., estimated treatment effect (SMD) derived from indirect evidence; *k*, number of studies providing direct evidence; nma, estimated treatment effect (SMD) in network meta‐analysis; prop, direct evidence proportion; *p* value, p‐value of test for disagreement (direct versus indirect); *z, z* value of test for disagreement (direct versus indirect).

*Net rankings of interventions*

**Table:**

Intervention	P‐score (fixed)	P‐score (random)
8	0.9996	0.833
4	0.8067	0.6739
6	0.6075	0.6178
5	0.104	0.576
7	0.3201	0.4727
9	0.0768	0.2643
2	0.5854	0.0623

## APPENDIX E HEALTH OUTCOME ANALYSIS

### Inconsistency of the network

*Quantifying heterogeneity/inconsistency:*

*τ* ^2^ = 0.0.95, *I* ^2^ = 99.1% with CI: [99.0%, 99.3%]

*Tests of heterogeneity (within designs) and inconsistency (between designs):*

**Table:**

Source	Q	df	p
Total	1852.22	16	<.001
Within designs	1795.81	13	<.001
Between designs	56.41	3	<.001

*Comparison of Direct and Indirect Estimates, Random Effects Model:*

**Table:**

comparison	k	prop	nma	direct	indir.	Diff	z	p‐value
2:04	0	0	−0.1934	.	−0.1934	.	.	.
2:05	0	0	−0.4547	.	−0.4547	.	.	.
2:06	0	0	−0.3603	.	−0.3603	.	.	.
2:07	0	0	−0.309	.	−0.309	.	.	.
2:08	2	1	−0.317	−0.317	.	.	.	.
2:09	0	0	−0.0952	.	−0.0952	.	.	.
4:05	0	0	−0.2613	.	−0.2613	.	.	.
4:06	0	0	−0.1669	.	−0.1669	.	.	.
4:07	0	0	−0.1157	.	−0.1157	.	.	.
4:08	1	1	−0.1236	−0.1236	.	.	.	.
4:09	0	0	0.0982	.	0.0982	.	.	.
5:06	1	0.42	0.0944	0.0884	0.0988	−0.0104	−0.03	0.9796
5:07	0	0	0.1457	.	0.1457	.	.	.
5:08	2	0.58	0.1377	0.1992	0.052	0.1472	0.43	0.6649
5:09	1	0.3	0.3595	0.2436	0.4095	−0.1659	−0.45	0.654
6:07	0	0	0.0513	.	0.0513	.	.	.
6:08	0	0	0.0433	.	0.0433	.	.	.
6:09	3	0.8	0.2651	0.2631	0.2734	−0.0104	−0.03	0.9796
7:08	1	0.27	−0.0079	0.261	−0.1066	0.3676	0.99	0.3213
7:09	4	0.82	0.2138	0.1479	0.5155	−0.3676	−0.99	0.3213
8:09	7	0.8	0.2218	0.2766	−0.0015	0.2781	1.05	0.2947

*Note*: See Figure 10 for comparison of direct and indirect estimates.

Abbreviations: Comparison, treatment comparison; Diff, difference between direct and indirect treatment estimates; Direct, estimated treatment effect (SMD) derived from direct evidence; indir., estimated treatment effect (SMD) derived from indirect evidence; *k*, number of studies providing direct evidence; nma, estimated treatment effect (SMD) in network meta‐analysis; prop, direct evidence proportion; *p* value, *p* value of test for disagreement (direct versus indirect); *z, z* value of test for disagreement (direct versus indirect).

*Net rankings of interventions*

**Table:**

Intervention	P‐score (fixed)	P‐score (random)
5	1	0.8215
6	0.8333	0.6716
8	0.6667	0.6157
7	0	0.5947
4	0.2224	0.4297
9	0.4995	0.1974
2	0.2781	0.1693
